# Treatment Effects in Randomized and Nonrandomized Studies of Pharmacological Interventions: A Meta-Analysis

**DOI:** 10.1001/jamanetworkopen.2024.36230

**Published:** 2024-09-27

**Authors:** Maximilian Salcher-Konrad, Mary Nguyen, Jelena Savović, Julian P. T. Higgins, Huseyin Naci

**Affiliations:** 1 Department of Health Policy, London School of Economics and Political Science, London, United Kingdom; 2 World Health Organization Collaborating Centre for Pharmaceutical Pricing and Reimbursement Policies, Pharmacoeconomics Department, Gesundheit Österreich GmbH (GÖG)/Austrian National Public Health Institute, Vienna, Austria; 3 Department of Family and Community Medicine, University of California, San Francisco; 4 Population Health Sciences, Bristol Medical School, University of Bristol, Bristol, United Kingdom; 5 National Institute for Health and Care Research Applied Research Collaboration West, University Hospitals Bristol and Weston National Health Service Foundation Trust, Bristol, United Kingdom

## Abstract

**Importance:**

Randomized clinical trials (RCTs) are widely regarded as the methodological benchmark for assessing clinical efficacy and safety of health interventions. There is growing interest in using nonrandomized studies to assess efficacy and safety of new drugs.

**Objective:**

To determine how treatment effects for the same drug compare when evaluated in nonrandomized vs randomized studies.

**Data Sources:**

Meta-analyses published between 2009 and 2018 were identified in MEDLINE via PubMed and the Cochrane Database of Systematic Reviews. Data analysis was conducted from October 2019 to July 2024.

**Study Selection:**

Meta-analyses of pharmacological interventions were eligible for inclusion if both randomized and nonrandomized studies contributed to a single meta-analytic estimate.

**Data Extraction and Synthesis:**

For this meta-analysis using a meta-epidemiological framework, separate summary effect size estimates were calculated for nonrandomized and randomized studies within each meta-analysis using a random-effects model and then these estimates were compared. The reporting of this study followed the Guidelines for Reporting Meta-Epidemiological Methodology Research and relevant portions of the Preferred Reporting Items for Systematic Reviews and Meta-Analyses (PRISMA) reporting guideline.

**Main Outcome and Measures:**

The primary outcome was discrepancies in treatment effects obtained from nonrandomized and randomized studies, as measured by the proportion of meta-analyses where the 2 study types disagreed about the direction or magnitude of effect, disagreed beyond chance about the effect size estimate, and the summary ratio of odds ratios (ROR) obtained from nonrandomized vs randomized studies combined across all meta-analyses.

**Results:**

A total of 346 meta-analyses with 2746 studies were included. Statistical conclusions about drug benefits and harms were different for 130 of 346 meta-analyses (37.6%) when focusing solely on either nonrandomized or randomized studies. Disagreements were beyond chance for 54 meta-analyses (15.6%). Across all meta-analyses, there was no strong evidence of consistent differences in treatment effects obtained from nonrandomized vs randomized studies (summary ROR, 0.95; 95% credible interval [CrI], 0.89-1.02). Compared with experimental nonrandomized studies, randomized studies produced on average a 19% smaller treatment effect (ROR, 0.81; 95% CrI, 0.68-0.97). There was increased heterogeneity in effect size estimates obtained from nonrandomized compared with randomized studies.

**Conclusions and Relevance:**

In this meta-analysis of treatment effects of pharmacological interventions obtained from randomized and nonrandomized studies, there was no overall difference in effect size estimates between study types on average, but nonrandomized studies both overestimated and underestimated treatment effects observed in randomized studies and introduced additional uncertainty. These findings suggest that relying on nonrandomized studies as substitutes for RCTs may introduce additional uncertainty about the therapeutic effects of new drugs.

## Key Points

**Question:**

How do treatment effects for drugs compare when obtained from nonrandomized vs randomized studies?

**Findings:**

In this meta-analysis of 2746 primary studies in 346 meta-analyses using a meta-epidemiological framework, there was no strong evidence of systematic overestimation or underestimation of treatment effects. However, disagreements between nonrandomized and randomized studies were beyond chance in 15.6% of meta-analyses, and the 2 study types led to different statistical conclusions about the therapeutic effect of drug interventions in 37.6% of meta-analyses.

**Meaning:**

These findings suggest that relying on nonrandomized studies as substitutes for randomized clinical trials may introduce additional uncertainty about the therapeutic effects of new drugs.

## Teaser

This meta-analysis using a meta-epidemiological framework compares treatment effects of pharmacological interventions obtained from randomized clinical trials (RCTs) and nonrandomized studies.

## Introduction

Randomized clinical trials (RCTs), in which participants are randomly assigned to treatments, are widely regarded as the methodological benchmark for assessing the clinical efficacy and safety of drugs.^1,2^ When designed, conducted, analyzed, and reported adequately, RCTs minimize bias and can therefore provide regulatory bodies, payers, clinicians, and patients with robust evidence on what treatments work. In contrast with RCTs, treatment assignment in nonrandomized studies (NRSs) is influenced by the patient, the clinician, or the setting. Despite their higher generalizability, NRSs are more susceptible to bias due to confounding and to selection bias.^3^ Consequently, discrepancies may emerge between the results of RCTs and NRSs.

The internal validity of NRSs has recently attracted renewed interest due to a growing enthusiasm for using NRSs when making decisions about new drugs. Drug regulatory agencies and health technology assessment bodies in the US and Europe are actively exploring the feasibility and validity of utilizing NRSs, including data collected outside of clinical trials (ie, observational data).^4,5,6,7^ While NRSs have traditionally been used as a complement to RCTs, there is interest in potentially substituting or replacing RCTs with well-conducted NRSs.^8^

Previous research^9,10,11,12,13,14,15,16,17,18^ has examined the comparability of treatment effect size estimates between RCTs and NRSs, yielding varied findings. However, the most recent comprehensive review,^12^ encompassing 45 clinical questions and 408 individual studies, was published more than 20 years ago. Most published studies focused on selected therapeutic areas, limiting the generalizability of their findings. Most recently, replication studies for highly selected clinical questions with good data availability have identified a general alignment between RCTs and their nonrandomized emulations, although disagreements in results were observed in approximately one-quarter of the cases.^19^ A comprehensive review of potential discrepancies between treatment effects of RCTs and NRSs is needed. In this study, our primary objective was to assess and compare treatment effects of the same drug when evaluated in NRSs vs RCTs.

## Methods

The study protocol for this meta-analysis using a meta-epidemiological framework was registered on PROSPERO (CRD42018062204). The reporting of this study followed the Guidelines for Reporting Meta-Epidemiological Methodology Research by Murad et al^20^ and relevant portions of the Preferred Reporting Items for Systematic Reviews and Meta-Analyses (PRISMA) reporting guideline.^21^

### Identification of Clinical Questions

We identified clinical questions for which meta-analyses including at least 1 RCT and 1 NRS were conducted to obtain estimates of the effectiveness of pharmacological treatments as defined in the participants, interventions, comparators, and outcomes (PICO) framework. Clinical questions with potentially eligible meta-analyses were identified through 3 sources: (1) a database search in MEDLINE (via PubMed) for existing meta-epidemiological studies comparing RCTs with NRSs, (2) a database search in MEDLINE (via PubMed) for systematic reviews including both RCTs and NRSs, and (3) a review of all systematic reviews indexed in the Cochrane Database of Systematic Reviews that included both RCTs and NRSs. We only included records published from 2009 to 2018 to cover clinical questions from the last decade (our original plan was to cover 2000-2018). Details of the database searches are available in eAppendix 1 in Supplement 1.

We included only clinical questions where RCTs and NRS contributed to a single meta-analytic estimate, following the within–meta-analyses approach for meta-epidemiological studies.^22^ We therefore capitalized on the subject matter expertise of researchers conducting meta-analysis in their area of interest and who judged RCTs and NRSs to be sufficiently similar to each with other with respect to study participants, intervention, comparator, and outcome to provide evidence on a drug’s benefits or harms. Systematic reviews where RCTs and NRS were meta-analyzed separately were excluded.

Potential source systematic reviews containing such meta-analyses, as identified through database searches, were screened at the title and abstract level independently by 2 reviewers (M.S.K. and a research assistant). Conflicting decisions were resolved by consensus. Full texts of remaining records were screened by 1 reviewer (M.N. or M.S.K.), after double screening of a 10% sample of records showed almost perfect agreement (κ = 0.85).

For each included source systematic review, we selected 1 meta-analysis for data extraction. We extracted data for the meta-analysis of the primary outcome. In cases where the meta-analysis of the primary outcome did not include both RCTs and NRSs, we extracted the next most prominently presented outcome with the highest number of contributing RCTs and NRSs. We identified possible double-counting of original studies included in the identified meta-analyses on the basis of unique identifiers.^23^ While original studies were eligible to contribute to several meta-analyses (eg, meta-analyses of the same intervention but measuring different outcomes), within each meta-analysis, only unique individual studies were included.

### Data Extraction

Meta-analysis–level and study-level information were extracted from source systematic reviews using a prespecified spreadsheet by a single researcher (M.N.). We used a guidebook with instructions for each item and data extraction was checked by a second researcher (M.S.K.) for approximately 10% of meta-analyses. Where possible, we used prespecified categories for study design characteristics (eAppendix 2 in Supplement 1).

We based the categorization of study designs on typologies used in previous meta-epidemiological reviews.^13,24^ We distinguished between RCTs and NRSs, where the former was defined by the use of a random sequence to allocate study participants to intervention and control groups, and the latter by the absence of such a random sequence. We relied on the assessment made by the authors of the source reviews whether a study should be categorized as an RCT or NRS.

For NRSs, we further distinguished between experimental and observational designs, a categorization also applied by others.^13,25,26,27^ Experimental NRSs are studies in which the investigator has some control over study conditions, including the allocation of participants into treatment and control groups (eg, clinical trials where the allocation mechanism falls short of true randomization or where allocation is by patient or physician preference). Observational NRSs lack the experimental intention of experimental NRSs, exploiting natural variation in the use of interventions to evaluate patient outcomes.

### Statistical Analysis

#### Main Analysis

All effect size estimates were converted into log odds ratios (ORs) and coded so that an OR less than 1 indicated a beneficial effect of the drug under investigation. For meta-analyses reporting continuous outcomes, we first converted these into standardized mean differences (SMDs)^28^ and then to ORs.^29^ For meta-analyses with active comparators, we identified which drug was considered experimental through the descriptions provided by the authors of the source review or through web searches in cases where this could not be determined with certainty from the source review.

In descriptive analyses, we first plotted the summary estimates for NRSs and RCTs conducted for the same clinical question and reported the number of meta-analyses for which the NRS and RCT effect size estimates, respectively, were more favorable. Within each meta-analysis, we calculated the summary estimates and 95% CIs of NRSs and RCTs, respectively, using a random-effects Hartung-Knapp-Sidik-Jonkman meta-analysis model to take into account between-study heterogeneity.^30,31^

We reported 4 measures of discrepancy. First, we reported the frequency of substantial disagreement, operationalized as the summary OR obtained from one type of study being twice as favorable as the other (ie, OR obtained from one study type was at most one-half the OR obtained from the other study type).^12^ We also considered alternative cutoff values (differences in summary OR by 50% and 10%). Second, we reported the frequency of discrepancies in the summary logOR being beyond what would be expected by chance alone at the 5% significance level.^12^ We compared the summary logORs for the NRS and RCT for each meta-analysis using the equation:

log_ROR_ = log(OR_NRS_) − log(OR_RCT_),

where ROR is the ratio of odds ratios, and then computed a 95% CI using standard error (SE) of logROR using the equation:
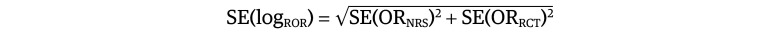
and compared these CIs with the null value of logROR = 0. Third, we reported the frequency of meta-analyses for which the summary estimates of NRSs and RCTs, respectively, led to different statistical conclusions. A different statistical conclusion was considered to be reached if one study type produced a meta-analytic result with 95% CI excluding an OR of 1 in a particular direction and the other study type did not. Contradictory treatment effects were considered to occur when a 95% CI for the meta-analytic OR for NRSs was entirely less than 1 while that for the meta-analytic OR for an RCTs was entirely greater than 1, or vice versa. This analysis did not account for differences in sample sizes between the 2 study types. Fourth, in the main, prespecified analysis, we quantified discrepancies between NRSs and RCTs through a 2-stage meta-analysis to obtain RORs for treatment effects obtained from NRSs vs RCTs.^32^ The analysis was implemented in a bayesian framework, with noninformative prior distributions for the discrepancy of treatment effects between NRS and RCTs.^33^ We also quantified the variation of discrepant treatment effects between NRS and RCT results across meta-analyses using the between–meta-analysis SD in discrepancies (φ) and the variation of discrepancies across studies within meta-analyses using the between-study SD in discrepancies (κ).^34,35^ These measures indicate variation in effect size estimates obtained from different study designs; higher values indicate a wider spread in the magnitude of discrepancies between the 2 study types across meta-analyses (φ) and across individual studies within meta-analyses (κ).

Other measures for assessing discrepancies in treatment effects exist, such as correlation and concordance coefficients and the absolute ROR.^10,12,14,15,17,34,36,37,38^ We focused on measures that we deemed important from a clinical or regulatory decision-making perspective (ie, that provide estimates of both absolute and relative discrepancies, potential differences in statistical conclusions drawn, and direction of deviation).

Analyses were implemented in Stata version 13.1 (StataCorp) and WinBUGS version 1.4.3 (Imperial College and Medical Research Council). Analysis was conducted from October 2019 to July 2024.

#### Subgroup and Sensitivity Analyses

Subgroup analyses were conducted for prespecified characteristics at the meta-analysis level and study level. Additional subgroup analysis to explore heterogeneity in the discrepancy in treatment effects in RCTs vs NRSs was conducted by data source of NRSs, type of control in NRSs, therapeutic area, how well matched RCTs and NRSs included in a meta-analysis were, and methodological quality of source meta-analyses. Study-level characteristics were often not reported in detail in source meta-analyses, resulting in small sample sizes for most subgroups. We therefore only report the results of subgroup analyses for selected characteristics (details in eAppendix 3 in Supplement 1). In a post hoc sensitivity analysis, we restricted our sample to meta-analyses where NRSs were published before the first RCT.

## Results

**Figure 1.**
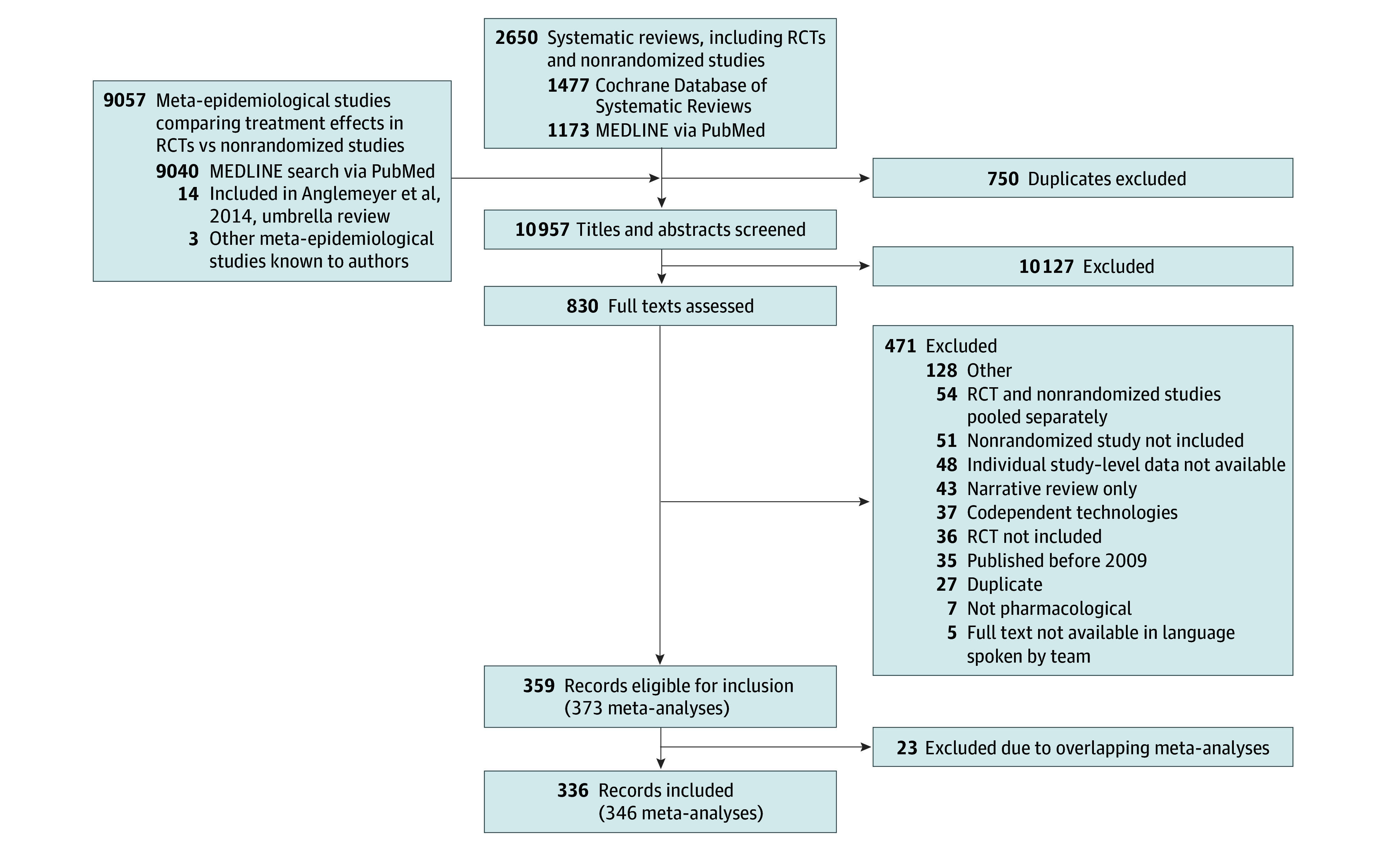
Flowchart of Selection of Meta-Analyses for Meta-Epidemiological Study RCT indicates randomized clinical trial.

A total of 10 957 records were screened at the title and abstract level, and 830 were reviewed in full, resulting in a total of 336^14,39,40,41,42,43,44,45,46,47,48,49,50,51,52,53,54,55,56,57,58,59,60,61,62,63,64,65,66,67,68,69,70,71,72,73,74,75,76,77,78,79,80,81,82,83,84,85,86,87,88,89,90,91,92,93,94,95,96,97,98,99,100,101,102,103,104,105,106,107,108,109,110,111,112,113,114,115,116,117,118,119,120,121,122,123,124,125,126,127,128,129,130,131,132,133,134,135,136,137,138,139,140,141,142,143,144,145,146,147,148,149,150,151,152,153,154,155,156,157,158,159,160,161,162,163,164,165,166,167,168,169,170,171,172,173,174,175,176,177,178,179,180,181,182,183,184,185,186,187,188,189,190,191,192,193,194,195,196,197,198,199,200,201,202,203,204,205,206,207,208,209,210,211,212,213,214,215,216,217,218,219,220,221,222,223,224,225,226,227,228,229,230,231,232,233,234,235,236,237,238,239,240,241,242,243,244,245,246,247,248,249,250,251,252,253,254,255,256,257,258,259,260,261,262,263,264,265,266,267,268,269,270,271,272,273,274,275,276,277,278,279,280,281,282,283,284,285,286,287,288,289,290,291,292,293,294,295,296,297,298,299,300,301,302,303,304,305,306,307,308,309,310,311,312,313,314,315,316,317,318,319,320,321,322,323,324,325,326,327,328,329,330,331,332,333,334,335,336,337,338,339,340,341,342,343,344,345,346,347,348,349,350,351,352,353,354,355,356,357,358,359,360,361,362,363,364,365,366,367,368,369,370,371,372,373^ included records (Figure 1). These 336 records contributed 346 unique meta-analyses (2 meta-epidemiological studies^14,174^ contributed more than 1 meta-analysis), with 2746 contributing individual studies (median [range] 3 [1-92] RCTs with a median [range] 100 [5-235 600] participants and median [range] 2 [1-44] NRSs with a median [range] 195 [6-2 145 593] participants per meta-analysis). Characteristics of included meta-analyses are presented in eTable 1 in Supplement 1 and summarized in the Table.

**Table.** Characteristics of Meta-Analyses Including Both Nonrandomized and Randomized Studies

Characteristic	Meta-analyses, No. (%) (N =346)
Comparator	
Active	94 (27.2)
Placebo or no treatment	226 (65.3)
Both active and placebo-controlled studies	26 (7.5)
Outcome type^a^	
Mortality	59 (17.1)
Other objective outcome	158 (45.7)
Subjective outcome	126 (36.4)
Different types of outcomes	3 (0.9)
Therapeutic area by WHO ATC first level categorization	
Anti-infective for systemic use	66 (19.1)
Blood and blood forming organs	64 (18.5)
Cardiovascular system	45 (13.0)
Antineoplastic and immuno-modulating agents	43 (12.4)
Nervous system	27 (7.8)
Alimentary tract and metabolism	23 (6.6)
Systemic hormonal preparations	19 (5.5)
Genito-urinary system and sex hormones	14 (4.0)
Other categories combined	45 (13.0)
Risk of bias across NRSs in a meta-analysis^b^	
Low median risk of bias	96 (27.7)
Moderate median risk of bias	61 (17.6)
High median risk of bias	123 (35.5)
No risk of bias information	66 (19.1)
Risk of bias across RCTs in a meta-analysis^b^	
Low median risk of bias	90 (26.0)
Moderate median risk of bias	95 (27.5)
High median risk of bias	103 (29.8)
No risk of bias information	58 (16.8)
Median publication year of studies included in a meta-analyses	
Before 2000	56 (16.2)
2000-2009	131 (37.9)
2010 and later	159 (46.0)
Matching quality of RCTs and NRSs in a meta-analysis^c^	
High (score of 10-12 of 12)	111 (32.1)
Moderate (score of 7-9 of 12)	166 (48.0)
Low (score of 4-6 of 12)	69 (19.9)
Timing of evidence generation	
NRS published before first RCT	146 (42.2)
First RCT published before NRS	169 (48.8)
First NRS and first RCT published in the same year	31 (9.0)

Abbreviations: NRS, nonrandomized study; RCT, randomized clinical trial; WHO ATC, World Health Organization Anatomical Therapeutic Chemical classification system.

^a^ Outcomes were categorized according to the extent to which their assessment could be influenced by investigators’ judgment.^374^ For composite outcomes, we used the most subjective component.

^b^ The proportion of meta-analyses for which the median of the risk of bias scores of NRSs or RCTs included in that meta-analysis was low, moderate, or high. Risk of bias assessments were extracted from source meta-analyses and standardized as low, moderate, or high.

^c^ The proportion of meta-analyses for which the quality of the matching between NRSs and RCTs included in the meta-analysis was deemed high, moderate, or low according to how closely aligned each of the 4 PICO components (participants, intervention, comparator, outcome) were between NRSs and RCTs. A score from 1 to 3 was assigned for each of the 4 PICO components according to how well NRSs and RCTs included in the same meta-analysis were matched.

**Figure 2.**
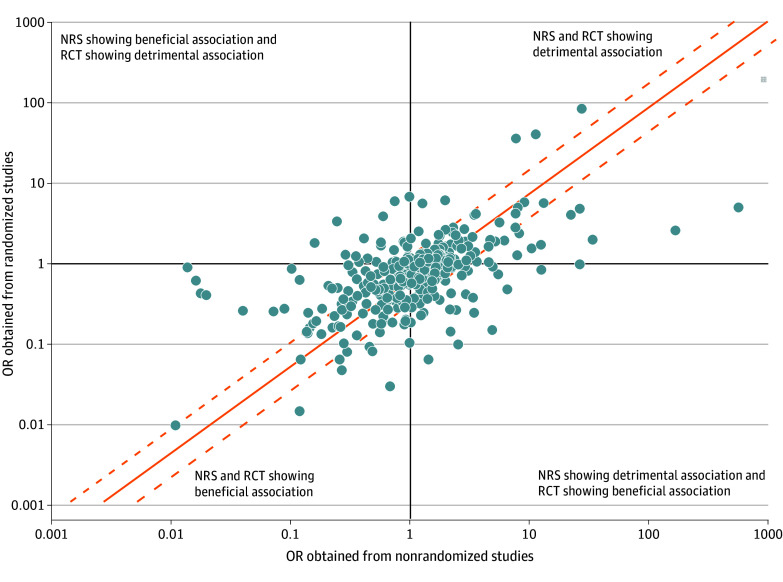
Agreement of Summary Effect Size Estimates Obtained From Randomized and Nonrandomized Studies for 346 Clinical Questions Each circle shows the summary odds ratio (OR) obtained from a meta-analysis of randomized clinical trials (RCTs; vertical axis) and nonrandomized studies (NRSs; horizonal axis) for 1 clinical question. An OR less than 1 indicates a beneficial effect. The solid orange line indicates perfect agreement (exact same summary OR obtained from randomized and nonrandomized studies) and the dashed orange lines indicate substantial disagreement (OR obtained from randomized studies is at most one-half of the OR obtained from nonrandomized studies, or vice versa). Results for alternative cutoff values for substantial disagreement are provided in eTable 2 in Supplement 1. Circles in the upper left quadrant show meta-analyses where NRS evidence indicates a beneficial effect (summary OR <1) and RCT evidence a detrimental effect (summary OR >1), and circles in the bottom right quadrant show meta-analyses where NRS evidence indicates a detrimental effect (summary OR >1) and RCT evidence a beneficial effect (summary OR <1). Circles in the upper right quadrant show meta-analyses where both NRS and RCT evidence indicate a detrimental effect; circles above the solid orange line indicate a larger detrimental effect size in RCTs and circles below the solid orange line indicate a larger detrimental effect size in NRSs. Circles in the bottom left quadrant show meta-analyses where both NRS and RCT evidence indicate a beneficial effect; circles above the solid orange line indicate a larger beneficial effect size in NRS and circles below the solid orange line indicate a larger beneficial effect size in RCTs.

Discrepancies between treatment effects are displayed in Figure 2, which shows the effect size estimates obtained from RCTs and NRSs for all 346 meta-analyses. NRSs gave a more favorable effect (ie, a lower summary OR) for 186 meta-analyses (53.8%), and RCTs gave a more favorable effect for 158 meta-analyses (45.7%). Results for all measures of discrepancy are summarized in the eTable 2 in Supplement 1. For 121 meta-analyses (35.0%), the OR obtained from one study type was twice as large or more (or one-half the OR or less) than the other, including 65 (18.8% of all meta-analyses) where NRSs indicated a substantially more beneficial effect and 56 (16.2%) where RCTs indicated a substantially more beneficial effect (Figure 2). Disagreement between study types was beyond chance for 54 meta-analyses (15.6%), including 30 (8.7%) where the OR obtained from NRSs was more beneficial, and 24 (6.9%) where the OR obtained from RCTs was more beneficial. In a subgroup analysis that only included experimental NRSs, the OR from one study type was twice as favorable as the other for 55 meta-analyses (45.1% of all meta-analyses including experimental NRSs), including 36 (29.5%) where the OR obtained from experimental NRSs was one-half the OR of RCTs or less. Disagreement between study types was beyond chance for 31 meta-analyses (25.4%) with experimental NRS. The subgroup analysis for observational studies showed lower frequencies of discrepancies (eTable 2 in Supplement 1).

**Figure 3.**
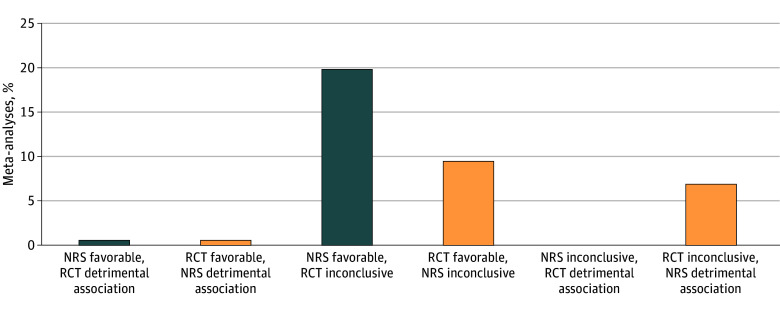
Discrepancies in Statistical Conclusions About Therapeutic Benefit of Pharmacological Interventions Based on Evidence Obtained From Nonrandomized Studies (NRSs) or Randomized Clinical Trials (RCTs) The figure shows proportions of meta-analyses based on the statistical conclusions about the existence of a therapeutic benefit drawn from NRS or RCT evidence. A favorable or detrimental effect was deemed to exist if the 95% CI of the summary odds ratio did not include 1. Evidence was considered inconclusive if the 95% CI of the summary odds ratio included 1.

RCTs and NRSs led to different statistical conclusions about the therapeutic benefit of pharmacological interventions in 130 meta-analyses (37.6%) and 216 (62.4%) reached the same statistical conclusion, based on comparing 95% CIs around the OR from either study type with a null effect (Figure 3). In 69 meta-analyses (19.9%), NRSs showed a favorable effect while evidence obtained from RCTs was inconclusive and in 33 meta-analyses (9.5%), RCTs showed a favorable effect while the NRS evidence was inconclusive. Contradictory treatment effects were observed in 4 meta-analyses (1.2%).

**Figure 4.**
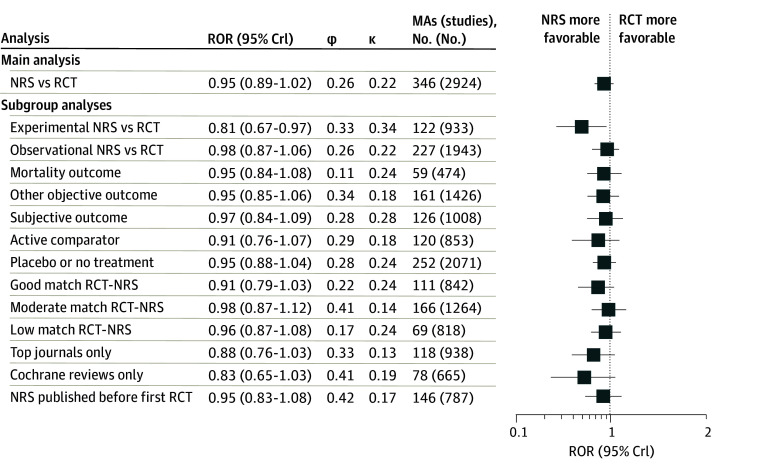
Results of Meta–Meta-Analytic Comparison The figure shows the ratio of odds ratios (ROR) comparing effect size estimates obtained from nonrandomized studies (NRSs) with effect size estimates obtained from randomized clinical trials (RCTs) and heterogeneity parameters (φ, between–meta-analysis heterogeneity; κ, increase in within–meta-analysis heterogeneity). Results are shown for all meta-analyses, followed by subgroup analyses by type of NRS, different types of outcomes, types of comparators, matching quality of RCTs and NRSs in the same meta-analysis, and high-quality publications. MA indicates meta-analyses.

In the main analysis, there was no evidence of a difference between effect size estimates obtained from NRSs vs RCTs on average when combining discrepancies across all 346 meta-analyses (ROR, 0.95; 95% credible interval [CrI], 0.89-1.02) (Figure 4). In subgroup analyses, effect size estimates obtained from experimental NRSs were more favorable compared with RCTs (ROR, 0.81; 95% CrI, 0.68-0.97), overestimating RCT estimates by 19%, while no difference was observed between observational NRS and RCTs (ROR, 0.98; 95% CrI, 0.87-1.06).

Variation in the discrepancy of treatment effects was present between studies within meta-analyses (κ = 0.22) and between meta-analyses (φ = 0.26). Variation between meta-analyses was reduced for meta-analyses measuring mortality (φ = 0.11) compared with other objective outcomes (φ = 0.34) or subjective outcomes (φ = 0.28). There were no systematic differences in between-meta-analysis variation (φ) or within-meta-analysis variation (κ) for the other characteristics at meta-analysis level.

Study-level data regarding analytical methods and data sources used in NRSs were only available for a subset of meta-analyses. Between-meta-analysis variation (φ) and within-meta-analysis variation (κ) were reduced for studies using propensity score methods compared with other analytical methods (eFigure in Supplement 1).

In 146 meta-analyses (42.2%), the first NRS was published before the first RCT. In this subset of meta-analyses, findings were consistent with the overall sample (eTable 2 in Supplement 1). In 53 of the 146 meta-analyses (36.3%), the summary OR was twice as favorable for one study type vs the other; in 31 meta-analyses (21.2%), the discrepancy in summary OR was beyond chance, while 50 (34.2%) reached different statistical conclusions and the ROR was 0.95 (95% CrI, 0.83-1.08) (Figure 4).

## Discussion

This meta-analysis of 346 clinical questions using a meta-epidemiological framework did not uncover any systematic underestimation or overestimation of treatment effects in NRSs when compared with RCTs. However, this overall finding masks substantial variability in the observed differences between treatment effects derived from the 2 study types. A considerable number of meta-analyses exhibited discrepancies in effect size estimates, with some cases showing effect size estimates differing by a factor of 2 or more. Estimates of the variation in discrepancies show that decision-makers face uncertainty around both the direction and magnitude of potential disagreement between RCTs and NRSs; NRSs both overestimated and underestimated treatment effects observed in randomized studies.

Our study extends previous research investigating the comparability of treatment effects derived from RCTs and NRSs.^18^ In particular, it provides findings across a broad range of therapeutic areas, reflecting how NRSs were designed and implemented for the clinical questions included and quantifies uncertainty associated with treatment effects derived from NRSs. Previous meta-epidemiological reviews yielded mixed results,^9,10,11,12,13^ with varying factors such as outcome types,^24^ study timing,^14^ and analytical methods in NRSs contributing to discrepancies across reviews.^15,18,375,376^ In our study, 37.6% of meta-analyses reached different statistical conclusions regarding the effectiveness of a drug depending on the type of study design considered, and 62.4% reached the same statistical conclusion. This finding broadly aligns with a recent study^19^ that sought to emulate highly selected RCTs using administrative data, yielding concordant conclusions for 56% of emulated trials. Our approach was different from this study^19^ and other observational studies^377^ aiming to emulate RCTs by design using the target trial approach. By applying strict criteria to emulate RCTs, these observational studies aim to obtain the same estimand of effectiveness as the target trial. Other NRSs do not necessarily aim to replicate RCTs, and discrepancies in effect size estimates may reflect differences in study design, implementation, and populations. From a decision-maker’s perspective, what matters is the availability of clinical evidence; in situations with uncertainty about the effectiveness of a treatment, NRSs of any design are likely to inform decision-making. Target trial emulation studies apply advanced methodological standards, but there are important data limitations to implement them.^378^ While they are becoming more common,^379^ they represent a small subset of all NRSs evaluating treatment effects. It is therefore important to understand how the body of evidence from NRSs overall compares with evidence obtained from RCTs.

We also provide novel evidence on how different types of study designs, analytical methods, and data used in NRSs perform when compared against RCTs. We found that effect size estimates obtained from experimental NRSs were systematically more favorable than those obtained from RCTs (overestimating RCT estimates by 19%). Experimental NRSs share important validity traits with RCTs, such as a controlled environment for administering the treatment and strict participant inclusion criteria. Nevertheless, the absence of random participant allocation in these studies can introduce bias through confounding. Experimental NRSs showed at least twice as favorable treatment effects as RCTs for 45.1% of meta-analyses.

Our study has important policy implications. NRSs are playing an increasingly important role in influencing decisions about the approval and reimbursement of new drugs.^380,381,382,383^ Between 2015 and 2017, approximately 18% of new drugs gained approval in the US based on NRSs, up from just 6% between 1995 and 1997.^384^ In draft guidance, the US Food and Drug Administration FDA names observational data as potentially suitable evidence for drug approval, replacing the previously used standard of 2 independent clinical studies.^385^ It is therefore important to understand the benefits and risks of relying on NRSs for the evaluation of new drugs. While we found overall no systematic difference in treatment effects obtained from randomized and observational studies, there was considerable disagreement about therapeutic benefit (eTable 2 in Supplement 1).

Our study has implications for practice. Although RCTs are the mainstay of clinical practice guidelines, there are valid concerns about their cost and complexity.^386^ RCTs may also be at high risk of bias due to problems with their design, conduct, analysis, and reporting.^387^ Despite these concerns, our findings underline their importance because the conclusions about a drug’s effect may differ when based on NRSs. In our study, the statistical conclusions about a drug’s treatment effect were different for almost 4 in 10 clinical questions. In the past, medical reversals occurred because RCTs provided conclusive evidence about the benefits and harms of long-standing medical practices that were based on evidence obtained from NRSs.^388,389,390,391,392^ Yet, there appears to be a limited effort to simplify the design and conduct of RCTs. As the push toward NRSs gains more traction, it could potentially impede the necessary progress required to improve the feasibility of RCTs.^393^

### Limitations

This study has limitations. This is an observational study which limits causal interpretation of results.^22^ We included 346 distinct clinical questions that were the subject of meta-analyses published from 2009 to 2018. While this represents, to our knowledge, the largest sample of clinical questions in a meta-epidemiological study comparing RCTs and NRSs, more recent clinical questions, in particular those relating to COVID-19,^17^ were not included.

We included only meta-analyses where researchers combined both RCTs and NRSs in the same meta-analysis. While the 2 designs may not study the same estimand, the fact that they are pooled in the same meta-analysis suggests that the researchers considered them both to provide relevant evidence for decision-makers about whether the treatment is effective or harmful. It is therefore important to understand how their effect size estimates compare. The methodological decision to include meta-analyses where RCTs and NRSs were combined likely resulted in a sample more representative of clinical questions with overall limited levels of evidence (otherwise, only RCTs would be expected to be included in a meta-analysis). Including both study types in the same meta-analysis may also reflect limited methodological understanding of the authors of source meta-analyses, but our conclusions did not change when restricting our sample to meta-analyses conducted by Cochrane groups or those published in high-impact journals. Excluding clinical questions where researchers determined that there were substantial differences between the 2 study types—possibly due to observed differences in results—may have resulted in an underestimation of the true difference between treatment effects obtained from RCTs and NRSs.

## Conclusions

In this meta-analysis using a meta-epidemiological framework, we found substantial disagreements between nonrandomized and randomized studies about the magnitude of effect and statistical conclusions about the therapeutic effect of pharmacological interventions for a large subset of clinical questions. While there was overall no systematic difference in effect size estimates obtained from NRSs vs RCTs, experimental NRSs studies produced 19% larger treatment effects compared with RCTs. Our findings suggest that caution is warranted when relying on NRSs as substitutes for RCTs.

## Notes

Supplement 1. **eAppendix 1.** Search Strategy **eAppendix 2.** Extracted Data From Source Meta-Analyses **eAppendix 3.** Subgroup Analyses
eReferences.
**eTable 1. **Characteristics of Included Meta-Analyses **eTable 2.** Results for Measures of Discrepancy Between Nonrandomized Studies and RCTs **eFigure.** Results From Additional Subgroup Analyses for Study-Level Characteristics

Supplement 2.
Data Sharing Statement

## References

[1] International Council for Harmonisation of Technical Requirements for Pharmaceuticals for Human Use. ICH harmonised guideline: general considerations for clinical studies E8(R1). October 6, 2021. Accessed September 3, 2023. https://database.ich.org/sites/default/files/E8-R1_Guideline_Step4_2021_1006.pdf

[2] Bothwell LE, Greene JA, Podolsky SH, Jones DS. Assessing the gold standard–lessons from the history of RCTs. N Engl J Med. 2016;374(22):2175-2181. doi:10.1056/NEJMms1604593 27248626

[3] Sterne JA, Hernán MA, Reeves BC, . ROBINS-I: a tool for assessing risk of bias in non-randomised studies of interventions. BMJ. 2016;355:i4919. doi:10.1136/bmj.i4919 27733354 PMC5062054

[4] Kesselheim AS, Avorn J. New “21st Century Cures” legislation: speed and ease vs science. JAMA. 2017;317(6):581-582. doi:10.1001/jama.2016.20640 28056124

[5] European Medicines Agency. Real-world evidence framework to support EU regulatory decision-making: report on the experience gained with regulator-led studies from September 2021 to February 2023. Amsterdam: European Medicines Agency. 2023. Accessed September 15, 2023. https://www.ema.europa.eu/en/documents/report/real-world-evidence-framework-support-eu-regulatory-decision-making-report-experience-gained-regulator-led-studies-september-2021-february-2023_en.pdf

[6] European Commission. Proposal for a regulation of the European parliament and of the council laying down union procedures for the authorisation and supervision of medicinal products for human use and establishing rules governing the European Medicines Agency, amending regulation (EC) No. 1394/2007 and regulation (EU) No. 536/2014 and repealing regulation (EC) No 726/2004, regulation (EC) No 141/2000 and regulation (EC) No 1901/2006. April 26, 2023. Accessed August 23, 2024. https://eur-lex.europa.eu/legal-content/EN/TXT/?uri=CELEX%3A52023PC0193

[7] National Institute for Health and Care Excellence. NICE real-world evidence framework. June 23, 2022. Accessed August 6, 2023. http://www.nice.org.uk/corporate/ecd9

[8] Franklin JM, Liaw KL, Iyasu S, Critchlow CW, Dreyer NA. Real-world evidence to support regulatory decision making: new or expanded medical product indications. Pharmacoepidemiol Drug Saf. 2021;30(6):685-693. doi:10.1002/pds.5222 33675248

[9] Benson K, Hartz AJ. A comparison of observational studies and randomized, controlled trials. N Engl J Med. 2000;342(25):1878-1886. doi:10.1056/NEJM200006223422506 10861324

[10] Mc Cord KA, Ewald H, Agarwal A, . Treatment effects in randomised trials using routinely collected data for outcome assessment versus traditional trials: meta-research study. BMJ. 2021;372(450):n450. doi:10.1136/bmj.n450 33658187 PMC7926294

[11] Concato J, Shah N, Horwitz RI. Randomized, controlled trials, observational studies, and the hierarchy of research designs. N Engl J Med. 2000;342(25):1887-1892. doi:10.1056/NEJM200006223422507 10861325 PMC1557642

[12] Ioannidis JP, Haidich AB, Pappa M, . Comparison of evidence of treatment effects in randomized and nonrandomized studies. JAMA. 2001;286(7):821-830. doi:10.1001/jama.286.7.821 11497536

[13] Deeks JJ, Dinnes J, D’Amico R, ; International Stroke Trial Collaborative Group; European Carotid Surgery Trial Collaborative Group. Evaluating non-randomised intervention studies. Health Technol Assess. 2003;7(27):iii-x, 1-173. doi:10.3310/hta7270 14499048

[14] Hemkens LG, Contopoulos-Ioannidis DG, Ioannidis JP. Agreement of treatment effects for mortality from routinely collected data and subsequent randomized trials: meta-epidemiological survey. BMJ. 2016;352:i493. doi:10.1136/bmj.i493 26858277 PMC4772787

[15] Ewald H, Ioannidis JPA, Ladanie A, Mc Cord K, Bucher HC, Hemkens LG. Nonrandomized studies using causal-modeling may give different answers than RCTs: a meta-epidemiological study. J Clin Epidemiol. 2020;118:29-41. doi:10.1016/j.jclinepi.2019.10.012 31704350

[16] Mathes T, Rombey T, Kuss O, Pieper D. No inexplicable disagreements between real-world data-based nonrandomized controlled studies and randomized controlled trials were found. J Clin Epidemiol. 2021;133:1-13. doi:10.1016/j.jclinepi.2020.12.019 33359322

[17] Moneer O, Daly G, Skydel JJ, . Agreement of treatment effects from observational studies and randomized controlled trials evaluating hydroxychloroquine, lopinavir-ritonavir, or dexamethasone for covid-19: meta-epidemiological study. BMJ. 2022;377:e069400. doi:10.1136/bmj-2021-069400 35537738 PMC9086409

[18] Toews I, Anglemyer A, Nyirenda JL, . Healthcare outcomes assessed with observational study designs compared with those assessed in randomized trials: a meta-epidemiological study. Cochrane Database Syst Rev. 2024;1(1):MR000034. 38174786 10.1002/14651858.MR000034.pub3 PMC10765475

[19] Wang SV, Schneeweiss S, Franklin JM, ; RCT-DUPLICATE Initiative. Emulation of randomized clinical trials with nonrandomized database analyses: results of 32 clinical trials. JAMA. 2023;329(16):1376-1385. doi:10.1001/jama.2023.4221 37097356 PMC10130954

[20] Murad MH, Wang Z. Guidelines for reporting meta-epidemiological methodology research. Evid Based Med. 2017;22(4):139-142. doi:10.1136/ebmed-2017-110713 28701372 PMC5537553

[21] Page MJ, McKenzie JE, Bossuyt PM, . The PRISMA 2020 statement: an updated guideline for reporting systematic reviews. BMJ. 2021;372(71):n71. doi:10.1136/bmj.n71 33782057 PMC8005924

[22] Moustgaard H, Jones HE, Savović J, . Ten questions to consider when interpreting results of a meta-epidemiological study-the MetaBLIND study as a case. Res Synth Methods. 2020;11(2):260-274. doi:10.1002/jrsm.1392 31851427

[23] Savović J, Harris RJ, Wood L, . Development of a combined database for meta-epidemiological research. Res Synth Methods. 2010;1(3-4):212-225. doi:10.1002/jrsm.18 26061467

[24] Anglemyer A, Horvath HT, Bero L. Healthcare outcomes assessed with observational study designs compared with those assessed in randomized trials. Cochrane Database Syst Rev. 2014;2014(4):MR000034. doi:10.1002/14651858.MR000034.pub2 24782322 PMC8191367

[25] Grimes DA, Schulz KF. An overview of clinical research: the lay of the land. Lancet. 2002;359(9300):57-61. doi:10.1016/S0140-6736(02)07283-5 11809203

[26] Reeves BC, Wells GA, Waddington H. Quasi-experimental study designs series-paper 5: a checklist for classifying studies evaluating the effects on health interventions-a taxonomy without labels. J Clin Epidemiol. 2017;89:30-42. doi:10.1016/j.jclinepi.2017.02.016 28351692 PMC5669452

[27] Innovative Medicines Initiative. RWE Navigator: Generating Real-World Evidence. Accessed August 23, 2024. https://rwe-navigator.eu/?page_id=849

[28] Higgins J, Deeks J. Selecting studies and collecting data. In: Higgins J, Green S, eds. Cochrane Handbook for Systematic Reviews of Interventions Version 5.1.0. The Cochrane Collaboration; 2011. Accessed August 23, 2024. https://handbook-5-1.cochrane.org/chapter_7/7_selecting_studies_and_collecting_data.htm

[29] Chinn S. A simple method for converting an odds ratio to effect size for use in meta-analysis. Stat Med. 2000;19(22):3127-3131. doi:10.1002/1097-0258(20001130)19:22<3127::AID-SIM784>3.0.CO;2-M 11113947

[30] Borenstein M, Hedges LV, Higgins JP, Rothstein HR. A basic introduction to fixed-effect and random-effects models for meta-analysis. Res Synth Methods. 2010;1(2):97-111. doi:10.1002/jrsm.12 26061376

[31] IntHout J, Ioannidis JP, Borm GF. The Hartung-Knapp-Sidik-Jonkman method for random effects meta-analysis is straightforward and considerably outperforms the standard DerSimonian-Laird method. BMC Med Res Methodol. 2014;14:25. doi:10.1186/1471-2288-14-25 24548571 PMC4015721

[32] Sterne JAC, Jüni P, Schulz KF, Altman DG, Bartlett C, Egger M. Statistical methods for assessing the influence of study characteristics on treatment effects in ‘meta-epidemiological’ research. Stat Med. 2002;21(11):1513-1524. doi:10.1002/sim.1184 12111917

[33] Welton NJ, Ades AE, Carlin JB, Altman DG, Sterne JAC. Models for potentially biased evidence in meta-analysis using empirically based priors. J R Stat Soc Ser A Stat Soc. 2009;172(1):119-136. doi:10.1111/j.1467-985X.2008.00548.x

[34] Savović J, Jones HE, Altman DG, . Influence of reported study design characteristics on intervention effect estimates from randomized, controlled trials. Ann Intern Med. 2012;157(6):429-438. doi:10.7326/0003-4819-157-6-201209180-00537 22945832

[35] Savović J, Turner RM, Mawdsley D, . Association between risk-of-bias assessments and results of randomized trials in Cochrane Reviews: the ROBES meta-epidemiologic study. Am J Epidemiol. 2018;187(5):1113-1122. doi:10.1093/aje/kwx344 29126260 PMC5928453

[36] Dahabreh IJ, Sheldrick RC, Paulus JK, . Do observational studies using propensity score methods agree with randomized trials? A systematic comparison of studies on acute coronary syndromes. Eur Heart J. 2012;33(15):1893-1901. doi:10.1093/eurheartj/ehs114 22711757 PMC3409422

[37] Soni PD, Hartman HE, Dess RT, . Comparison of population-based observational studies with randomized trials in oncology. J Clin Oncol. 2019;37(14):1209-1216. doi:10.1200/JCO.18.01074 30897037 PMC7186578

[38] Moustgaard H, Clayton GL, Jones HE, . Impact of blinding on estimated treatment effects in randomised clinical trials: meta-epidemiological study. BMJ. 2020;368:l6802. doi:10.1136/bmj.l6802 31964641 PMC7190062

[39] Abolhassani H, Sadaghiani MS, Aghamohammadi A, Ochs HD, Rezaei N. Home-based subcutaneous immunoglobulin versus hospital-based intravenous immunoglobulin in treatment of primary antibody deficiencies: systematic review and meta analysis. J Clin Immunol. 2012;32(6):1180-1192. doi:10.1007/s10875-012-9720-1 22730009

[40] Afolabi BB, Lesi FE. Regional versus general anaesthesia for caesarean section. Cochrane Database Syst Rev. 2012;10:CD004350. doi:10.1002/14651858.CD004350.pub3 23076903 PMC12009660

[41] Agarwal N, Jain A, Mahmoud AN, . Safety and efficacy of dual versus triple antithrombotic therapy in patients undergoing percutaneous coronary intervention. Am J Med. 2017;130(11):1280-1289. doi:10.1016/j.amjmed.2017.03.057 28460853

[42] Agarwal N, Mahmoud AN, Patel NK, . Meta-analysis of aspirin versus dual antiplatelet therapy following coronary artery bypass grafting. Am J Cardiol. 2018;121(1):32-40. doi:10.1016/j.amjcard.2017.09.022 29122278

[43] Alfirevic Z, Kelly AJ, Dowswell T. Intravenous oxytocin alone for cervical ripening and induction of labour. Cochrane Database Syst Rev. 2009;2009(4):CD003246. doi:10.1002/14651858.CD003246.pub2 19821304 PMC4164045

[44] Allen SJ, Martinez EG, Gregorio GV, Dans LF. Probiotics for treating acute infectious diarrhoea. Cochrane Database Syst Rev. 2010;2010(11):CD003048. 21069673 10.1002/14651858.CD003048.pub3 PMC6532699

[45] Ampuero J, Reddy KR, Romero-Gomez M. Hepatitis C virus genotype 3: Meta-analysis on sustained virologic response rates with currently available treatment options. World J Gastroenterol. 2016;22(22):5285-5292. doi:10.3748/wjg.v22.i22.5285 27298572 PMC4893476

[46] An T, Hao J, Sun S, . Efficacy of statins for osteoporosis: a systematic review and meta-analysis. Osteoporos Int. 2017;28(1):47-57. doi:10.1007/s00198-016-3844-8 27888285

[47] Andia I, Latorre PM, Gomez MC, Burgos-Alonso N, Abate M, Maffulli N. Platelet-rich plasma in the conservative treatment of painful tendinopathy: a systematic review and meta-analysis of controlled studies. Br Med Bull. 2014;110(1):99-115. doi:10.1093/bmb/ldu007 24795364

[48] Antoniou GA, Fisher RK, Georgiadis GS, Antoniou SA, Torella F. Statin therapy in lower limb peripheral arterial disease: systematic review and meta-analysis. Vascul Pharmacol. 2014;63(2):79-87. doi:10.1016/j.vph.2014.09.001 25446168

[49] Araujo RL, Gönen M, Herman P. Chemotherapy for patients with colorectal liver metastases who underwent curative resection improves long-term outcomes: systematic review and meta-analysis. Ann Surg Oncol. 2015;22(9):3070-3078. doi:10.1245/s10434-014-4354-6 25586244

[50] Arnaud L, Mathian A, Ruffatti A, . Efficacy of aspirin for the primary prevention of thrombosis in patients with antiphospholipid antibodies: an international and collaborative meta-analysis. Autoimmun Rev. 2014;13(3):281-291. doi:10.1016/j.autrev.2013.10.014 24189281

[51] Austin N, Cleminson J, Darlow BA, McGuire W. Prophylactic oral/topical non-absorbed antifungal agents to prevent invasive fungal infection in very low birth weight infants. Cochrane Database Syst Rev. 2015;2015(10):CD003478. doi:10.1002/14651858.CD003478.pub5 26497202 PMC7154334

[52] Ayoub K, Nairooz R, Almomani A, Marji M, Paydak H, Maskoun W. Perioperative heparin bridging in atrial fibrillation patients requiring temporary interruption of anticoagulation: evidence from meta-analysis. J Stroke Cerebrovasc Dis. 2016;25(9):2215-2221. doi:10.1016/j.jstrokecerebrovasdis.2016.04.006 27289185

[53] Bai Y, Miller T, Tan M, Law LS, Gan TJ. Lidocaine patch for acute pain management: a meta-analysis of prospective controlled trials. Curr Med Res Opin. 2015;31(3):575-581. doi:10.1185/03007995.2014.973484 25290665

[54] Bakhsheshian J, Dahdaleh NS, Lam SK, Savage JW, Smith ZA. The use of vancomycin powder in modern spine surgery: systematic review and meta-analysis of the clinical evidence. World Neurosurg. 2015;83(5):816-823. doi:10.1016/j.wneu.2014.12.033 25535069

[55] Baldinger R, Katzberg HD, Weber M. Treatment for cramps in amyotrophic lateral sclerosis/motor neuron disease. Cochrane Database Syst Rev. 2012;(4):CD004157. doi:10.1002/14651858.CD004157.pub2 22513921 PMC12107517

[56] Ballinger AE, Palmer SC, Wiggins KJ, . Treatment for peritoneal dialysis-associated peritonitis. Cochrane Database Syst Rev. 2014;2014(4):CD005284. 24771351 10.1002/14651858.CD005284.pub3 PMC11231986

[57] Bang CS, Baik GH, Shin IS, . Effect of intragastric injection of botulinum toxin A for the treatment of obesity: a meta-analysis and meta-regression. Gastrointest Endosc. 2015;81(5):1141-9.e1, 7. doi:10.1016/j.gie.2014.12.025 25765772

[58] Barkat M, Hajibandeh S, Hajibandeh S, Torella F, Antoniou GA. Systematic review and meta-analysis of dual versus single antiplatelet therapy in carotid interventions. Eur J Vasc Endovasc Surg. 2017;53(1):53-67. doi:10.1016/j.ejvs.2016.10.011 27894896

[59] Bellemain-Appaix A, Kerneis M, O’Connor SA, ; ACTION Study Group. Reappraisal of thienopyridine pretreatment in patients with non-ST elevation acute coronary syndrome: a systematic review and meta-analysis. BMJ. 2014;349:g6269. doi:10.1136/bmj.g6269 25954988 PMC4208629

[60] Benjo A, Cardoso RN, Collins T, . Vascular brachytherapy versus drug-eluting stents in the treatment of in-stent restenosis: a meta-analysis of long-term outcomes. Catheter Cardiovasc Interv. 2016;87(2):200-208. doi:10.1002/ccd.25998 25963829

[61] Bhangu A, Singh P, Fitzgerald JE, Slesser A, Tekkis P. Postoperative nonsteroidal anti-inflammatory drugs and risk of anastomotic leak: meta-analysis of clinical and experimental studies. World J Surg. 2014;38(9):2247-2257. doi:10.1007/s00268-014-2531-1 24682313

[62] Bloom JE, Rischin A, Johnston RV, Buchbinder R. Image-guided versus blind glucocorticoid injection for shoulder pain. Cochrane Database Syst Rev. 2012;(8):CD009147. doi:10.1002/14651858.CD009147.pub2 22895984

[63] Bonet M, Ota E, Chibueze CE, Oladapo OT. Routine antibiotic prophylaxis after normal vaginal birth for reducing maternal infectious morbidity. Cochrane Database Syst Rev. 2017;11(11):CD012137. doi:10.1002/14651858.CD012137.pub2 29190037 PMC6486135

[64] Bosanquet DC, Glasbey JC, Stimpson A, Williams IM, Twine CP. Systematic review and meta-analysis of the efficacy of perineural local anaesthetic catheters after major lower limb amputation. Eur J Vasc Endovasc Surg. 2015;50(2):241-249. doi:10.1016/j.ejvs.2015.04.030 26067167

[65] Bossard M, Mehta SR, Welsh RC, Bainey KR. Utility of unfractionated heparin in transradial cardiac catheterization: a systematic review and meta-analysis. Can J Cardiol. 2017;33(10):1245-1253. doi:10.1016/j.cjca.2017.06.003 28866078

[66] Boyle RJ, Elremeli M, Hockenhull J, . Venom immunotherapy for preventing allergic reactions to insect stings. Cochrane Database Syst Rev. 2012;10(10):CD008838. doi:10.1002/14651858.CD008838.pub2 23076950 PMC8734599

[67] Branger P, Parienti JJ, Sormani MP, Defer G. The effect of disease-modifying drugs on brain atrophy in relapsing-remitting multiple sclerosis: a meta-analysis. PLoS One. 2016;11(3):e0149685. doi:10.1371/journal.pone.0149685 26983008 PMC4794160

[68] Brennan M, Young G, Devane D. Topical preparations for preventing stretch marks in pregnancy. Cochrane Database Syst Rev. 2012;11(11):CD000066. doi:10.1002/14651858.CD000066.pub2 23152199 PMC10001689

[69] Brito NC, Rabello A, Cota GF. Efficacy of pentavalent antimoniate intralesional infiltration therapy for cutaneous leishmaniasis: a systematic review. PLoS One. 2017;12(9):e0184777. doi:10.1371/journal.pone.0184777 28926630 PMC5604971

[70] Brogly SB, Saia KA, Walley AY, Du HM, Sebastiani P. Prenatal buprenorphine versus methadone exposure and neonatal outcomes: systematic review and meta-analysis. Am J Epidemiol. 2014;180(7):673-686. doi:10.1093/aje/kwu190 25150272

[71] Brustia R, Granger B, Scatton O. An update on topical haemostatic agents in liver surgery: systematic review and meta analysis. J Hepatobiliary Pancreat Sci. 2016;23(10):609-621. doi:10.1002/jhbp.389 27580747

[72] Budden A, Chen LJ, Henry A. High-dose versus low-dose oxytocin infusion regimens for induction of labour at term. Cochrane Database Syst Rev. 2014;2014(10):CD009701. doi:10.1002/14651858.CD009701.pub2 25300173 PMC8932234

[73] Caldwell PH, Sureshkumar P, Wong WC. Tricyclic and related drugs for nocturnal enuresis in children. Cochrane Database Syst Rev. 2016;2016(1):CD002117. doi:10.1002/14651858.CD002117.pub2 26789925 PMC8741207

[74] Campbell D, Mudge DW, Craig JC, Johnson DW, Tong A, Strippoli GF. Antimicrobial agents for preventing peritonitis in peritoneal dialysis patients. Cochrane Database Syst Rev. 2017;4(4):CD004679. doi:10.1002/14651858.CD004679.pub3 28390069 PMC6478113

[75] Carneiro A, Sasse AD, Wagner AA, . Cardiovascular events associated with androgen deprivation therapy in patients with prostate cancer: a systematic review and meta-analysis. World J Urol. 2015;33(9):1281-1289. doi:10.1007/s00345-014-1439-6 25387877

[76] Chai-Adisaksopha C, Hillis C, Siegal DM, . Prothrombin complex concentrates versus fresh frozen plasma for warfarin reversal: a systematic review and meta-analysis. Thromb Haemost. 2016;116(5):879-890. doi:10.1160/TH16-04-0266 27488143

[77] Chalhoub JM, Rimmani HH, Gumaste VV, Sharara AI. Systematic review and meta-analysis: adalimumab monotherapy versus combination therapy with immunomodulators for induction and maintenance of remission and response in patients with Crohn’s disease. Inflamm Bowel Dis. 2017;23(8):1316-1327. doi:10.1097/MIB.0000000000001203 28719541

[78] Chao M, Zhang Y, Liang C. Impact of preoperative hormonal stimulation on postoperative complication rates after hypospadias repair: a meta-analysis. Minerva Urol Nefrol. 2017;69(3):253-261. doi:10.23736/S0393-2249.16.02634-5 27163504

[79] Chen CF, Chen B, Zhu J, Xu YZ. Antithrombotic therapy after percutaneous coronary intervention in patients requiring oral anticoagulant treatment: a meta-analysis. Herz. 2015;40(8):1070-1083. doi:10.1007/s00059-015-4325-0 26135462

[80] Chen J, Han X, An M, . Immunological and virological benefits resulted from short-course treatment during primary HIV infection: a meta-analysis. PLoS One. 2013;8(12):e82461. doi:10.1371/journal.pone.0082461 24324793 PMC3855754

[81] Chen X, Chen Y, Cai X, . Efficacy and safety of bevacizumab in elderly patients with advanced colorectal cancer: a meta-analysis. J Cancer Res Ther. 2017;13(5):869-877. doi:10.4103/jcrt.JCRT_833_17 29237919

[82] Chen Z, Liang JQ, Wang JH, Feng SS, Zhang GY. Moxifloxacin plus standard first-line therapy in the treatment of pulmonary tuberculosis: a meta-analysis. Tuberculosis (Edinb). 2015;95(4):490-496. doi:10.1016/j.tube.2015.03.014 25964137

[83] Cheng SP, Liu TP, Yang PS, Lee KS, Liu CL. Effect of perioperative dexamethasone on subjective voice quality after thyroidectomy: a meta-analysis and systematic review. Langenbecks Arch Surg. 2015;400(8):929-936. doi:10.1007/s00423-015-1354-3 26545607

[84] Chowdhury A, Fernandes B, Melhuish TM, White LD. Antiarrhythmics in cardiac arrest: a systematic review and meta-analysis. Heart Lung Circ. 2018;27(3):280-290. doi:10.1016/j.hlc.2017.07.004 28988724

[85] Chrcanovic BR, Albrektsson T, Wennerberg A. Prophylactic antibiotic regimen and dental implant failure: a meta-analysis. J Oral Rehabil. 2014;41(12):941-956. doi:10.1111/joor.12211 25040894

[86] Clifton P. Do dipeptidyl peptidase IV (DPP-IV) inhibitors cause heart failure? Clin Ther. 2014;36(12):2072-2079. doi:10.1016/j.clinthera.2014.10.009 25453730

[87] Coppola A, Windyga J, Tufano A, Yeung C, Di Minno MN. Treatment for preventing bleeding in people with haemophilia or other congenital bleeding disorders undergoing surgery. Cochrane Database Syst Rev. 2015;2015(2):CD009961. doi:10.1002/14651858.CD009961.pub2 25922858 PMC11245682

[88] Costi D, Cyna AM, Ahmed S, . Effects of sevoflurane versus other general anaesthesia on emergence agitation in children. Cochrane Database Syst Rev. 2014;2014(9):CD007084. doi:10.1002/14651858.CD007084.pub2 25212274 PMC10898224

[89] Coussement J, Scemla A, Abramowicz D, Nagler EV, Webster AC. Antibiotics for asymptomatic bacteriuria in kidney transplant recipients. Cochrane Database Syst Rev. 2018;2(2):CD011357. doi:10.1002/14651858.CD011357.pub2 29390169 PMC6491324

[90] Critchley JA, Orton LC, Pearson F. Adjunctive steroid therapy for managing pulmonary tuberculosis. Cochrane Database Syst Rev. 2014;2014(11):CD011370. doi:10.1002/14651858.CD011370 25387839 PMC6532561

[91] Cui XJ, He Q, Zhang JM, Fan HJ, Wen ZF, Qin YR. High-dose aspirin consumption contributes to decreased risk for pancreatic cancer in a systematic review and meta-analysis. Pancreas. 2014;43(1):135-140. doi:10.1097/MPA.0b013e3182a8d41f 24263109

[92] Dahal K, Kunwar S, Rijal J, . The effects of aldosterone antagonists in patients with resistant hypertension: a meta-analysis of randomized and nonrandomized studies. Am J Hypertens. 2015;28(11):1376-1385. doi:10.1093/ajh/hpv031 25801902

[93] David JA, Sankarapandian V, Christopher PR, Chatterjee A, Macaden AS. Injected corticosteroids for treating plantar heel pain in adults. Cochrane Database Syst Rev. 2017;6(6):CD009348. doi:10.1002/14651858.CD009348.pub2 28602048 PMC6481652

[94] de Frutos F, Gea A, Hernandez-Estefania R, Rabago G. Prophylactic treatment with coenzyme Q10 in patients undergoing cardiac surgery: could an antioxidant reduce complications? A systematic review and meta-analysis. Interact Cardiovasc Thorac Surg. 2015;20(2):254-259. doi:10.1093/icvts/ivu334 25344142

[95] Desiderio J, Chao J, Melstrom L, . The 30-year experience-a meta-analysis of randomised and high-quality non-randomised studies of hyperthermic intraperitoneal chemotherapy in the treatment of gastric cancer. Eur J Cancer. 2017;79:1-14. doi:10.1016/j.ejca.2017.03.030 28456089 PMC5568419

[96] Di X, Bai N, Zhang X, . A meta-analysis of metronidazole and vancomycin for the treatment of Clostridium difficile infection, stratified by disease severity. Braz J Infect Dis. 2015;19(4):339-349. doi:10.1016/j.bjid.2015.03.006 26001980 PMC9427463

[97] Dong SQ, Singh TP, Wei X, Yao H, Wang HL. Review: a Japanese population-based meta-analysis of vonoprazan versus PPI for Helicobacter pylori eradication therapy: is superiority an illusion? Helicobacter. 2017;22(6). doi:10.1111/hel.12438 28884937

[98] Edmonds ML, Milan SJ, Camargo CA Jr, Pollack CV, Rowe BH. Early use of inhaled corticosteroids in the emergency department treatment of acute asthma. Cochrane Database Syst Rev. 2012;12(12):CD002308. doi:10.1002/14651858.CD002308.pub2 23235589 PMC6513646

[99] El Sayed I, Liu Q, Wee I, Hine P. Antibiotics for treating scrub typhus. Cochrane Database Syst Rev. 2018;9(9):CD002150. 30246875 10.1002/14651858.CD002150.pub2 PMC6485465

[100] Elgendy AY, Mahtta D, Barakat AF, . Meta-analysis of safety and efficacy of uninterrupted non-vitamin K antagonist oral anticoagulants versus vitamin K antagonists for catheter ablation of atrial fibrillation. Am J Cardiol. 2017;120(10):1830-1836. doi:10.1016/j.amjcard.2017.07.096 28882334

[101] Engelen ET, Schutgens RE, Mauser-Bunschoten EP, van Es RJ, van Galen KP. Antifibrinolytic therapy for preventing oral bleeding in people on anticoagulants undergoing minor oral surgery or dental extractions. Cochrane Database Syst Rev. 2018;7(7):CD012293. doi:10.1002/14651858.CD012293.pub2 29963686 PMC6513563

[102] Engelman E, Maeyens C. Effect of preoperative single-dose corticosteroid administration on postoperative morbidity following esophagectomy. J Gastrointest Surg. 2010;14(5):788-804. doi:10.1007/s11605-010-1168-0 20229072

[103] Estcourt LJ, Stanworth S, Doree C, . Granulocyte transfusions for preventing infections in people with neutropenia or neutrophil dysfunction. Cochrane Database Syst Rev. 2015;2015(6):CD005341. doi:10.1002/14651858.CD005341.pub3 26118415 PMC4538863

[104] Facciorusso A, Roy S, Livadas S, . Nonselective beta-blockers do not affect survival in cirrhotic patients with ascites. Dig Dis Sci. 2018;63(7):1737-1746. doi:10.1007/s10620-018-5092-6 29725793

[105] Falagas ME, Karageorgopoulos DE, Tansarli GS. continuous versus conventional infusion of amphotericin B deoxycholate: a meta-analysis. PLoS One. 2013;8(10):e77075. doi:10.1371/journal.pone.0077075 24204739 PMC3804519

[106] Feng L, Lin XF, Wan ZH, Hu D, Du YK. Efficacy of metformin on pregnancy complications in women with polycystic ovary syndrome: a meta-analysis. Gynecol Endocrinol. 2015;31(11):833-839. doi:10.3109/09513590.2015.1041906 26440203

[107] Ferrer P, Amelio J, Ballarín E, ; PROTECT Work Package 2. Systematic review and meta-analysis: macrolides- and amoxicillin/clavulanate-induced acute liver injury. Basic Clin Pharmacol Toxicol. 2016;119(1):3-9. doi:10.1111/bcpt.12550 26707367

[108] Filippini G, Del Giovane C, Clerico M, . Treatment with disease-modifying drugs for people with a first clinical attack suggestive of multiple sclerosis. Cochrane Database Syst Rev. 2017;4(4):CD012200. doi:10.1002/14651858.CD012200.pub2 28440858 PMC6478290

[109] Fukuta H, Goto T, Wakami K, Ohte N. The effect of beta-blockers on mortality in heart failure with preserved ejection fraction: a meta-analysis of observational cohort and randomized controlled studies. Int J Cardiol. 2017;228:4-10. doi:10.1016/j.ijcard.2016.11.239 27863360

[110] Fung M, Kim J, Marty FM, Schwarzinger M, Koo S. Meta-analysis and cost comparison of empirical versus pre-emptive antifungal strategies in hematologic malignancy patients with high-risk febrile neutropenia. PLoS One. 2015;10(11):e0140930. doi:10.1371/journal.pone.0140930 26554923 PMC4640557

[111] Furtado R, Crawford M, Sandroussi C. Systematic review and meta-analysis of adjuvant i(131) lipiodol after excision of hepatocellular carcinoma. Ann Surg Oncol. 2014;21(8):2700-2707. doi:10.1245/s10434-014-3511-2 24743904

[112] Galappaththy GN, Tharyan P, Kirubakaran R. Primaquine for preventing relapse in people with Plasmodium vivax malaria treated with chloroquine. Cochrane Database Syst Rev. 2013;2013(10):CD004389. 24163057 10.1002/14651858.CD004389.pub3 PMC6532739

[113] Gandhi S, Schwalm JD, Velianou JL, Natarajan MK, Farkouh ME. Comparison of dual-antiplatelet therapy to mono-antiplatelet therapy after transcatheter aortic valve implantation: systematic review and meta-analysis. Can J Cardiol. 2015;31(6):775-784. doi:10.1016/j.cjca.2015.01.014 25913473

[114] Gausden EB, Qudsi R, Boone MD, OʼGara B, Ruzbarsky JJ, Lorich DG. Tranexamic acid in orthopaedic trauma surgery: a meta-analysis. J Orthop Trauma. 2017;31(10):513-519. doi:10.1097/BOT.0000000000000913 28938281 PMC6827340

[115] Gharaibeh A, Savage HI, Scherer RW, Goldberg MF, Lindsley K. Medical interventions for traumatic hyphema. Cochrane Database Syst Rev. 2013;12(12):CD005431. 24302299 10.1002/14651858.CD005431.pub3 PMC4268787

[116] Gillespie WJ, Walenkamp GH. Antibiotic prophylaxis for surgery for proximal femoral and other closed long bone fractures. Cochrane Database Syst Rev. 2010;2010(3):CD000244. doi:10.1002/14651858.CD000244.pub2 20238310 PMC7043359

[117] Gong Q, Janowski M, Luo M, . Efficacy and adverse effects of atropine in childhood myopia: a meta-analysis. JAMA Ophthalmol. 2017;135(6):624-630. doi:10.1001/jamaophthalmol.2017.1091 28494063 PMC5710262

[118] González R, Pons-Duran C, Piqueras M, Aponte JJ, Ter Kuile FO, Menéndez C. Mefloquine for preventing malaria in pregnant women. Cochrane Database Syst Rev. 2018;3(3):CD011444. 29561063 10.1002/14651858.CD011444.pub2 PMC5875065

[119] Grabein B, Graninger W, Rodríguez Baño J, Dinh A, Liesenfeld DB. Intravenous fosfomycin-back to the future: systematic review and meta-analysis of the clinical literature. Clin Microbiol Infect. 2017;23(6):363-372. doi:10.1016/j.cmi.2016.12.005 27956267

[120] Graves PM, Choi L, Gelband H, Garner P. Primaquine or other 8-aminoquinolines for reducing Plasmodium falciparum transmission. Cochrane Database Syst Rev. 2018;2(2):CD008152. doi:10.1002/14651858.CD008152.pub5 29393511 PMC5815493

[121] Graves PM, Deeks JJ, Demicheli V, Jefferson T. Vaccines for preventing cholera: killed whole cell or other subunit vaccines (injected). Cochrane Database Syst Rev. 2010;2010(8):CD000974. doi:10.1002/14651858.CD000974.pub2 20687062 PMC6532721

[122] Gray RT, Coleman HG, Hughes C, Murray LJ, Cardwell CR. Statin use and survival in colorectal cancer: Results from a population-based cohort study and an updated systematic review and meta-analysis. Cancer Epidemiol. 2016;45:71-81. doi:10.1016/j.canep.2016.10.004 27750068

[123] Guerra F, Romandini A, Barbarossa A, Belardinelli L, Capucci A. Ranolazine for rhythm control in atrial fibrillation: a systematic review and meta-analysis. Int J Cardiol. 2017;227:284-291. doi:10.1016/j.ijcard.2016.11.103 27839812

[124] Gunter BR, Butler KA, Wallace RL, Smith SM, Harirforoosh S. Non-steroidal anti-inflammatory drug-induced cardiovascular adverse events: a meta-analysis. J Clin Pharm Ther. 2017;42(1):27-38. doi:10.1111/jcpt.12484 28019014

[125] Haas DM, Morgan AM, Deans SJ, Schubert FP. Ethanol for preventing preterm birth in threatened preterm labor. Cochrane Database Syst Rev. 2015;2015(11):CD011445. doi:10.1002/14651858.CD011445.pub2 26544539 PMC8944412

[126] Haas DM, Morgan S, Contreras K, Enders S. Vaginal preparation with antiseptic solution before cesarean section for preventing postoperative infections. Cochrane Database Syst Rev. 2018;7(7):CD007892. doi:10.1002/14651858.CD007892.pub6 30016540 PMC6513039

[127] Han X, Yang X, Huang B, Yuan L, Cao Y. Low-dose versus high-dose heparin locks for hemodialysis catheters: a systematic review and meta-analysis. Clin Nephrol. 2016;86(7):1-8. doi:10.5414/CN108701 27191662

[128] Han Y, Zeng A, Liao H, Liu Y, Chen Y, Ding H. The efficacy and safety comparison between tenofovir and entecavir in treatment of chronic hepatitis B and HBV related cirrhosis: a systematic review and Meta-analysis. Int Immunopharmacol. 2017;42:168-175. doi:10.1016/j.intimp.2016.11.022 27915131

[129] Hannah J, Casian A, D’Cruz D. Tacrolimus use in lupus nephritis: a systematic review and meta-analysis. Autoimmun Rev. 2016;15(1):93-101. doi:10.1016/j.autrev.2015.09.006 26427983

[130] Hao JJ, Chen H, Zhou JX. Continuous versus intermittent infusion of vancomycin in adult patients: a systematic review and meta-analysis. Int J Antimicrob Agents. 2016;47(1):28-35. doi:10.1016/j.ijantimicag.2015.10.019 26655032

[131] Harnoss JC, Zelienka I, Probst P, . Antibiotics versus surgical therapy for uncomplicated appendicitis: systematic review and meta-analysis of controlled trials (PROSPERO 2015: CRD42015016882). Ann Surg. 2017;265(5):889-900. doi:10.1097/SLA.0000000000002039 27759621

[132] Haroon NN, Sriganthan J, Al Ghanim N, Inman RD, Cheung AM. Effect of TNF-alpha inhibitor treatment on bone mineral density in patients with ankylosing spondylitis: a systematic review and meta-analysis. Semin Arthritis Rheum. 2014;44(2):155-161. doi:10.1016/j.semarthrit.2014.05.008 24909809

[133] He MM, Wu WJ, Wang F, . S-1-based chemotherapy versus capecitabine-based chemotherapy as first-line treatment for advanced gastric carcinoma: a meta-analysis. PLoS One. 2013;8(12):e82798. doi:10.1371/journal.pone.0082798 24349363 PMC3861463

[134] He Y, Chan EW, Leung WK, Anand S, Wong IC. Systematic review with meta-analysis: the association between the use of calcium channel blockers and gastrointestinal bleeding. Aliment Pharmacol Ther. 2015;41(12):1246-1255. doi:10.1111/apt.13211 25898902

[135] Heal CF, Banks JL, Lepper P, Kontopantelis E, van Driel ML. Meta-analysis of randomized and quasi-randomized clinical trials of topical antibiotics after primary closure for the prevention of surgical-site infection. Br J Surg. 2017;104(9):1123-1130. doi:10.1002/bjs.10588 28656693

[136] Henderson-Smart DJ, De Paoli AG. Methylxanthine treatment for apnoea in preterm infants. Cochrane Database Syst Rev. 2010;(12):CD000140. doi:10.1002/14651858.CD000140.pub2 21154343 PMC11751766

[137] Henssler J, Bschor T, Baethge C. Combining antidepressants in acute treatment of depression: a meta-analysis of 38 studies including 4511 patients. Can J Psychiatry. 2016;61(1):29-43. doi:10.1177/0706743715620411 27582451 PMC4756602

[138] Hernandez AV, Thota P, Pellegrino D, . A systematic review and meta-analysis of the relative efficacy and safety of treatment regimens for HIV-associated cerebral toxoplasmosis: is trimethoprim-sulfamethoxazole a real option? HIV Med. 2017;18(2):115-124. doi:10.1111/hiv.12402 27353303

[139] Hodson EM, Ladhani M, Webster AC, Strippoli GF, Craig JC. Antiviral medications for preventing cytomegalovirus disease in solid organ transplant recipients. Cochrane Database Syst Rev. 2013;(2):CD003774. doi:10.1002/14651858.CD003774.pub4 23450543

[140] Hong D, Yang Z, Han S, Liang X, Ma K, Zhang X. Interleukin 1 inhibition with anakinra in adult-onset Still disease: a meta-analysis of its efficacy and safety. Drug Des Devel Ther. 2014;8:2345-2357. 25473268 10.2147/DDDT.S73428 PMC4251663

[141] Horbach SER, Rigter IM, Smitt JHS, Reekers JA, Spuls PI, van der Horst CMAM. Intralesional bleomycin injections for vascular malformations: a systematic review and meta-analysis. Plast Reconstr Surg. 2016;137(1):244-256. doi:10.1097/PRS.0000000000001924 26710030

[142] Horita N, Otsuka T, Haranaga S, . Beta-lactam plus macrolides or beta-lactam alone for community-acquired pneumonia: a systematic review and meta-analysis. Respirology. 2016;21(7):1193-1200. doi:10.1111/resp.12835 27338144

[143] Hu H, Xie Y, Yang G, Jian C, Deng Y. Nonsteroidal anti-inflammatory drug use and the risk of melanoma: a meta-analysis. Eur J Cancer Prev. 2014;23(1):62-68. doi:10.1097/CEJ.0b013e328360f479 23549150

[144] Hu J, Zhang Q, Ren X, Sun Z, Quan Q. Efficacy and safety of acetylcysteine in “non-acetaminophen” acute liver failure: a meta-analysis of prospective clinical trials. Clin Res Hepatol Gastroenterol. 2015;39(5):594-599. doi:10.1016/j.clinre.2015.01.003 25732608

[145] Hu MD, Jia LH, Liu HB, Zhang KH, Guo GH. Sorafenib in combination with transarterial chemoembolization for hepatocellular carcinoma: a meta-analysis. Eur Rev Med Pharmacol Sci. 2016;20(1):64-74. 26813455

[146] Huang L, Yin Y, Yang L, Wang C, Li Y, Zhou Z. Comparison of antibiotic therapy and appendectomy for acute uncomplicated appendicitis in children: a meta-analysis. JAMA Pediatr. 2017;171(5):426-434. doi:10.1001/jamapediatrics.2017.0057 28346589 PMC5470362

[147] Huang QY, Rong MH, Lan AH, . The impact of atosiban on pregnancy outcomes in women undergoing in vitro fertilization-embryo transfer: a meta-analysis. PLoS One. 2017;12(4):e0175501. doi:10.1371/journal.pone.0175501 28422984 PMC5396917

[148] Huang XC, Hu XH, Wang XR, Zhou CX, Wang GY. Efficacy and safety of therapeutic anticoagulation for the treatment of isolated calf muscle vein thrombosis - a systematic review and meta-analysis. Vasa. 2016;45(6):478-485. doi:10.1024/0301-1526/a000569 27598049

[149] Huang Y, He Q, Yang M, Zhan L. Antiarrhythmia drugs for cardiac arrest: a systemic review and meta-analysis. Crit Care. 2013;17(4):R173. doi:10.1186/cc12852 23938138 PMC4056084

[150] Hughes RA, Brassington R, Gunn AA, van Doorn PA. Corticosteroids for Guillain-Barré syndrome. Cochrane Database Syst Rev. 2016;10(10):CD001446. 27775812 10.1002/14651858.CD001446.pub5 PMC6464149

[151] Hunt R, Hey E. Ethamsylate for the prevention of morbidity and mortality in preterm or very low birth weight infants. Cochrane Database Syst Rev. 2010;(1):CD004343. doi:10.1002/14651858.CD004343.pub2 20091562 PMC12744937

[152] Hyun MH, Lee YS, Kim JH, . Systematic review with meta-analysis: the efficacy and safety of tenofovir to prevent mother-to-child transmission of hepatitis B virus. Aliment Pharmacol Ther. 2017;45(12):1493-1505. doi:10.1111/apt.14068 28436552

[153] Jain P, Sharma S, Dua T, Barbui C, Das RR, Aneja S. Efficacy and safety of anti-epileptic drugs in patients with active convulsive seizures when no IV access is available: systematic review and meta-analysis. Epilepsy Res. 2016;122:47-55. doi:10.1016/j.eplepsyres.2016.02.006 26922313

[154] Ji S, Wei Y, Chen J, Tang S. Clinical efficacy of anti-VEGF medications for central serous chorioretinopathy: a meta-analysis. Int J Clin Pharm. 2017;39(3):514-521. doi:10.1007/s11096-017-0460-4 28386700

[155] Jian-Yu E, Graber JM, Lu SE, Lin Y, Lu-Yao G, Tan XL. Effect of metformin and statin use on survival in pancreatic cancer patients: a systematic literature review and meta-analysis. Curr Med Chem. 2018;25(22):2595-2607. doi:10.2174/0929867324666170412145232 28403788 PMC5638687

[156] Jiang R, Wang L, Zhu CT, . Comparative effectiveness of sildenafil for pulmonary hypertension due to left heart disease with HFrEF. Hypertens Res. 2015;38(12):829-839. doi:10.1038/hr.2015.73 26202179

[157] Jiang X, Ma XL, Ma JX. Efficiency and safety of intravenous tranexamic acid in simultaneous bilateral total knee arthroplasty: a systematic review and Meta-analysis. Orthop Surg. 2016;8(3):285-293. doi:10.1111/os.12256 27627710 PMC6584435

[158] Johnston ANB, Park J, Doi SA, . Effect of immediate administration of antibiotics in patients with sepsis in tertiary care: a systematic review and meta-analysis. Clin Ther. 2017;39(1):190-202.e6. doi:10.1016/j.clinthera.2016.12.003 28062114

[159] Kabra SK, Lodha R. Antibiotics for preventing complications in children with measles. Cochrane Database Syst Rev. 2013;2013(8):CD001477. doi:10.1002/14651858.CD001477.pub4 23943263 PMC7055587

[160] Kalil AC, Freifeld AG, Lyden ER, Stoner JA. Valganciclovir for cytomegalovirus prevention in solid organ transplant patients: an evidence-based reassessment of safety and efficacy. PLoS One. 2009;4(5):e5512. doi:10.1371/journal.pone.0005512 19436751 PMC2677673

[161] Kamal F, Khan MA, Khan Z, . Rifaximin for the prevention of spontaneous bacterial peritonitis and hepatorenal syndrome in cirrhosis: a systematic review and meta-analysis. Eur J Gastroenterol Hepatol. 2017;29(10):1109-1117. doi:10.1097/MEG.0000000000000940 28763340

[162] Kamal S, Khan MA, Seth A, . Beneficial effects of statins on the rates of hepatic fibrosis, hepatic decompensation, and mortality in chronic liver disease: a systematic review and meta-analysis. Am J Gastroenterol. 2017;112(10):1495-1505. doi:10.1038/ajg.2017.170 28585556

[163] Kanbay M, Siriopol D, Nistor I, . Effects of allopurinol on endothelial dysfunction: a meta-analysis. Am J Nephrol. 2014;39(4):348-356. doi:10.1159/000360609 24751886

[164] Kaplan YC, Ozsarfati J, Nickel C, Koren G. Reproductive outcomes following hydroxychloroquine use for autoimmune diseases: a systematic review and meta-analysis. Br J Clin Pharmacol. 2016;81(5):835-848. doi:10.1111/bcp.12872 26700396 PMC4834589

[165] Kenyon S, Tokumasu H, Dowswell T, Pledge D, Mori R. High-dose versus low-dose oxytocin for augmentation of delayed labour. Cochrane Database Syst Rev. 2013;2013(7):CD007201. doi:10.1002/14651858.CD007201.pub3 23853046 PMC10575623

[166] Kessel L, Flesner P, Andresen J, Erngaard D, Tendal B, Hjortdal J. Antibiotic prevention of postcataract endophthalmitis: a systematic review and meta-analysis. Acta Ophthalmol. 2015;93(4):303-317. doi:10.1111/aos.12684 25779209 PMC6680152

[167] Khan M, Boyce A, Prieto-Merino D, Svensson Å, Wedgeworth E, Flohr C. The Role of Topical Timolol in the Treatment of Infantile Hemangiomas: A Systematic Review and Meta-analysis. Acta Derm Venereol. 2017;97(10):1167-1171. doi:10.2340/00015555-2681 28421234

[168] Khan MS, Fonarow GC, Khan H, . Renin-angiotensin blockade in heart failure with preserved ejection fraction: a systematic review and meta-analysis. ESC Heart Fail. 2017;4(4):402-408. doi:10.1002/ehf2.12204 28869332 PMC5695183

[169] Khan NR, VanLandingham MA, Fierst TM, . Should levetiracetam or phenytoin be used for posttraumatic seizure prophylaxis? a systematic review of the literature and meta-analysis. Neurosurgery. 2016;79(6):775-782. doi:10.1227/NEU.0000000000001445 27749510

[170] Khoshbin A, Leroux T, Wasserstein D, . The efficacy of platelet-rich plasma in the treatment of symptomatic knee osteoarthritis: a systematic review with quantitative synthesis. Arthroscopy. 2013;29(12):2037-2048. doi:10.1016/j.arthro.2013.09.006 24286802

[171] Kim JS, Kwon SH. Mupirocin in the treatment of staphylococcal infections in chronic rhinosinusitis: a meta-analysis. PLoS One. 2016;11(12):e0167369. doi:10.1371/journal.pone.0167369 27907108 PMC5132234

[172] Kirkland SW, Vandenberghe C, Voaklander B, Nikel T, Campbell S, Rowe BH. Combined inhaled beta-agonist and anticholinergic agents for emergency management in adults with asthma. Cochrane Database Syst Rev. 2017;1(1):CD001284. doi:10.1002/14651858.CD001284.pub2 28076656 PMC6465060

[173] Kirsch JM, Bedi A, Horner N, . Tranexamic acid in shoulder arthroplasty: a systematic review and meta-analysis. JBJS Rev. 2017;5(9):e3. doi:10.2106/JBJS.RVW.17.00021 28902659

[174] Kitsios GD, Dahabreh IJ, Callahan S, Paulus JK, Campagna AC, Dargin JM. Can we trust observational studies using propensity scores in the critical care literature? a systematic comparison with randomized clinical trials. Crit Care Med. 2015;43(9):1870-1879. doi:10.1097/CCM.0000000000001135 26086943

[175] Klimo P Jr, Van Poppel M, Thompson CJ, Baird LC, Duhaime AC, Flannery AM; Pediatric Hydrocephalus Systematic Review and Evidence-Based Guidelines Task Force. Pediatric hydrocephalus: systematic literature review and evidence-based guidelines. Part 6: preoperative antibiotics for shunt surgery in children with hydrocephalus: a systematic review and meta-analysis. J Neurosurg Pediatr. 2014;14(suppl 1):44-52. doi:10.3171/2014.7.PEDS14326 25988782

[176] Kovacs SD, van Eijk AM, Sevene E, . The safety of artemisinin derivatives for the treatment of malaria in the 2nd or 3rd trimester of pregnancy: a systematic review and meta-analysis. PLoS One. 2016;11(11):e0164963. doi:10.1371/journal.pone.0164963 27824884 PMC5100961

[177] Kowalewski M, Suwalski P, Raffa GM, . Meta-analysis of uninterrupted as compared to interrupted oral anticoagulation with or without bridging in patients undergoing coronary angiography with or without percutaneous coronary intervention. Int J Cardiol. 2016;223:186-194. doi:10.1016/j.ijcard.2016.08.089 27541652

[178] Krajewski ML, Raghunathan K, Paluszkiewicz SM, Schermer CR, Shaw AD. Meta-analysis of high- versus low-chloride content in perioperative and critical care fluid resuscitation. Br J Surg. 2015;102(1):24-36. doi:10.1002/bjs.9651 25357011 PMC4282059

[179] Kroon FP, van der Burg LR, Ramiro S, . Non-steroidal anti-inflammatory drugs (NSAIDs) for axial spondyloarthritis (ankylosing spondylitis and non-radiographic axial spondyloarthritis). Cochrane Database Syst Rev. 2015;2015(7):CD010952. doi:10.1002/14651858.CD010952.pub2 26186173 PMC8942090

[180] Kuang MJ, Du Y, Ma JX, He W, Fu L, Ma XL. The efficacy of liposomal bupivacaine using periarticular injection in total knee arthroplasty: a systematic review and meta-analysis. J Arthroplasty. 2017;32(4):1395-1402. doi:10.1016/j.arth.2016.12.025 28082044

[181] Kwok CS, Jeevanantham V, Dawn B, Loke YK. No consistent evidence of differential cardiovascular risk amongst proton-pump inhibitors when used with clopidogrel: meta-analysis. Int J Cardiol. 2013;167(3):965-974. doi:10.1016/j.ijcard.2012.03.085 22464478

[182] Lee J, Park JH, Jwa H, Kim YH. Comparison of efficacy of intravenous peramivir and oral oseltamivir for the treatment of influenza: systematic review and meta-analysis. Yonsei Med J. 2017;58(4):778-785. doi:10.3349/ymj.2017.58.4.778 28540991 PMC5447109

[183] Lee JH, Kim HJ, Kim YH. Is β-lactam plus macrolide more effective than β-lactam plus fluoroquinolone among patients with severe community-acquired pneumonia?: a systemic review and meta-analysis. J Korean Med Sci. 2017;32(1):77-84. doi:10.3346/jkms.2017.32.1.77 27914135 PMC5143302

[184] Leibovici-Weissman Y, Neuberger A, Bitterman R, Sinclair D, Salam MA, Paul M. Antimicrobial drugs for treating cholera. Cochrane Database Syst Rev. 2014;2014(6):CD008625. 24944120 10.1002/14651858.CD008625.pub2 PMC4468928

[185] Lemos LL, de Oliveira Costa J, Almeida AM, . Treatment of psoriatic arthritis with anti-TNF agents: a systematic review and meta-analysis of efficacy, effectiveness and safety. Rheumatol Int. 2014;34(10):1345-1360. doi:10.1007/s00296-014-3006-2 24728068

[186] Leone MA, Giussani G, Nolan SJ, Marson AG, Beghi E. Immediate antiepileptic drug treatment, versus placebo, deferred, or no treatment for first unprovoked seizure. Cochrane Database Syst Rev. 2016;2016(5):CD007144. doi:10.1002/14651858.CD007144.pub2 27150433 PMC6478062

[187] Lewis SR, Pritchard MW, Evans DJ, . Colloids versus crystalloids for fluid resuscitation in critically ill people. Cochrane Database Syst Rev. 2018;8(8):CD000567. doi:10.1002/14651858.CD000567.pub7 30073665 PMC6513027

[188] Li D, Chen C, Zhou Y, . Gemcitabine compared with gemcitabine and s-1 combination therapy in advanced pancreatic cancer: a systematic review and meta-analysis. Medicine (Baltimore). 2015;94(35):e1345. doi:10.1097/MD.0000000000001345 26334891 PMC4616522

[189] Li G, Holbrook A, Jin Y, . Comparison of treatment effect estimates of non-vitamin K antagonist oral anticoagulants versus warfarin between observational studies using propensity score methods and randomized controlled trials. Eur J Epidemiol. 2016;31(6):541-561. doi:10.1007/s10654-016-0178-y 27370013

[190] Li J, Li S, Yu H, Wang J, Xu C, Lu X. The efficacy and safety of first-line single-agent chemotherapy regimens in low-risk gestational trophoblastic neoplasia: a network meta-analysis. Gynecol Oncol. 2018;148(2):247-253. doi:10.1016/j.ygyno.2017.11.031 29203174

[191] Li L, Han Z, Yuan H, Zhang G, Jia Y, He C. Nonsteroidal anti-inflammatory drugs reduce the incidence of post-endoscopic retrograde cholangiopancreatography pancreatitis: a meta-analysis. J Hepatobiliary Pancreat Sci. 2017;24(9):520-529. doi:10.1002/jhbp.489 28681997

[192] Li X, Wang W, Zhang X. Meta-analysis of selective laser trabeculoplasty versus topical medication in the treatment of open-angle glaucoma. BMC Ophthalmol. 2015;15:107. doi:10.1186/s12886-015-0091-2 26286384 PMC4544808

[193] Liang JW, Zheng ZC, Yu T, Wang X, Zhang JJ. Is postoperative adjuvant chemoradiotherapy efficacious and safe for gastric cancer patients with D2 lymphadenectomy? a meta-analysis of the literature. Eur J Surg Oncol. 2014;40(12):1614-1621. doi:10.1016/j.ejso.2014.04.009 24813809

[194] Liang L, Cai Y, Li A, Ma C. The efficiency of intravenous acetaminophen for pain control following total knee and hip arthroplasty: A systematic review and meta-analysis. Medicine (Baltimore). 2017;96(46):e8586. doi:10.1097/MD.0000000000008586 29145272 PMC5704817

[195] Liet JM, Ducruet T, Gupta V, Cambonie G. Heliox inhalation therapy for bronchiolitis in infants. Cochrane Database Syst Rev. 2015;2015(9):CD006915. doi:10.1002/14651858.CD006915.pub3 26384333 PMC8504435

[196] Lim CL, Lee W, Liew YX, Tang SS, Chlebicki MP, Kwa AL. Role of antibiotic prophylaxis in necrotizing pancreatitis: a meta-analysis. J Gastrointest Surg. 2015;19(3):480-491. doi:10.1007/s11605-014-2662-6 25608671

[197] Lim JY, Deo SV, Rababa’h A, . Levosimendan reduces mortality in adults with left ventricular dysfunction undergoing cardiac surgery: a systematic review and meta-analysis. J Card Surg. 2015;30(7):547-554. doi:10.1111/jocs.12562 25989324

[198] Lin HS, Wan RH, Gao LH, Li JF, Shan RF, Shi J. Adjuvant chemotherapy after liver transplantation for hepatocellular carcinoma: a systematic review and a meta-analysis. Hepatobiliary Pancreat Dis Int. 2015;14(3):236-245. doi:10.1016/S1499-3872(15)60373-3 26063023

[199] Liu HY, Han Y, Chen XS, . Comparison of efficacy of treatments for early syphilis: a systematic review and network meta-analysis of randomized controlled trials and observational studies. PLoS One. 2017;12(6):e0180001. doi:10.1371/journal.pone.0180001 28658325 PMC5489196

[200] Liu J, Yang J, Chen Y, Cheng S, Xia C, Deng T. Is steroids therapy effective in treating phimosis? a meta-analysis. Int Urol Nephrol. 2016;48(3):335-342. doi:10.1007/s11255-015-1184-9 26725071

[201] Liu Q, Li W, Feng Y, Tao C. Efficacy and safety of polymyxins for the treatment of Acinectobacter baumannii infection: a systematic review and meta-analysis. PLoS One. 2014;9(6):e98091. doi:10.1371/journal.pone.0098091 24911658 PMC4049575

[202] Liu Y, Lu Y, Wang J, . Association between nonsteroidal anti-inflammatory drug use and brain tumour risk: a meta-analysis. Br J Clin Pharmacol. 2013;78(1):56-68. 24341448 10.1111/bcp.12311 PMC4168380

[203] Liu Z, Tao X, Chen Y, Fan Z, Li Y. Bed rest versus early ambulation with standard anticoagulation in the management of deep vein thrombosis: a meta-analysis. PLoS One. 2015;10(4):e0121388. doi:10.1371/journal.pone.0121388 25860350 PMC4393252

[204] Loomba RS, Nijhawan K, Aggarwal S, Arora RR. Increased return of spontaneous circulation at the expense of neurologic outcomes: is prehospital epinephrine for out-of-hospital cardiac arrest really worth it? J Crit Care. 2015;30(6):1376-1381. doi:10.1016/j.jcrc.2015.08.016 26428074

[205] Lu YP, Liang XJ, Xiao XM, . Telbivudine during the second and third trimester of pregnancy interrupts HBV intrauterine transmission: a systematic review and meta-analysis. Clin Lab. 2014;60(4):571-586. doi:10.7754/Clin.Lab.2013.130408 24779291

[206] Luni FK, Khan AR, Singh H, . Identification and ablation of dormant conduction in atrial fibrillation using adenosine. Am J Med Sci. 2018;355(1):27-36. doi:10.1016/j.amjms.2017.09.005 29289258

[207] Lussana F, Squizzato A, Permunian ET, Cattaneo M. A systematic review on the effect of aspirin in the prevention of post-operative arterial thrombosis in patients undergoing total hip and total knee arthroplasty. Thromb Res. 2014;134(3):599-603. doi:10.1016/j.thromres.2014.06.027 25064034

[208] Ma QF, Chu CB, Song HQ. Intravenous versus intra-arterial thrombolysis in ischemic stroke: a systematic review and meta-analysis. PLoS One. 2015;10(1):e0116120. doi:10.1371/journal.pone.0116120 25569136 PMC4287629

[209] Mackeen AD, Seibel-Seamon J, Grimes-Dennis J, Baxter JK, Berghella V. Tocolytics for preterm premature rupture of membranes. Cochrane Database Syst Rev. 2011;(10):CD007062. doi:10.1002/14651858.CD007062.pub2 21975760

[210] Mao M, Chen C. Corticosteroid therapy for management of hemolysis, elevated liver enzymes, and low platelet count (HELLP) syndrome: a meta-analysis. Med Sci Monit. 2015;21:3777-3783. doi:10.12659/MSM.895220 26633822 PMC4672720

[211] Matthews E, Brassington R, Kuntzer T, Jichi F, Manzur AY. Corticosteroids for the treatment of Duchenne muscular dystrophy. Cochrane Database Syst Rev. 2016;2016(5):CD003725. doi:10.1002/14651858.CD003725.pub4 27149418 PMC8580515

[212] Mbeye NM, ter Kuile FO, Davies MA, Phiri KS, Egger M, Wandeler G; IeDEA-Southern Africa. Cotrimoxazole prophylactic treatment prevents malaria in children in sub-Saharan Africa: systematic review and meta-analysis. Trop Med Int Health. 2014;19(9):1057-1067. doi:10.1111/tmi.12352 25039469 PMC4127108

[213] Meduri M, Gregoraci G, Baglivo V, Balestrieri M, Isola M, Brambilla P. A meta-analysis of efficacy and safety of aripiprazole in adult and pediatric bipolar disorder in randomized controlled trials and observational studies. J Affect Disord. 2016;191:187-208. doi:10.1016/j.jad.2015.11.033 26674213

[214] Merlotti C, Morabito A, Pontiroli AE. Prevention of type 2 diabetes; a systematic review and meta-analysis of different intervention strategies. Diabetes Obes Metab. 2014;16(8):719-727. doi:10.1111/dom.12270 24476122

[215] Mesfin YM, Kibret KT, Taye A. Is protease inhibitors based antiretroviral therapy during pregnancy associated with an increased risk of preterm birth? systematic review and a meta-analysis. Reprod Health. 2016;13:30. doi:10.1186/s12978-016-0149-5 27048501 PMC4822312

[216] Mesgarpour B, Heidinger BH, Roth D, Schmitz S, Walsh CD, Herkner H. Harms of off-label erythropoiesis-stimulating agents for critically ill people. Cochrane Database Syst Rev. 2017;8(8):CD010969. doi:10.1002/14651858.CD010969.pub2 28841235 PMC6373621

[217] Miyake Y, Iwasaki Y, Yamamoto K. Meta-analysis: reduced incidence of hepatocellular carcinoma in patients not responding to interferon therapy of chronic hepatitis C. Int J Cancer. 2010;127(4):989-996. doi:10.1002/ijc.25090 19957327

[218] Moraes VY, Lenza M, Tamaoki MJ, Faloppa F, Belloti JC. Platelet-rich therapies for musculoskeletal soft tissue injuries. Cochrane Database Syst Rev. 2014;2014(4):CD010071. doi:10.1002/14651858.CD010071.pub3 24782334 PMC6464921

[219] Muanda FT, Chaabane S, Boukhris T, . Antimalarial drugs for preventing malaria during pregnancy and the risk of low birth weight: a systematic review and meta-analysis of randomized and quasi-randomized trials. BMC Med. 2015;13:193. doi:10.1186/s12916-015-0429-x 26275820 PMC4537579

[220] Munnee K, Bundhun PK, Quan H, Tang Z. Comparing the clinical outcomes between insulin-treated and non-insulin-treated patients with type 2 diabetes mellitus after coronary artery bypass surgery: a systematic review and meta-analysis. Medicine (Baltimore). 2016;95(10):e3006. doi:10.1097/MD.0000000000003006 26962814 PMC4998895

[221] Muranushi C, Olsen CM, Pandeya N, Green AC. Aspirin and nonsteroidal anti-inflammatory drugs can prevent cutaneous squamous cell carcinoma: a systematic review and meta-analysis. J Invest Dermatol. 2015;135(4):975-983. doi:10.1038/jid.2014.531 25521453

[222] Muranushi C, Olsen CM, Green AC. Pandeya N. Can oral nonsteroidal antiinflammatory drugs play a role in the prevention of basal cell carcinoma? a systematic review and meta-analysis. J Am Acad Dermatol. 2016. doi:10.1016/j.jaad.2015.08.034 26433247

[223] Murphy GR, Gardiner MD, Glass GE, Kreis IA, Jain A, Hettiaratchy S. Meta-analysis of antibiotics for simple hand injuries requiring surgery. Br J Surg. 2016;103(5):487-492. doi:10.1002/bjs.10111 26928808

[224] Muzii L, Di Tucci C, Achilli C, . Continuous versus cyclic oral contraceptives after laparoscopic excision of ovarian endometriomas: a systematic review and metaanalysis. Am J Obstet Gynecol. 2016;214(2):203-211. doi:10.1016/j.ajog.2015.08.074 26364832

[225] Nairooz R, Valgimigli M, Rochlani Y, . Meta-analysis of clopidogrel pretreatment in acute coronary syndrome patients undergoing invasive strategy. Int J Cardiol. 2017;229:82-89. doi:10.1016/j.ijcard.2016.11.226 27887802

[226] Neufeld KJ, Yue J, Robinson TN, Inouye SK, Needham DM. Antipsychotic medication for prevention and treatment of delirium in hospitalized adults: a systematic review and meta-analysis. J Am Geriatr Soc. 2016;64(4):705-714. doi:10.1111/jgs.14076 27004732 PMC4840067

[227] Niafar M, Hai F, Porhomayon J, Nader ND. The role of metformin on vitamin B12 deficiency: a meta-analysis review. Intern Emerg Med. 2015;10(1):93-102. doi:10.1007/s11739-014-1157-5 25502588

[228] Nie M, Wang Y, Bi XW, . Effect of rituximab on adult Burkitt’s lymphoma: a systematic review and meta-analysis. Ann Hematol. 2016;95(1):19-26. doi:10.1007/s00277-015-2501-1 26423805

[229] O’Brien J, Jackson JW, Grodstein F, Blacker D, Weuve J. Postmenopausal hormone therapy is not associated with risk of all-cause dementia and Alzheimer’s disease. Epidemiol Rev. 2014;36(1):83-103. doi:10.1093/epirev/mxt008 24042430 PMC3873843

[230] Ogunlesi TA, Odigwe CC, Oladapo OT. Adjuvant corticosteroids for reducing death in neonatal bacterial meningitis. Cochrane Database Syst Rev. 2015;2015(11):CD010435. doi:10.1002/14651858.CD010435.pub2 26560739 PMC10542916

[231] Ohlsson A, Lacy JB. Intravenous immunoglobulin for suspected or proven infection in neonates. Cochrane Database Syst Rev. 2015;(3):CD001239. doi:10.1002/14651858.CD001239.pub5 25815707

[232] Okoli GN, Otete HE, Beck CR, Nguyen-Van-Tam JS. Use of neuraminidase inhibitors for rapid containment of influenza: a systematic review and meta-analysis of individual and household transmission studies. PLoS One. 2014;9(12):e113633. doi:10.1371/journal.pone.0113633 25490762 PMC4260958

[233] Ortiz-Orendain J, Castiello-de Obeso S, Colunga-Lozano LE, Hu Y, Maayan N, Adams CE. Antipsychotic combinations for schizophrenia. Cochrane Database Syst Rev. 2017;6(6):CD009005. 28658515 10.1002/14651858.CD009005.pub2 PMC6481822

[234] Ortiz-Salas P, Velez-Van-Meerbeke A, Galvis-Gomez CA, Rodriguez Q JH. Human immunoglobulin versus plasmapheresis in Guillain-Barre syndrome and myasthenia gravis: a meta-analysis. J Clin Neuromuscul Dis. 2016;18(1):1-11. doi:10.1097/CND.0000000000000119 27552383

[235] Osborn DA, Jeffery HE, Cole MJ. Opiate treatment for opiate withdrawal in newborn infants. Cochrane Database Syst Rev. 2010;(10):CD002059. doi:10.1002/14651858.CD002059.pub3 20927730

[236] Osborn DA, Jeffery HE, Cole MJ. Sedatives for opiate withdrawal in newborn infants. Cochrane Database Syst Rev. 2010;(10):CD002053. doi:10.1002/14651858.CD002053.pub3 20927729

[237] Paciaroni M, Agnelli G, Venti M, Alberti A, Acciarresi M, Caso V. Efficacy and safety of anticoagulants in the prevention of venous thromboembolism in patients with acute cerebral hemorrhage: a meta-analysis of controlled studies. J Thromb Haemost. 2011;9(5):893-898. doi:10.1111/j.1538-7836.2011.04241.x 21324058

[238] Pammi M, Haque KN. Pentoxifylline for treatment of sepsis and necrotizing enterocolitis in neonates. Cochrane Database Syst Rev. 2015;(3):CD004205. doi:10.1002/14651858.CD004205.pub3 25751631

[239] Pan W, Wang Y, Lin L, Zhou G, Hua X, Mo L. Outcomes of dexmedetomidine treatment in pediatric patients undergoing congenital heart disease surgery: a meta-analysis. Paediatr Anaesth. 2016;26(3):239-248. doi:10.1111/pan.12820 26612740

[240] Pan X, Zhu Y, Zheng D, Liu Y, Yu F, Yang J. Prior antiplatelet agent use and outcomes after intravenous thrombolysis with recombinant tissue plasminogen activator in acute ischemic stroke: a meta-analysis of cohort studies and randomized controlled trials. Int J Stroke. 2015;10(3):317-323. doi:10.1111/ijs.12431 25545076

[241] Pani PP, Trogu E, Pacini M, Maremmani I. Anticonvulsants for alcohol dependence. Cochrane Database Syst Rev. 2014;2014(2):CD008544. 24523233 10.1002/14651858.CD008544.pub2 PMC10585425

[242] Paul M, Dickstein Y, Raz-Pasteur A. Antibiotic de-escalation for bloodstream infections and pneumonia: systematic review and meta-analysis. Clin Microbiol Infect. 2016;22(12):960-967. doi:10.1016/j.cmi.2016.05.023 27283148

[243] Paul M, Lador A, Grozinsky-Glasberg S, Leibovici L. Beta lactam antibiotic monotherapy versus beta lactam-aminoglycoside antibiotic combination therapy for sepsis. Cochrane Database Syst Rev. 2014;2014(1):CD003344. doi:10.1002/14651858.CD003344.pub3 24395715 PMC6517128

[244] Paul S, Saxena A, Terrin N, Viveiros K, Balk EM, Wong JB. Hepatitis B virus reactivation and prophylaxis during solid tumor chemotherapy: a systematic review and meta-analysis. Ann Intern Med. 2016;164(1):30-40. doi:10.7326/M15-1121 26595058 PMC6410701

[245] Pérez-Gaxiola G, Cuello-García CA, Florez ID, Pérez-Pico VM. Smectite for acute infectious diarrhoea in children. Cochrane Database Syst Rev. 2018;4(4):CD011526. doi:10.1002/14651858.CD011526.pub2 29693719 PMC6494641

[246] Peters R, Booth A, Peters J. A systematic review of calcium channel blocker use and cognitive decline/dementia in the elderly. J Hypertens. 2014;32(10):1945-1957. doi:10.1097/HJH.0000000000000273 25068540

[247] Prasad M, Krishnan PR, Sequeira R, Al-Roomi K. Anticonvulsant therapy for status epilepticus. Cochrane Database Syst Rev. 2014;2014(9):CD003723. 25207925 10.1002/14651858.CD003723.pub3 PMC7154380

[248] Price J, Leng GC. Steroid sex hormones for lower limb atherosclerosis. Cochrane Database Syst Rev. 2012;10(10):CD000188. doi:10.1002/14651858.CD000188.pub2 23076884 PMC6956560

[249] Prijic S, Buchhorn R, Kosutic J, . Beta-blockers (carvedilol) in children with systemic ventricle systolic dysfunction - systematic review and meta-analysis. Rev Recent Clin Trials. 2014;9(2):68-75. doi:10.2174/1574887109666140908125640 25198735

[250] Prins KW, Neill JM, Tyler JO, Eckman PM, Duval S. Effects of beta-blocker withdrawal in acute decompensated heart failure: a systematic review and meta-analysis. JACC Heart Fail. 2015;3(8):647-653. doi:10.1016/j.jchf.2015.03.008 26251094 PMC4777602

[251] Proietti R, Porto I, Levi M, . Risk of pocket hematoma in patients on chronic anticoagulation with warfarin undergoing electrophysiological device implantation: a comparison of different peri-operative management strategies. Eur Rev Med Pharmacol Sci. 2015;19(8):1461-1479. 25967723

[252] Prutsky G, Domecq JP, Mori L, . Treatment outcomes of human bartonellosis: a systematic review and meta-analysis. Int J Infect Dis. 2013;17(10):e811-e819. doi:10.1016/j.ijid.2013.02.016 23602630

[253] Puig I, Baylina M, Sánchez-Delgado J, . Systematic review and meta-analysis: triple therapy combining a proton-pump inhibitor, amoxicillin and metronidazole for Helicobacter pylori first-line treatment. J Antimicrob Chemother. 2016;71(10):2740-2753. doi:10.1093/jac/dkw220 27342548

[254] Qin AQ, Liang ZG, Ye JX, . Significant efficacy of additional concurrent chemotherapy with radiotherapy for postoperative cervical cancer with risk factors: a systematic review and meta-analysis. Asian Pac J Cancer Prev. 2016;17(8):3945-3951. 27644643

[255] Qiu JL, Shao MY, Xie WF, . Effect of combined ursodeoxycholic acid and glucocorticoid on the outcome of Kasai procedure: a systematic review and meta-analysis. Medicine (Baltimore). 2018;97(35):e12005. doi:10.1097/MD.0000000000012005 30170405 PMC6393119

[256] Qiu Y, Mao R, Chen BL, . Effects of combination therapy with immunomodulators on trough levels and antibodies against tumor necrosis factor antagonists in patients with inflammatory bowel disease: a meta-analysis. Clin Gastroenterol Hepatol. 2017;15(9):1359-1372.e6. doi:10.1016/j.cgh.2017.02.005 28232073

[257] Radeva-Petrova D, Kayentao K, ter Kuile FO, Sinclair D, Garner P. Drugs for preventing malaria in pregnant women in endemic areas: any drug regimen versus placebo or no treatment. Cochrane Database Syst Rev. 2014;2014(10):CD000169. doi:10.1002/14651858.CD000169.pub3 25300703 PMC4498495

[258] Rivero A, Liang J. Anti-IgE and anti-IL5 biologic therapy in the treatment of nasal polyposis: a systematic review and meta-analysis. Ann Otol Rhinol Laryngol. 2017;126(11):739-747. doi:10.1177/0003489417731782 28918644

[259] Roberts MJ, Scott S, Harris PN, Naber K, Wagenlehner FME, Doi SAR. Comparison of fosfomycin against fluoroquinolones for transrectal prostate biopsy prophylaxis: an individual patient-data meta-analysis. World J Urol. 2018;36(3):323-330. doi:10.1007/s00345-017-2163-9 29288398

[260] Rodriguez-Zuniga M, Torres N, Garcia-Perdomo H. Effectiveness of acyclovir in the treatment of pityriasis rosea. a systematic review and meta-analysis. An Bras Dermatol. 2018;93(5):686-695. doi:10.1590/abd1806-4841.20187252 30156618 PMC6106661

[261] Rojas-Villarraga A, Torres-Gonzalez JV, Ruiz-Sternberg AM. Safety of hormonal replacement therapy and oral contraceptives in systemic lupus erythematosus: a systematic review and meta-analysis. PLoS One. 2014;9(8):e104303. doi:10.1371/journal.pone.0104303 25137236 PMC4138076

[262] Rokkas T, Portincasa P. Colon neoplasia in patients with type 2 diabetes on metformin: a meta-analysis. Eur J Intern Med. 2016;33:60-66 doi:10.1016/j.ejim.2016.05.027 27318643

[263] Rys PM, Ludwig-Slomczynska AH, Cyganek K, Malecki MT. Continuous subcutaneous insulin infusion vs multiple daily injections in pregnant women with type 1 diabetes mellitus: a systematic review and meta-analysis of randomised controlled trials and observational studies. Eur J Endocrinol. 2018;178(5):545-563. doi:10.1530/EJE-17-0804 29545258

[264] Sahebkar A, Reiner Z, Simental-Mendia LE, Ferretti G, Della Corte C, Nobili V. Impact of statin therapy on plasma vitamin d levels: a systematic review and meta-analysis. Curr Pharm Des. 2017;23(6):861-869. doi:10.2174/1381612822666161006150542 27719645

[265] Sahebkar A, Serban MC, Penson P, ; Lipid and Blood Pressure Meta-analysis Collaboration (LBPMC) Group. The effects of tamoxifen on plasma lipoprotein(a) concentrations: systematic review and meta-analysis. Drugs. 2017;77(11):1187-1197. doi:10.1007/s40265-017-0767-4 28573436 PMC5501893

[266] Salata K, Syed M, Hussain MA, . Renin-angiotensin system blockade does not attenuate abdominal aortic aneurysm growth, rupture rate, or perioperative mortality after elective repair. J Vasc Surg. 2018;67(2):629-636.e2. doi:10.1016/j.jvs.2017.09.007 29175038

[267] Salvi V, Grua I, Cerveri G, Mencacci C, Barone-Adesi F. The risk of new-onset diabetes in antidepressant users - A systematic review and meta-analysis. PLoS One. 2017;12(7):e0182088. doi:10.1371/journal.pone.0182088 28759599 PMC5536271

[268] Sant’anna RT, Leiria TL, Nascimento T, . Meta-analysis of continuous oral anticoagulants versus heparin bridging in patients undergoing CIED surgery: reappraisal after the BRUISE study. Pacing Clin Electrophysiol. 2015;38(4):417-423. doi:10.1111/pace.12557 25546244

[269] Sardar P, Nairooz R, Chatterjee S, Wetterslev J, Ghosh J, Aronow WS. Meta-analysis of risk of stroke or transient ischemic attack with dabigatran for atrial fibrillation ablation. Am J Cardiol. 2014;113(7):1173-1177. doi:10.1016/j.amjcard.2013.12.027 24513472

[270] Serpa Neto A, Veelo DP, Peireira VG, . Fluid resuscitation with hydroxyethyl starches in patients with sepsis is associated with an increased incidence of acute kidney injury and use of renal replacement therapy: a systematic review and meta-analysis of the literature. J Crit Care. 2014;29(1):185.e1-185.e7. doi:10.1016/j.jcrc.2013.09.031 24262273

[271] Seth R, Kydd AS, Buchbinder R, Bombardier C, Edwards CJ. Allopurinol for chronic gout. Cochrane Database Syst Rev. 2014;2014(10):CD006077. 25314636 10.1002/14651858.CD006077.pub3 PMC8915170

[272] Shang PF, Kwong J, Wang ZP, . Intravesical Bacillus Calmette-Guérin versus epirubicin for Ta and T1 bladder cancer. Cochrane Database Syst Rev. 2011;(5):CD006885. doi:10.1002/14651858.CD006885.pub2 21563157 PMC13378883

[273] Sharma A, Einstein AJ, Vallakati A, . Risk of atrial fibrillation with use of oral and intravenous bisphosphonates. Am J Cardiol. 2014;113(11):1815-1821. doi:10.1016/j.amjcard.2014.03.008 24837258

[274] Shen J, Huang YM, Wang M, . Renin-angiotensin system blockade for the risk of cancer and death. J Renin Angiotensin Aldosterone Syst. Published online, July 8, 2016. doi:10.1177/1470320316656679 27402638 PMC5843874

[275] Shi L, Xu L, Shi L, Brandon D, Chen S, Zhang J. Intraventricular recombinant tissue plasminogen activator in treatment of aneurysmal intraventricular hemorrhage: a meta-analysis. Curr Drug Targets. 2017;18(12):1399-1407. doi:10.2174/1389450116666150907110815 26343113

[276] Shi M, Zheng H, Nie B, Gong W, Cui X. Statin use and risk of liver cancer: an update meta-analysis. BMJ Open. 2014;4(9):e005399. doi:10.1136/bmjopen-2014-005399 25227628 PMC4166249

[277] Shim SH, Lee SJ, Kim SN. Effects of hormone replacement therapy on the rate of recurrence in endometrial cancer survivors: a meta-analysis. Eur J Cancer. 2014;50(9):1628-1637. doi:10.1016/j.ejca.2014.03.006 24685478

[278] Shin JY, Kim JS. Could 5-fluorouracil or triamcinolone be an effective treatment option for keloid after surgical excision? a meta-analysis. J Oral Maxillofac Surg. 2016;74(5):1055-1060. doi:10.1016/j.joms.2015.10.002 26529198

[279] Sim LA, McGovern L, Elamin MB, Swiglo BA, Erwin PJ, Montori VM. Effect on bone health of estrogen preparations in premenopausal women with anorexia nervosa: a systematic review and meta-analyses. Int J Eat Disord. 2010;43(3):218-225. doi:10.1002/eat.20687 19350651

[280] Sinclair D, Abba K, Zaman K, Qadri F, Graves PM. Oral vaccines for preventing cholera. Cochrane Database Syst Rev. 2011;2011(3):CD008603. 21412922 10.1002/14651858.CD008603.pub2 PMC6532691

[281] Singh P, Madanipour S, Bhamra JS, Gill I. A systematic review and meta-analysis of platelet-rich plasma versus corticosteroid injections for plantar fasciopathy. Int Orthop. 2017;41(6):1169-1181. doi:10.1007/s00264-017-3470-x 28396927

[282] Singh PM, Borle A, Trikha A, Michos L, Sinha A, Goudra B. Role of periarticular liposomal bupivacaine infiltration in patients undergoing total knee arthroplasty-a meta-analysis of comparative trials. J Arthroplasty. 2017;32(2):675-688.e1. doi:10.1016/j.arth.2016.09.042 28029532

[283] Smaill FM, Grivell RM. Antibiotic prophylaxis versus no prophylaxis for preventing infection after cesarean section. Cochrane Database Syst Rev. 2014;2014(10):CD007482. doi:10.1002/14651858.CD007482.pub3 25350672 PMC8078551

[284] Smit E, Odd D, Whitelaw A. Postnatal phenobarbital for the prevention of intraventricular haemorrhage in preterm infants. Cochrane Database Syst Rev. 2013;2013(8):CD001691. doi:10.1002/14651858.CD001691.pub3 23943189 PMC7061244

[285] Solé-Lleonart C, Rouby JJ, Blot S, . Nebulization of antiinfective agents in invasively mechanically ventilated adults: a systematic review and meta-analysis. Anesthesiology. 2017;126(5):890-908. doi:10.1097/ALN.0000000000001570 28248714

[286] Song T, Choi CH, Kim MK, Kim ML, Yun BS, Seong SJ. The effect of angiotensin system inhibitors (angiotensin-converting enzyme inhibitors or angiotensin receptor blockers) on cancer recurrence and survival: a meta-analysis. Eur J Cancer Prev. 2017;26(1):78-85. doi:10.1097/CEJ.0000000000000269 27158979

[287] Sotiriadis A, Tsiami A, Papatheodorou S, Baschat AA, Sarafidis K, Makrydimas G. Neurodevelopmental outcome after a single course of antenatal steroids in children born preterm: a systematic review and meta-analysis. Obstet Gynecol. 2015;125(6):1385-1396. doi:10.1097/AOG.0000000000000748 26000510

[288] Squizzato A, Galli M, Romualdi E, . Statins, fibrates, and venous thromboembolism: a meta-analysis. Eur Heart J. 2010;31(10):1248-1256. doi:10.1093/eurheartj/ehp556 20031958

[289] Stern A, Green H, Paul M, Vidal L, Leibovici L. Prophylaxis for pneumocystis pneumonia (PCP) in non-HIV immunocompromised patients. Cochrane Database Syst Rev. 2014;2014(10):CD005590. doi:10.1002/14651858.CD005590.pub3 25269391 PMC6457644

[290] Strohmeier Y, Hodson EM, Willis NS, Webster AC, Craig JC. Antibiotics for acute pyelonephritis in children. Cochrane Database Syst Rev. 2014;2014(7):CD003772. 25066627 10.1002/14651858.CD003772.pub4 PMC10580126

[291] Suthar AB, Vitoria MA, Nagata JM, . Co-trimoxazole prophylaxis in adults, including pregnant women, with HIV: a systematic review and meta-analysis. Lancet HIV. 2015;2(4):e137-e150. doi:10.1016/S2352-3018(15)00005-3 26424674

[292] Talukdar R, Murthy HV, Reddy DN. Role of methionine containing antioxidant combination in the management of pain in chronic pancreatitis: a systematic review and meta-analysis. Pancreatology. 2015;15(2):136-144. doi:10.1016/j.pan.2015.01.003 25648074

[293] Tang BM, Craig JC, Eslick GD, Seppelt I, McLean AS. Use of corticosteroids in acute lung injury and acute respiratory distress syndrome: a systematic review and meta-analysis. Crit Care Med. 2009;37(5):1594-1603. doi:10.1097/CCM.0b013e31819fb507 19325471

[294] Tang X, Yang Y, Luo S, . The effect of statin therapy on plaque regression following acute coronary syndrome: a meta-analysis of prospective trials. Coron Artery Dis. 2016;27(8):636-649. doi:10.1097/MCA.0000000000000403 27388482

[295] Tarantini G, Ueshima D, D’Amico G, . Efficacy and safety of potent platelet P2Y12 receptor inhibitors in elderly versus nonelderly patients with acute coronary syndrome: a systematic review and meta-analysis. Am Heart J. 2018;195:78-85. doi:10.1016/j.ahj.2017.09.012 29224649

[296] Taylor JE, Tan K, Lai NM, McDonald SJ. Antibiotic lock for the prevention of catheter-related infection in neonates. Cochrane Database Syst Rev. 2015;2015(6):CD010336. doi:10.1002/14651858.CD010336.pub2 26040840 PMC10577674

[297] Teng Y, Ma J, Ma X, Wang Y, Lu B, Guo C. The efficacy and safety of epinephrine for postoperative bleeding in total joint arthroplasty: a PRISMA-compliant meta-analysis. Medicine (Baltimore). 2017;96(17):e6763. doi:10.1097/MD.0000000000006763 28445306 PMC5413271

[298] Toews I, George AT, Peter JV, . Interventions for preventing upper gastrointestinal bleeding in people admitted to intensive care units. Cochrane Database Syst Rev. 2018;6(6):CD008687. doi:10.1002/14651858.CD008687.pub2 29862492 PMC6513395

[299] Tran-Duy A, Spaetgens B, Hoes AW, de Wit NJ, Stehouwer CD. Use of proton pump inhibitors and risks of fundic gland polyps and gastric cancer: systematic review and meta-analysis. Clin Gastroenterol Hepatol. 2016;14(12):1706-1719.e5. doi:10.1016/j.cgh.2016.05.018 27211501

[300] Tsai CC, Yang PS, Liu CL, Wu CJ, Hsu YC, Cheng SP. Comparison of topical mupirocin and gentamicin in the prevention of peritoneal dialysis-related infections: a systematic review and meta-analysis. Am J Surg. 2018;215(1):179-185. doi:10.1016/j.amjsurg.2017.03.005 28341139

[301] Tsaousi GG, Lamperti M, Bilotta F. Role of dexmedetomidine for sedation in neurocritical care patients: a qualitative systematic review and meta-analysis of current evidence. Clin Neuropharmacol. 2016;39(3):144-151. doi:10.1097/WNF.0000000000000151 27046655

[302] Tully PJ, Hanon O, Cosh S, Tzourio C. Diuretic antihypertensive drugs and incident dementia risk: a systematic review, meta-analysis and meta-regression of prospective studies. J Hypertens. 2016;34(6):1027-1035. doi:10.1097/HJH.0000000000000868 26886565

[303] Tunnicliffe DJ, Palmer SC, Henderson L, . Immunosuppressive treatment for proliferative lupus nephritis. Cochrane Database Syst Rev. 2018;6(6):CD002922. 29957821 10.1002/14651858.CD002922.pub4 PMC6513226

[304] Turgeon RD, Barry AR. Single vs dual antiplatelet therapy following transcatheter aortic valve implantation: a systematic review. Clin Cardiol. 2015;38(10):629-634. doi:10.1002/clc.22426 26239886 PMC6490860

[305] Ukaigwe A, Shrestha P, Karmacharya P, . Meta-analysis of efficacy and safety of apixaban and uninterrupted apixaban therapy compared to vitamin K antagonists in patients undergoing catheter ablation for atrial fibrillation. J Interv Card Electrophysiol. 2017;48(2):223-233. doi:10.1007/s10840-016-0195-5 27771820

[306] van Herwaarden N, den Broeder AA, Jacobs W, . Down-titration and discontinuation strategies of tumor necrosis factor-blocking agents for rheumatoid arthritis in patients with low disease activity. Cochrane Database Syst Rev. 2014;(9):CD010455. doi:10.1002/14651858.CD010455.pub2 25264908

[307] Vardakas KZ, Mavroudis AD, Georgiou M, Falagas ME. Intravenous colistin combination antimicrobial treatment vs. monotherapy: a systematic review and meta-analysis. Int J Antimicrob Agents. 2018;51(4):535-547. doi:10.1016/j.ijantimicag.2017.12.020 29288723

[308] Vecchio M, Bonerba B, Palmer SC, . Immunosuppressive agents for treating IgA nephropathy. Cochrane Database Syst Rev. 2015;(8):CD003965. 26235292 10.1002/14651858.CD003965.pub2

[309] Vyas A, El Accaoui R, Blevins A, Karrowni W. Outcome comparison of 600 mg versus 300 mg loading dose of clopidogrel for patients with ST-elevation myocardial infarction: a meta-analysis. Postgrad Med. 2014;126(5):176-186. doi:10.3810/pgm.2014.09.2812 25295662

[310] Wan Q, Li L, Yang S, Chu F. Impact of statins on arteriovenous fistulas outcomes: a meta-analysis. Ther Apher Dial. 2018;22(1):67-72. doi:10.1111/1744-9987.12597 28960860

[311] Wang H, Zhang L, Jin Y. A meta-analysis of the protective effect of recombinant human erythropoietin (rhEPO) for neurodevelopment in preterm infants. Cell Biochem Biophys. 2015;71(2):795-802. doi:10.1007/s12013-014-0265-1 25300689

[312] Wang HS, Wang ZW, Yin ZT. Carvedilol for prevention of atrial fibrillation after cardiac surgery: a meta-analysis. PLoS One. 2014;9(4):e94005. doi:10.1371/journal.pone.0094005 24705913 PMC3976381

[313] Wang J, Wang Q, Wu Q, Chen Y, Wu P. Intravesical botulinum toxin a injections for bladder pain syndrome/interstitial cystitis: a systematic review and meta-analysis of controlled studies. Med Sci Monit. 2016;22:3257-3267. doi:10.12659/MSM.897350 27624897 PMC5032852

[314] Wang J, Zhu L, Hu K, . Effects of metformin treatment on serum levels of C-reactive protein and interleukin-6 in women with polycystic ovary syndrome: a meta-analysis: a PRISMA-compliant article. Medicine (Baltimore). 2017;96(39):e8183. doi:10.1097/MD.0000000000008183 28953677 PMC5626320

[315] Wang M, Tan H, Wu Z, Liang Y. The efficacy and safety of anti-fibrinolytic agents in blood management following peri-acetabular osteotomy: a meta-analysis. Medicine (Baltimore). 2018;97(34):e11967. doi:10.1097/MD.0000000000011967 30142824 PMC6112897

[316] Wang SQ, Zhang LW, Wei P, Hua H. Is hydroxychloroquine effective in treating primary Sjogren’s syndrome: a systematic review and meta-analysis. BMC Musculoskelet Disord. 2017;18(1):186. doi:10.1186/s12891-017-1543-z 28499370 PMC5427554

[317] Wang W, Shi M, Zhou C, . Effectiveness of corticosteroid injections in adhesive capsulitis of shoulder: a meta-analysis. Medicine (Baltimore). 2017;96(28):e7529. doi:10.1097/MD.0000000000007529 28700506 PMC5515778

[318] Wang WN, Wu MY, Ma FZ, Sun T, Xu ZG. Meta-analysis of the efficacy and safety of nucleotide/nucleoside analog monotherapy for hepatitis B virus-associated glomerulonephritis. Clin Nephrol. 2016;85(1):21-29. doi:10.5414/CN108648 26636326

[319] Watti H, Dahal K, Zabher HG, Katikaneni P, Modi K, Abdulbaki A. Comparison of prasugrel and ticagrelor in patients with acute coronary syndrome undergoing percutaneous coronary intervention: a meta-analysis of randomized and non-randomized studies. Int J Cardiol. 2017;249:66-72. doi:10.1016/j.ijcard.2017.07.103 29121759

[320] Westhoff G, Cotter AM, Tolosa JE. Prophylactic oxytocin for the third stage of labour to prevent postpartum haemorrhage. Cochrane Database Syst Rev. 2013;(10):CD001808. doi:10.1002/14651858.CD001808.pub2 24173606

[321] Whiting P, Morden A, Tomlinson LA, . What are the risks and benefits of temporarily discontinuing medications to prevent acute kidney injury? a systematic review and meta-analysis. BMJ Open. 2017;7(4):e012674. doi:10.1136/bmjopen-2016-012674 28389482 PMC5541442

[322] Widmer M, Lopez I, Gülmezoglu AM, Mignini L, Roganti A. Duration of treatment for asymptomatic bacteriuria during pregnancy. Cochrane Database Syst Rev. 2015;2015(11):CD000491. doi:10.1002/14651858.CD000491.pub3 26560337 PMC7043273

[323] Wilhelmus KR. Antiviral treatment and other therapeutic interventions for herpes simplex virus epithelial keratitis. Cochrane Database Syst Rev. 2015;1(1):CD002898. doi:10.1002/14651858.CD002898.pub5 25879115 PMC4443501

[324] Wiysonge CS, Ntsekhe M, Thabane L, . Interventions for treating tuberculous pericarditis. Cochrane Database Syst Rev. 2017;9(9):CD000526. 28902412 10.1002/14651858.CD000526.pub2 PMC5618454

[325] Wu L, Zhu J, Prokop LJ, Murad MH. Pharmacologic therapy of diabetes and overall cancer risk and mortality: a meta-analysis of 265 studies. Sci Rep. 2015;5:10147. doi:10.1038/srep10147 26076034 PMC4467243

[326] Wu Z, Zhang H, Jin W, . The effect of renin-angiotensin-aldosterone system blockade medications on contrast-induced nephropathy in patients undergoing coronary angiography: a meta-analysis. PLoS One. 2015;10(6):e0129747. doi:10.1371/journal.pone.0129747 26083525 PMC4470628

[327] Xia BW, Zhang YC, Wang J, Ding FH, He XD. Efficacy of antiviral therapy with nucleotide/nucleoside analogs after curative treatment for patients with hepatitis B virus-related hepatocellular carcinoma: a systematic review and meta-analysis. Clin Res Hepatol Gastroenterol. 2015;39(4):458-468. doi:10.1016/j.clinre.2014.12.003 25650304

[328] Xia P, Wang X, Lin Q, Li X. Efficacy of mesenchymal stem cells injection for the management of knee osteoarthritis: a systematic review and meta-analysis. Int Orthop. 2015;39(12):2363-2372. doi:10.1007/s00264-015-2785-8 25944079

[329] Xie Y, Zhang T, Tian Z, . Efficacy of intrauterine perfusion of granulocyte colony-stimulating factor (G-CSF) for infertile women with thin endometrium: a systematic review and meta-analysis. Am J Reprod Immunol. 2017;78(2). doi:10.1111/aji.12701 28497881

[330] Xing F, Hu X, Jiang J, Ma Y, Tang A. A meta-analysis of low-dose dopamine in heart failure. Int J Cardiol. 2016;222:1003-1011. doi:10.1016/j.ijcard.2016.07.262 27526385

[331] Xiong Q, Li Z, Li Z, . Anti-VEGF agents with or without antimetabolites in trabeculectomy for glaucoma: a meta-analysis. PLoS One. 2014;9(2):e88403. doi:10.1371/journal.pone.0088403 24523890 PMC3921170

[332] Xu SX, Shen JL, Tang XF, Feng B, Xu HQ. Newer antifungal agents micafungin and voriconazole for fungal infection prevention during hematopoietic cell transplantation: a meta-analysis. Eur Rev Med Pharmacol Sci. 2016;20(2):381-390. 26875911

[333] Yang C, Qin B, Yuan Z, Chen L, Zhou HY. Meta-analysis of prophylactic entecavir or lamivudine against hepatitis B virus reactivation. Ann Hepatol. 2016;15(4):501-511. 27236149

[334] Yang H, Cui X, Ma Z, Liu L. Evaluation outcomes associated with alternative dosing strategies for piperacillin/tazobactam: a systematic review and meta-analysis. J Pharm Pharm Sci. 2016;19(2):274-289. doi:10.18433/J33S4B 27518175

[335] Yang S, Shi Q, Liu J, Li J, Xu J. Should oral anticoagulant therapy be continued during dental extraction? a meta-analysis. BMC Oral Health. 2016;16(1):81. doi:10.1186/s12903-016-0278-9 27566540 PMC5002166

[336] Yang Y, Ma YP, Chen DP, Zhuo L, Li WG. A meta-analysis of antiviral therapy for hepatitis b virus-associated membranous nephropathy. PLoS One. 2016;11(9):e0160437. doi:10.1371/journal.pone.0160437 27598699 PMC5012684

[337] Yang Z, Hackshaw A, Feng Q, . Comparison of gefitinib, erlotinib and afatinib in non-small cell lung cancer: a meta-analysis. Int J Cancer. 2017;140(12):2805-2819. doi:10.1002/ijc.30691 28295308

[338] Yao Z, Ma L, You C. Antiepileptic drugs for patients with intracerebral hemorrhage: a meta-analysis. Turk Neurosurg. 2018;28(3):389-393. 27873294 10.5137/1019-5149.JTN.18791-16.2

[339] Ye R, Hu Y, Yao A, . Impact of renin-angiotensin system-targeting antihypertensive drugs on treatment of Alzheimer’s disease: a meta-analysis. Int J Clin Pract. 2015;69(6):674-681. doi:10.1111/ijcp.12626 25721930

[340] Yin S, He T, Li Y, . Rituximab shows no effect on remission in patients with refractory nephrotic syndrome: A MOOSE-compliant meta-analysis. Medicine (Baltimore). 2016;95(50):e5320. doi:10.1097/MD.0000000000005320 27977574 PMC5268020

[341] Yong JW, Yang LX, Ohene BE, Zhou YJ, Wang ZJ. Periprocedural heparin bridging in patients receiving oral anticoagulation: a systematic review and meta-analysis. BMC Cardiovasc Disord. 2017;17(1):295. doi:10.1186/s12872-017-0719-7 29237411 PMC5729256

[342] Yong M, Zhou M, Deng G. Photodynamic therapy versus anti-vascular endothelial growth factor agents for polypoidal choroidal vasculopathy: a meta-analysis. BMC Ophthalmol. 2015;15:82. doi:10.1186/s12886-015-0064-5 26209516 PMC4513969

[343] Yuan Y, Yunhe M, Xiang W, . P450 enzyme-inducing and non-enzyme-inducing antiepileptic drugs for seizure prophylaxis after glioma resection surgery: a meta-analysis. Seizure. 2014;23(8):616-621. doi:10.1016/j.seizure.2014.04.016 24878104

[344] Zaiem F, Alahdab F, Al Nofal A, Murad MH, Javed A. Oral versus transdermal estrogen in turner syndrome: a systematic review and meta-analysis. Endocr Pract. 2017;23(4):408-421. doi:10.4158/EP161622.OR 28095041

[345] Zeng L, Choonara I, Zhang L, Li Y, Shi J. Effectiveness of prothrombin complex concentrate (PCC) in neonates and infants with bleeding or risk of bleeding: a systematic review and meta-analysis. Eur J Pediatr. 2017;176(5):581-589. doi:10.1007/s00431-017-2877-0 28281092

[346] Zeng XL, Zhang YF, Tian Q, Xue Y, An RF. Effects of metformin on pregnancy outcomes in women with polycystic ovary syndrome: a meta-analysis. Medicine (Baltimore). 2016;95(36):e4526. doi:10.1097/MD.0000000000004526 27603343 PMC5023865

[347] Zhai L, Song Z, Liu K. The effect of gabapentin on acute postoperative pain in patients undergoing total knee arthroplasty: a meta-analysis. Medicine (Baltimore). 2016;95(20):e3673. doi:10.1097/MD.0000000000003673 27196473 PMC4902415

[348] Zhang F, Wang Y, Wang ZQ, . Efficacy and safety of cisplatin-based versus nedaplatin-based regimens for the treatment of metastatic/recurrent and advanced esophageal squamous cell carcinoma: a systematic review and meta-analysis. Dis Esophagus. 2017;30(2):1-8. 27868295 10.1111/dote.12490

[349] Zhang FY, Tang W, Zhang ZZ, Huang JC, Zhang SX, Zhao XC. Systematic review of high-dose and standard-dose chemotherapies in the treatment of primary well-differentiated osteosarcoma. Tumour Biol. 2014;35(10):10419-10427. doi:10.1007/s13277-014-2253-x 25053592

[350] Zhang HW, Lin ZX, Xu C, Leung C, Chan LS. Astragalus (a traditional Chinese medicine) for treating chronic kidney disease. Cochrane Database Syst Rev. 2014;2014(10):CD008369. doi:10.1002/14651858.CD008369.pub2 25335553 PMC10589061

[351] Zhang L, Mendoza-Sassi RA, Wainwright C, Klassen TP. Nebulised hypertonic saline solution for acute bronchiolitis in infants. Cochrane Database Syst Rev. 2017;12(12):CD006458. doi:10.1002/14651858.CD006458.pub4 29265171 PMC6485976

[352] Zhang LK, Ma JX, Kuang MJ, . The efficacy of tranexamic acid using oral administration in total knee arthroplasty: a systematic review and meta-analysis. J Orthop Surg Res. 2017;12(1):159. doi:10.1186/s13018-017-0660-6 29078788 PMC5658985

[353] Zhang LK, Ma JX, Kuang MJ, . Comparison of oral versus intravenous application of tranexamic acid in total knee and hip arthroplasty: a systematic review and meta-analysis. Int J Surg. 2017;45:77-84. doi:10.1016/j.ijsu.2017.07.097 28755884

[354] Zhang M, Niu W, Wang Y, . Dehydroepiandrosterone treatment in women with poor ovarian response undergoing IVF or ICSI: a systematic review and meta-analysis. J Assist Reprod Genet. 2016;33(8):981-991. doi:10.1007/s10815-016-0713-5 27094195 PMC4974220

[355] Zhang MZ, Xun PC, He K, Cai W. Adjuvant steroid treatment following Kasai portoenterostomy and clinical outcomes of biliary atresia patients: an updated meta-analysis. World J Pediatr. 2017;13(1):20-26. doi:10.1007/s12519-016-0052-8 27830578

[356] Zhang Y, Wang ZZ, Sun HM, Li P, Li YF, Chen NH. Systematic review of traditional chinese medicine for depression in Parkinson’s disease. Am J Chin Med. 2014;42(5):1035-1051. doi:10.1142/S0192415X14500657 25183301

[357] Zhang Z, Zhang X, Korantzopoulos P, . Thiazolidinedione use and atrial fibrillation in diabetic patients: a meta-analysis. BMC Cardiovasc Disord. 2017;17(1):96. doi:10.1186/s12872-017-0531-4 28381265 PMC5382449

[358] Zhao J, Li D, Shi Y, . Transarterial infusion chemotherapy with and without embolisation in hepatocellular carcinoma patients: a systematic review and meta-analysis. Ann Acad Med Singap. 2017;46(5):174-184. doi:10.47102/annals-acadmedsg.V46N5p174 28600578

[359] Zhao J, Wang C, Hu Z. Efficacy and safety of bisphosphonates for osteoporosis or osteopenia in cardiac transplant patients: a meta-analysis. Transplant Proc. 2015;47(10):2957-2964. doi:10.1016/j.transproceed.2015.10.049 26707321

[360] Zhao R, Xu Z, Zhao M. Effects of oestrogen treatment on skeletal response to exercise in the hips and spine in postmenopausal women: a meta-analysis. Sports Med. 2015;45(8):1163-1173. doi:10.1007/s40279-015-0338-3 26003475

[361] Zhao SJ, Zhong ZS, Qi GX, Shi LY, Chen L, Tian W. Effect of pioglitazone in preventing in-stent restenosis after percutaneous coronary intervention in patients with type 2 diabetes: a meta-analysis. PLoS One. 2016;11(5):e0155273. doi:10.1371/journal.pone.0155273 27163676 PMC4862640

[362] Zhao XY, Xia S, Wang EQ, Chen YX. Efficacy of intravitreal injection of bevacizumab in vitrectomy for patients with proliferative vitreoretinopathy retinal detachment: a meta-analysis of prospective studies. Retina. 2018;38(3):462-470. doi:10.1097/IAE.0000000000001584 28272285

[363] Zhao Y, Nicoll R, He YH, Henein MY. The effect of statins on valve function and calcification in aortic stenosis: a meta-analysis. Atherosclerosis. 2016;246:318-324. doi:10.1016/j.atherosclerosis.2016.01.023 26828749

[364] Zhao Y, Yang Y, Tang X, Yu X, Zhang L, Xiao H. New oral anticoagulants compared to warfarin for perioperative anticoagulation in patients undergoing atrial fibrillation catheter ablation: a meta-analysis of continuous or interrupted new oral anticoagulants during ablation compared to interrupted or continuous warfarin. J Interv Card Electrophysiol. 2017;48(3):267-282. doi:10.1007/s10840-016-0221-7 28078536

[365] Zheng GH, Yang L, Chen HY, Chu JF, Mei L. Aloe vera for prevention and treatment of infusion phlebitis. Cochrane Database Syst Rev. 2014;2014(6):CD009162. doi:10.1002/14651858.CD009162.pub2 24895299 PMC6464352

[366] Zheng MH, Sun HT, Xu JG, . Combining whole-brain radiotherapy with gefitinib/erlotinib for brain metastases from non-small-cell lung cancer: a meta-analysis. Biomed Res Int. 2016;2016:5807346. doi:10.1155/2016/5807346 27006948 PMC4783530

[367] Zheng YX, Zhou PC, Zhou RR, Fan XG. The benefit of statins in chronic hepatitis C patients: a systematic review and meta-analysis. Eur J Gastroenterol Hepatol. 2017;29(7):759-766. doi:10.1097/MEG.0000000000000867 28240613

[368] Zhou ZR, Liu SX, Zhang TS, . Short-course preoperative radiotherapy with immediate surgery versus long-course chemoradiation with delayed surgery in the treatment of rectal cancer: a systematic review and meta-analysis. Surg Oncol. 2014;23(4):211-221. doi:10.1016/j.suronc.2014.10.003 25466851

[369] Zhu LB, Liu LL, Yao L, Wang LN. Efficacy and safety of tacrolimus versus cyclophosphamide for primary membranous nephropathy: a meta-analysis. Drugs. 2017;77(2):187-199. doi:10.1007/s40265-016-0683-z 28084563

[370] Zhu RL, Chen ZJ, Li S, . Statin-treated patients with aneurysmal subarachnoid haemorrhage: a meta-analysis. Eur Rev Med Pharmacol Sci. 2016;20(10):2090-2098. 27249609

[371] Zhuang S, Wang HF, Wang X, Li J, Xing CM. The association of renin-angiotensin system blockade use with the risks of cognitive impairment of aging and Alzheimer’s disease: a meta-analysis. J Clin Neurosci. 2016;33:32-38. doi:10.1016/j.jocn.2016.02.036 27475317

[372] Ziff OJ, Lane DA, Samra M, . Safety and efficacy of digoxin: systematic review and meta-analysis of observational and controlled trial data. BMJ. 2015;351:h4451. doi:10.1136/bmj.h4451 26321114 PMC4553205

[373] Zuo SR, Zuo XC, Wang CJ, . A meta-analysis comparing the efficacy of entecavir and tenofovir for the treatment of chronic hepatitis B infection. J Clin Pharmacol. 2015;55(3):288-297. doi:10.1002/jcph.409 25293471

[374] Wood L, Egger M, Gluud LL, . Empirical evidence of bias in treatment effect estimates in controlled trials with different interventions and outcomes: meta-epidemiological study. BMJ. 2008;336(7644):601-605. doi:10.1136/bmj.39465.451748.AD 18316340 PMC2267990

[375] Lonjon G, Boutron I, Trinquart L, . Comparison of treatment effect estimates from prospective nonrandomized studies with propensity score analysis and randomized controlled trials of surgical procedures. Ann Surg. 2014;259(1):18-25. doi:10.1097/SLA.0000000000000256 24096758

[376] Kuss O, Legler T, Börgermann J. Treatments effects from randomized trials and propensity score analyses were similar in similar populations in an example from cardiac surgery. J Clin Epidemiol. 2011;64(10):1076-1084. doi:10.1016/j.jclinepi.2011.01.005 21482068

[377] Hernán MA, Wang W, Leaf DE. Target trial emulation: a framework for causal inference from observational data. JAMA. 2022;328(24):2446-2447. doi:10.1001/jama.2022.21383 36508210

[378] Janda GS, Wallach JD, Dhodapkar MM, Ramachandran R, Ross JS. Feasibility of emulating clinical trials supporting US FDA supplemental indication approvals of drugs and biologics. JAMA Intern Med. 2023;183(11):1271-1273. doi:10.1001/jamainternmed.2023.4073 37782514 PMC10546285

[379] Hansford HJ, Cashin AG, Jones MD, . Reporting of observational studies explicitly aiming to emulate randomized trials: a systematic review. JAMA Netw Open. 2023;6(9):e2336023. doi:10.1001/jamanetworkopen.2023.36023 37755828 PMC10534275

[380] Flynn R, Plueschke K, Quinten C, . Marketing authorization applications made to the European Medicines Agency in 2018-2019: what was the contribution of real-world evidence? Clin Pharmacol Ther. 2022;111(1):90-97. doi:10.1002/cpt.2461 34689339 PMC9299056

[381] Purpura CA, Garry EM, Honig N, Case A, Rassen JA. The role of real-world evidence in FDA-approved new drug and biologics license applications. Clin Pharmacol Ther. 2022;111(1):135-144. doi:10.1002/cpt.2474 34726771 PMC9299054

[382] Eskola SM, Leufkens HGM, Bate A, De Bruin ML, Gardarsdottir H. Use of real-world data and evidence in drug development of medicinal products centrally authorized in Europe in 2018-2019. Clin Pharmacol Ther. 2022;111(1):310-320. doi:10.1002/cpt.2462 34689334 PMC9299055

[383] Kaplan RM, Koong AJ, Irvin V. Review of evidence supporting 2022 US Food and Drug Administration drug approvals. JAMA Netw Open. 2023;6(8):e2327650. doi:10.1001/jamanetworkopen.2023.27650 37552481 PMC10410475

[384] Zhang AD, Puthumana J, Downing NS, Shah ND, Krumholz HM, Ross JS. Assessment of clinical trials supporting US Food and Drug Administration approval of novel therapeutic agents, 1995-2017. JAMA Netw Open. 2020;3(4):e203284. doi:10.1001/jamanetworkopen.2020.3284 32315070 PMC7175081

[385] US Food and Drug Administration. Demonstrating substantial evidence of effectiveness with one adequate and well-controlled clinical investigation and confirmatory evidence: guidance for industry. September 2023. Accessed September 27, 2023. https://www.fda.gov/media/172166/download

[386] Djurisic S, Rath A, Gaber S, . Barriers to the conduct of randomised clinical trials within all disease areas. Trials. 2017;18(1):360. doi:10.1186/s13063-017-2099-9 28764809 PMC5539637

[387] Naci H, Davis C, Savović J, . Design characteristics, risk of bias, and reporting of randomised controlled trials supporting approvals of cancer drugs by European Medicines Agency, 2014-16: cross sectional analysis. BMJ. 2019;366:l5221. doi:10.1136/bmj.l5221 31533922 PMC6749182

[388] Mehra MR, Desai SS, Ruschitzka F, Patel AN. Notice of retraction: hydroxychloroquine or chloroquine with or without a macrolide for treatment of COVID-19: a multinational registry analysis. Lancet. Published online May 22, 2020. doi:10.1016/S0140-6736(20)31180-6 32511943 PMC7274621

[389] Prasad V, Cifu A. Medical reversal: why we must raise the bar before adopting new technologies. Yale J Biol Med. 2011;84(4):471-478. 22180684 PMC3238324

[390] Herrera-Perez D, Haslam A, Crain T, . A comprehensive review of randomized clinical trials in three medical journals reveals 396 medical reversals. Elife. 2019;8:8. doi:10.7554/eLife.45183 31182188 PMC6559784

[391] Haslam A, Livingston C, Prasad V. Medical reversals in family practice: a review. Curr Ther Res Clin Exp. 2020;92:100579. doi:10.1016/j.curtheres.2020.100579 32180846 PMC7063107

[392] Prasad V, Gall V, Cifu A. The frequency of medical reversal. Arch Intern Med. 2011;171(18):1675-1676. doi:10.1001/archinternmed.2011.295 21747003

[393] Wieseler B, Neyt M, Kaiser T, Hulstaert F, Windeler J. Replacing RCTs with real world data for regulatory decision making: a self-fulfilling prophecy? BMJ. 2023;380:e073100. doi:10.1136/bmj-2022-073100 36863730

