# Supplementary material for: Treatment Effects in Randomized and Nonrandomized Studies of Pharmacological Interventions: A Meta-Analysis
Source: JAMA Netw Open. 2024 Sep 27;7(9):e2436230. doi: 10.1001/jamanetworkopen.2024.36230 (PMC11437387; doi:10.1001/jamanetworkopen.2024.36230)
Supplement: Supplement 1. — eAppendix 1. Search Strategy eAppendix 2. Extracted Data From Source Meta-Analyses eAppendix 3. Subgroup Analyses eReferences. eTable 1. Characteristics of Included Meta-Analyses eTable 2. Results for Measures of Discrepancy Between Nonrandomized Studies and RCTs eFigure. Results From Additional Subgroup Analyses for Study-Level Characteristics [file jamanetwopen-e2436230-s001.pdf]

Supplemental Online Content

Salcher-Konrad M, Nguyen M, Savović J, Higgins JPT, Naci H. Treatment effects in randomized and nonrandomized studies of pharmacological interventions: a meta-analysis. *JAMA Netw. Open*. 2024;7(9):e2436230. doi:10.1001/jamanetworkopen.2024.36230

eAppendix 1. Search Strategy

eAppendix 2. Extracted Data From Source Meta-Analyses

eAppendix 3. Subgroup Analyses

eReferences

eTable 1. Characteristics of Included Meta-Analyses

eTable 2. Results for Measures of Discrepancy Between Nonrandomized Studies and RCTs

eFigure. Results From Additional Subgroup Analyses for Study-Level Characteristics

This supplemental material has been provided by the authors to give readers additional information about their work.

© 2024 Salcher-Konrad M et al. *JAMA Network Open*

eAppendix 1. Search Strategy

The search strategy for meta-analyses including both randomized controlled trials (RCTs) and non-randomized studies (NRS) consisted of three parts:

(1) a review of existing meta-epidemiological studies comparing RCTs and NRS,
(2) a database search in MEDLINE for meta-analyses including both RCTs and NRS,
(3) a review of all systematic reviews indexed in the Cochrane Database of Systematic Reviews that included both RCTs and NRS.

Search terms used and number of records identified for each of these three parts are presented below.

Part 1: Search for previous meta-epidemiological studies (MEDLINE via PubMed)

Previous meta-epidemiological studies were identified from an umbrella Cochrane review of studies comparing treatment effects from RCTs and NRS.[1] The searches conducted by Anglemyer et al. (2014) were updated from December 2013 until October 2018 (search date: 8 October 2018) to identify additional meta-epidemiological studies published since the umbrella review was conducted. The search terms were modified to yield a manageable number of studies and focus on pharmacological interventions.

Table 1: Search strategy for meta-epidemiological studies published since December 2013 (MEDLINE via PubMed)

Search #	Search terms	Number of records
#1	Cochrane Database Syst Rev [TA] OR search[tiab] OR meta-analysis[PT] OR MEDLINE[tiab] OR PubMed[tiab] OR (systematic*[tiab] AND review*[tiab])	438,486
#2	"Observation"[mh] OR "Cohort Studies"[mh] OR "Longitudinal Studies"[mh] OR "Retrospective Studies"[mh] OR "Prospective Studies"[mh] OR observational[tiab] OR cohort*[tiab] OR crosssectional[tiab] OR crossectional[tiab] OR cross-sectional[tiab] OR cross sectional[tiab] OR longitudinal[tiab] OR causal inference*[tw] OR causality[tw] OR “instrumental variable”[tw] OR “structural model”[tw] OR practice-based[tw] OR propensity score*[tw] OR natural experiment*[tw] OR case-control[tw] OR before-after[tw] OR pre-post[tw] OR case-cohort[tw] OR case-crossover[tw] OR serial[tiab] OR nonexperimental[tiab] OR non-experimental[tiab] OR “nonrandomized”[tiab] OR “nonrandomised”[tiab] OR “non-randomised”[tiab] OR “nonrandomised”[tiab] OR “study designs”[tiab] OR “newcastle ottawa”[tiab] OR overestimat*[tiab] OR over-estimat*[tiab] OR bias[tiab] OR "are needed"[tiab] OR (evidence[tiab] AND quality[tiab])	2,873,431
#3	compara*[tiab] OR comparison*[tiab] OR contrast*[tiab] OR similar*[tiab] OR consistent*[tiab] OR inconsistent*[tiab] OR dissimilar*[tiab] OR differen*[tiab] OR concordan*[tiab] OR discordan*[tiab] OR heterogene*[tiab] OR "Research Design"[mh]	8,499,330
#4	(pharmacologic therapy[MeSH Terms]) OR drug*[Text Word] OR medicine*[Text Word])	6,339,992
#5	((((#1) AND #2) AND #3) AND #4)	21,751
#6	Limit #5 to only humans	19,761
#7	Limit #6 to December 2013-now	8,305

Part 2: Search for systematic reviews including randomized and non-randomized studies (MEDLINE via PubMed)

Part 2 of the search strategy involved searching MEDLINE (via PubMed) for systematic reviews that included both RCTs and non-randomized studies and that included meta-analysis. The search was conducted on 8 October 2018. The database was searched from March 2000 onwards (the date when the last comprehensive meta-epidemiological study last updated their search of MEDLINE using the same search strategy[2]). Compared to the search used by Ioannidis et al. (2001), search terms were added to identify reviews of pharmacological therapies (drugs) only.

© 2024 Salcher-Konrad M et al. *JAMA Network Open*

Table 2: Search strategy for systematic reviews including both randomized and non-randomized studies, published since 2000 (MEDLINE via PubMed)

Search #	Search terms	Number of records
#1	"Observational Studies as Topic"[Mesh]	3,058
#2	"Cohort Studies"[Mesh]	1,750,345
#3	"Controlled Before-After Studies"[Mesh]	330
#4	"Cross-Sectional Studies"[Mesh]	267,550
#5	"Historically Controlled Study"[Mesh]	138
#6	"Interrupted Time Series Analysis"[Mesh]	433
#7	"Case-Control Studies"[Mesh]	921,604
#8	"Non-Randomized Controlled Trials as Topic"[Mesh]	357
#9	"Propensity Score"[Mesh]	5,576
#10	#1 OR #2 OR #3 OR #4 OR #5 OR #6 OR #7 OR #8 OR #9	2,152,490
#11	"Randomized Controlled Trials as Topic"[Mesh]	118,781
#12	("Meta-Analysis as Topic"[Mesh] OR "Network Meta-Analysis"[Mesh] OR "Meta-Analysis" [Publication Type])	104,595
#13	(pharmacologic therapy[MeSH Terms]) OR drug*[Text Word] OR medicine*[Text Word])	6,339,992
#14	#10 AND #11 AND #12 AND #13	1,303
#15	Letter[ptyp] OR Editorial[ptyp] OR Comment[ptyp]	
#16	#14 NOT #15	1,266
	Limit to humans only	1,260
	Limit to hits from March 2000-now	1,156

Part 3: Search for systematic reviews including randomized and non- randomized studies (Cochrane Database of Systematic Reviews)

Part 3 involved searching the Cochrane Database of Systematic Reviews to identify systematic reviews that included both RCTs and NRS. The search was conducted on 8 October 2018.

Table 3: Search strategy for Cochrane systematic reviews including both randomized and non-randomized studies (Cochrane Database of Systematic Reviews)

Search #	Search terms	Number of records
#1	Observational:ti,ab,kw	15,169
#2	MeSH descriptor: [Observational Studies as Topic] explode all trees	58
#3	non-randomized:ti,ab,kw	2,251
#4	MeSH descriptor: [Non-Randomized Controlled Trials as Topic] explode all trees	25
#3	quasi:ti,ab,kw	5,146
#5	cohort:ti,ab,kw	42,795
#6	MeSH descriptor: [Cohort Studies as Topic] explode all trees	142,696
#7	before-after:ti,ab,kw	1,035
#8	MeSH descriptor: [Controlled Before-After Studies] explode all trees	39
#9	Cross-sectional:ti,ab,kw	13,194
#10	MeSH descriptor: [Cross-Sectional Studies] explode all trees	4,628
#11	historica*:ti,ab,kw	3,879
#12	MeSH descriptor: [Historically Controlled Study] explode all trees	21
#13	Interrupted time series:ti,ab,kw	459
#14	MeSH descriptor: [Interrupted Time Series Analysis] explode all trees	23
#15	Case-control:ti,ab,kw	10,171
#16	MeSH descriptor: [Case-Control Studies] explode all trees	15,067
#17	Match*:ti,ab,kw	34,468
#18	Propensity:ti,ab,kw	2,845
#19	MeSH descriptor: [Propensity Score] explode all trees	199
#20	#1 or #2 or #3 or #4 or #5 or #6 or #7 or #8 or #9 or #10 or #11 or #12 or #13 or #14 or #15 or #16 or #17 or #18 or #19	230,725
#21	randomized:ti,ab,kw or rct:ti,ab,kw or "randomized controlled clinical trial":ti,ab,kw or randomization:ti,ab,kw	568,926
#22	MeSH descriptor: [Randomized Controlled Trials as Topic] explode all trees	23,085

#23	#21 or #22	569,322
#24	drug*:ti,ab,kw or medicine*:ti,ab,kw	394,028
#25	MeSH descriptor: [Drug Therapy] explode all trees	138,470
#26	#24 or #25	446,327
#27	#20 and #23 and #26	56,186
	Limit to Cochrane reviews only	341

© 2024 Salcher-Konrad M et al. *JAMA Network Open*

<u>eAppendix 2.</u> Extracted Data From Source Meta-Analyses

	Variable name	Description
**Meta analysis-level information**	Source meta-analysis ID	Unique ID for each source meta-analysis (ID is identical for meta-epidemiological studies contributing more than 1 topic).
	Source meta-analysis	Name and publication year of source meta-analysis.
	Topic ("drug" and "outcome" in "patients")	Brief topic description through combination of the name of the drug, outcome, and participants.
	Therapeutic area (ATC code first level)	First-level ATC code under which the drug is categorized.
	Participants	Brief description of participants, as reported in source meta-analysis.
	Intervention	Name of the pharmacological intervention.
	Comparator	Name of the comparator.
	Outcome	Outcome of interest, as reported in source meta-analysis.
	Beneficial vs. detrimental outcome	"Beneficial" for outcomes where an increase in the number of participants experiencing an event, or an increase in the measured scale, is desired. "Detrimental" for outcomes where an increase in the number of participants experiencing an event, or an increase in the measured scale, is not desired.
	Do authors report pooled estimate within text?	Indication of whether single pooled effect estimate (obtained from both RCT and NRS in the same meta-analysis) is reported in the abstract, main body of text, or only included in a figure.
	Studies included in meta-analysis	Total number of studies with information contributing to the pooled effect.
**Study-level information**	Study name	Name of study, as reported in source meta-analysis.
	Year	Year of study, as reported in source meta-analysis.
	PMID	Unique PubMed ID for study, if available.
	Other	Unique ID for study, if not indexed in PubMed.
	Study type	RCT or non-randomized study, as reported in the source meta-analysis.<br>• RCT: study is described as RCT in source meta-analysis.<br>• NRS: study is described as non- randomized study in source meta-analysis. For Cochrane reviews: study does not use a random sequence generation.
	Study type details	Study type categorization, based on information provided in source meta-analysis.<br>• Experimental: applies to NRS only; investigators assign participants to intervention or control arm (e.g. in a non- randomized controlled trial)<br>• Observational: applies to NRS only; no experimental set-up of the study<br>• Individual RCT: applies to RCT only; randomization done at the individual level<br>• Cluster RCT: applies to RCTs only; randomization done at the group/practice/hospital/regional level<br>• Unclear: insufficient information to categorize study
	NRS type	Non- randomized study type categorization, based on information provided in source meta-analysis.<br>• Quasi- randomized: a quasi-random allocation sequence was used, such as date of birth; social insurance number; odd vs. even date; etc.<br>• Non- randomized: participants were allocated to intervention and control arm in a clinical trial based on investigator's or physician's preference (no measures taken to ensure random or quasi-random allocation).<br>• Retrospective Cohort: participants who have received an intervention are retrospectively compared to those who have not received that intervention.<br>• Prospective Cohort: participants who receive an intervention are compared to those who do not receive that intervention, and the study is planned prospectively.

		• Case-Control: retrospective studies where study participants are identified through their outcome.<br>• Controlled before-and-after<br>• Cross-sectional<br>• Retrospective analysis of RCT: only applies when randomization was not preserved<br>• Unclear/other<br>• N/A: only applies to RCTs
	NRS data source	Non- randomized study data source categorization, based on information provided in source meta-analysis.<br>• Prospectively collected data for study: data collected for the purpose of the study<br>• Disease registry<br>• Product registry<br>• Hospital case records<br>• Primary or secondary care administrative database<br>• Claims database<br>• Other/unclear data sources<br>• N/A: only applies to RCTs
	NRS data source: other	For non- randomized studies with “other” data source, the type of data source is described.
	NRS analytical method	For non- randomized studies only, the type of analytical method was extracted, as reported in the source meta-analysis, or inferred based on information provided in the source meta-analysis.
	NRS type of control	For NRS only: which type of control was used. Information either as reported in source meta-analysis or, where possible, inferred from description of study in source meta-analysis. Options include:<br>• Concurrent: Participants in intervention and control arm are recruited from the same pool of patients, so that they could conceivably have received either the novel or the standard (control) treatment.<br>• Historical: “In historical cohort studies, the treated cohort is compared with an untreated cohort who did not receive the intervention in a previous period, i.e. the individuals are not studied concurrently.” (NICE DSU Technical Support Document 17)<br>• Self-control: the control group consists of the same individuals as those receiving the experimental intervention. E.g. wound dressing (half of the wound treated with new intervention, other half with standard treatment), or comparisons within individuals over time (observation without intervention, followed by observation with intervention).<br>• Unclear: insufficient information in source meta-analysis to categorize type of control.<br>• N/A: only applies to RCTs
	Study sites	• Single-center: includes studies where information was provided on the exact center/hospital where the study took place, or reference is made in the source meta-analysis to the study being “single-center”<br>• Multi-center: includes studies where more than one country/city, or more than one center, are listed, or reference is made in the source meta-analysis to the study being “multi-center”<br>• Unclear: includes studies where reference is made to a single country or city, but no specific study site is mentioned.<br>• Not reported: no information about study sites were available in the source meta-analysis.
	Comparator type	• Active<br>• Placebo/No treatment: includes standard of care
	Outcome type	Outcomes were categorized into three types. In case of doubts, Wood et al. (*BMJ,* 2008) was used as key reference. For composite outcomes, the most subjective component was used to categorize the study as objective or subjective.<br>• Mortality<br>• Objective<br>• Subjective

© 2024 Salcher-Konrad M et al. *JAMA Network Open*

	Participant details	Details about study participants (inclusion criteria), as reported in the source meta-analysis. If no study-level information was provided, the definition for population as used in the meta-analysis was extracted.
	Intervention details (dosing, administration, treatment period)	Details about the intervention, as reported in the source meta-analysis. If no study-level information was provided, the definition for intervention as used in the meta-analysis was extracted.
	Risk of bias tool	Name of the tool that was used to assess risk of bias, as reported in the source meta-analysis.
	Risk of bias score	Overall score or overall judgement for the risk of bias tool used, as reported in the source meta-analysis.
	Risk of Bias categorization	Standardization of risk of bias scores into "high", "moderate" and "low" risk of bias.
	N total sample size	Number of participants in intervention and control arms of the study. Function of numbers of participants in each arm.
	N intervention arm	Number of participants in the intervention arm of the study, as reported in the source meta-analysis.
	N control arm	Number of participants in the control arm of the study, as reported in the source meta-analysis.
	Effect measure	Effect measure for pooled effect estimate, as reported in the source meta-analysis. Including: RR=risk ratio; OR=odds ratio; HR=hazard ratio; RD=risk difference; MD=mean difference; SMD=standardized mean difference
	Coding of effect measure	Information on how the relationship between intervention and control arm was presented in the source meta-analysis:<br>• Intervention vs. control:<br>For relative effect measures (RR, OR, HR): events in intervention arm / events in control arm<br>For absolute effect measures (RD, MD, SMD): effect in intervention arm – effect in control arm<br>• Control vs. intervention:<br>For relative effect measures (RR, OR, HR): events in control arm / events in intervention arm<br>For absolute effect measures (RD, MD, SMD): effect in control arm – effect in intervention arm
	Effect estimate as reported	Effect estimate, as reported in the source meta-analysis.
	Measure of variance	Indicates which measure of variance was reported in the source meta-analysis (including standard error, 95% confidence interval).
	SE as reported	Standard error of effect estimate, as reported in the source meta-analysis (N/A if not reported).
	Lower 95% CI as reported	Lower bound of 95% confidence interval of effect estimate, as reported in the source meta-analysis.
	Upper 95% CI as reported	Upper bound of 95% confidence interval of effect estimate, as reported in the source meta-analysis.
	p-value as reported	p-value for effect estimate, if reported in the source meta-analysis (N/A if not reported).

© 2024 Salcher-Konrad M et al. *JAMA Network Open*

<u>eAppendix 3.</u> Subgoup Analyses

Study-level characteristics were often not reported in detail in source meta-analyses, resulting in small sample sizes for most subgroups. We therefore only report the results of subgroup analyses for selected characteristics:

- Type of NRS: The discrepancy in treatment effects between experimental NRS and RCTs may be different from observational NRS, which are not typically conducted in a controlled clinical research setting.

- Type of comparator: In studies with an active comparator, the novel drug may not be clearly identified, and it may therefore be unclear in which direction bias would operate.[3] On the other hand, a clear direction of bias operating in favor of the drug can be expected in topics comparing a pharmacological intervention to placebo or no treatment.

- Type of outcome: Mortality represents the most objective outcome and is commonly deemed the most important outcome for health interventions. Studies measuring subjective outcomes may be at higher risk for bias overall,[4] and the impact of the lack of random allocation of participants may be obscured by differential reporting of subjective outcomes in RCTs vs. (observational) NRS, in which blinding would not be feasible. By stratifying the analysis according to outcome, we created more homogenous samples to focus on the impact of randomization on treatment effects.

- Matching of RCTs and NRS in a meta-analysis by PICO criteria: We assigned scores from 1 (low) to 3 (high) for how narrowly participants, intervention, comparator, and outcome were defined in each meta-analysis to give an overall score of matching quality between the two study types within each meta-analysis (range 4-12).

- Methodological quality: We restricted our sample to meta-analyses considered to be likely the most methodologically robust using two proxy indicators: meta-analyses published in the journals with the 10 top five-year impact factors as of January 2020, and meta-analyses conducted by Cochrane groups.

- Type of analysis used in NRS: Where this information was available from source meta-analyses, we identified NRS using naïve analysis (no adjustment), some form of adjustment for patient characteristics (one or more covariates included in regression models), propensity score-based analysis, and matching by individual patient characteristics to assess heterogeneity in the agreement of effect estimates according to the analytical methods used.

- Data source used in NRS: Where this information was available from source meta-analyses, we grouped NRS where data were collected specifically for the study, that used clinical RWD (disease registries and case records), and that used non-clinical, administrative RWD (administrative or claims data).

© 2024 Salcher-Konrad M et al. *JAMA Network Open*

eReferences

1. Anglemyer A, Horvath HT, Bero L. Healthcare outcomes assessed with observational study designs compared with those assessed in randomized trials. *Cochrane Database Syst Rev.* 2014(4):MR000034.
2. Ioannidis JP, Haidich AB, Pappa M, et al. Comparison of evidence of treatment effects in randomized and nonrandomized studies. *JAMA.* 2001;286(7):821-830.
3. Franklin JM, Dejene S, Huybrechts KF, Wang SV, Kulldorff M, Rothman KJ. A bias in the evaluation of bias comparing randomized trials with nonexperimental studies. *Epidemiologic Methods.* 2017;6(1).
4. Savović J, Jones HE, Altman DG, et al. Influence of reported study design characteristics on intervention effect estimates from randomized, controlled trials. *Annals of Internal Medicine.* 2012;157(6):429-438.

eTable 1. Characteristics of Included Meta-Analyses

Source meta-analysis	Reference as cited in manuscript	Comparator	Outcome type	Therapeutic area by WHO ATC code first level categorization	Risk of bias across NRSs in a meta-analysis	Risk of bias across RCTs in a meta-analysis	Median publication year of the studies included in the meta-analyses	Matching quality of RCTs and NRSs in meta-analysis	Timing of evidence generation	Cochrane review	Meta-analysis published in a top journal
Abolhassani 2013	39	Active	Other objective outcome	Anti-infective for systemic use	Moderate median risk of bias	Low median risk of bias	2000-2009	Moderate (score of 7-9 of 12)	NRS published before first RCT	No	No
Afolabi 2012	40	Active	Other objective outcome	Nervous system	High median risk of bias	Moderate median risk of bias	Before 2000	Moderate (score of 7-9 of 12)	NRS published before first RCT	Yes	Yes
Agarwal 2017	41	Placebo or no treatment	Subjective outcome	Blood and blood forming organs	Low median risk of bias	No risk of bias information	2010 and later	Moderate (score of 7-9 of 12)	NRS published before first RCT	No	No
Agarwal 2018	42	Active	Subjective outcome	Blood and blood forming organs	Low median risk of bias	Moderate median risk of bias	2000-2009	Moderate (score of 7-9 of 12)	NRS published before first RCT	No	No
Alfirevic 2009	43	Placebo or no treatment	Other objective outcome	Systemic hormonal preparations	High median risk of bias	High median risk of bias	Before 2000	Moderate (score of 7-9 of 12)	First RCT published before NRS	Yes	Yes
Allen 2010	44	Placebo or no treatment	Subjective outcome	Alimentary tract and metabolism	High median risk of bias	High median risk of bias	2000-2009	Low (score of 4-6 of 12)	First NRS and first RCT published in the same year	Yes	Yes

© 2024 Salcher-Konrad M et al. *JAMA Network Open*

Ampuero 2016	45	Placebo or no treatment	Other objective outcome	Antineoplastic and immuno-modulating agents	No risk of bias information	No risk of bias information	2010 and later	High (score of 10-12 of 12)	First NRS and first RCT published in the same year	No	No
An 2017	46	Placebo or no treatment	Other objective outcome	Cardiovascular system	Low median risk of bias	Moderate median risk of bias	2000-2009	Low (score of 4-6 of 12)	NRS published before first RCT	No	No
Andia 2014	47	Active	Subjective outcome	Blood and blood forming organs	Moderate median risk of bias	High median risk of bias	2010 and later	High (score of 10-12 of 12)	First NRS and first RCT published in the same year	No	No
Antoniou 2014	48	Placebo or no treatment	Mortality	Cardiovascular system	Low median risk of bias	Moderate median risk of bias	2000-2009	Low (score of 4-6 of 12)	First RCT published before NRS	No	No
Araujo 2015	49	Placebo or no treatment	Mortality	Antineoplastic and immuno-modulating agents	Low median risk of bias	Moderate median risk of bias	2000-2009	Moderate (score of 7-9 of 12)	First RCT published before NRS	No	No
Arnaud 2014	50	Placebo or no treatment	Subjective outcome	Blood and blood forming organs	No risk of bias information	No risk of bias information	2000-2009	High (score of 10-12 of 12)	NRS published before first RCT	No	No
Austin 2015	51	Placebo or no treatment	Other objective outcome	Alimentary tract and metabolism	High median risk of bias	Moderate median risk of bias	Before 2000	Moderate (score of 7-9 of 12)	NRS published before first RCT	Yes	Yes
Ayoub 2016	52	Placebo or no treatment	Mortality	Blood and blood forming organs	Low median risk of bias	Low median risk of bias	2010 and later	Moderate (score of 7-9 of 12)	NRS published before first RCT	No	No

© 2024 Salcher-Konrad M et al. *JAMA Network Open*

Bai 2015	53	Placebo or no treatment	Subjective outcome	Dermatologicals	High median risk of bias	Low median risk of bias	2010 and later	Moderate (score of 7-9 of 12)	First RCT published before NRS	No	No
Bakhshehian 2015	54	Placebo or no treatment	Other objective outcome	Anti-infective for systemic use	Moderate median risk of bias	Low median risk of bias	2010 and later	High (score of 10-12 of 12)	NRS published before first RCT	No	No
Baldinger 2012	55	Placebo or no treatment	Subjective outcome	Alimentary tract and metabolism	High median risk of bias	High median risk of bias	Before 2000	Moderate (score of 7-9 of 12)	First NRS and first RCT published in the same year	Yes	Yes
Ballinger 2014	56	Active	Other objective outcome	Anti-infective for systemic use	High median risk of bias	High median risk of bias	Before 2000	Moderate (score of 7-9 of 12)	NRS published before first RCT	Yes	Yes
Bang 2015	57	Placebo or no treatment	Other objective outcome	Musculo-skeletal system	Moderate median risk of bias	Low median risk of bias	2000-2009	Moderate (score of 7-9 of 12)	NRS published before first RCT	No	No
Barkat 2017	58	Placebo or no treatment	Subjective outcome	Blood and blood forming organs	Low median risk of bias	Moderate median risk of bias	2010 and later	High (score of 10-12 of 12)	First RCT published before NRS	No	No
Bellemain-Appaix	59	Placebo or no treatment	Mortality	Blood and blood forming organs	Low median risk of bias	High median risk of bias	2000-2009	Low (score of 4-6 of 12)	First NRS and first RCT published in the same year	No	Yes
Benjo 2016	60	Placebo or no treatment	Other objective outcome	Cardiovascular system	Low median risk of bias	Low median risk of bias	2000-2009	Low (score of 4-6 of 12)	NRS published before first RCT	No	No
Bhangu 2014	61	Placebo or no treatment	Other objective outcome	Musculo-skeletal system	Low median risk of bias	Low median risk of bias	2000-2009	Low (score of 4-6 of 12)	First RCT published before NRS	No	No

© 2024 Salcher-Konrad M et al. *JAMA Network Open*

Bloom 2012	62	Active	Subjective outcome	Systemic hormonal preparations	High median risk of bias	High median risk of bias	2000-2009	High (score of 10-12 of 12)	First NRS and first RCT published in the same year	Yes	Yes
Bonet 2017	63	Placebo or no treatment	Subjective outcome	Anti-infective for systemic use	High median risk of bias	High median risk of bias	Before 2000	Low (score of 4-6 of 12)	NRS published before first RCT	Yes	Yes
Bosanquet 2015	64	Placebo or no treatment	Other objective outcome	Nervous system	High median risk of bias	Moderate median risk of bias	Before 2000	Moderate (score of 7-9 of 12)	NRS published before first RCT	No	No
Bossard 2017	65	Placebo or no treatment	Other objective outcome	Blood and blood forming organs	Low median risk of bias	High median risk of bias	2000-2009	Moderate (score of 7-9 of 12)	NRS published before first RCT	No	No
Boyle 2012	66	Placebo or no treatment	Subjective outcome	Anti-infective for systemic use	High median risk of bias	High median risk of bias	2000-2009	Low (score of 4-6 of 12)	First RCT published before NRS	Yes	Yes
Branger 2016	67	Active	Other objective outcome	Antineoplastic and immuno-modulating agents	High median risk of bias	Moderate median risk of bias	2010 and later	Moderate (score of 7-9 of 12)	First NRS and first RCT published in the same year	No	Yes
Brennan 2012	68	Placebo or no treatment	Subjective outcome	Dermatologicals	High median risk of bias	Moderate median risk of bias	Before 2000	Low (score of 4-6 of 12)	First RCT published before NRS	Yes	Yes
Brito 2017	69	Placebo or no treatment	Subjective outcome	Antiparasitic products	Moderate median risk of bias	High median risk of bias	2000-2009	Moderate (score of 7-9 of 12)	First RCT published before NRS	No	Yes
Brogly 2014	70	Placebo or no treatment	Subjective outcome	Nervous system	No risk of bias information	No risk of bias information	2010 and later	Moderate (score of 7-9 of 12)	First RCT published before NRS	No	No

© 2024 Salcher-Konrad M et al. *JAMA Network Open*

Brustia 2016	71	Both active and placebo-controlled studies	Other objective outcome	Blood and blood forming organs	Moderate median risk of bias	High median risk of bias	2010 and later	Low (score of 4-6 of 12)	First RCT published before NRS	No	No
Budden 2014	72	Active	Other objective outcome	Systemic hormonal preparations	High median risk of bias	High median risk of bias	Before 2000	High (score of 10-12 of 12)	First RCT published before NRS	Yes	Yes
Caldwell 2016	73	Placebo or no treatment	Subjective outcome	Nervous system	High median risk of bias	High median risk of bias	Before 2000	Moderate (score of 7-9 of 12)	NRS published before first RCT	Yes	Yes
Campbell 2017	74	Placebo or no treatment	Other objective outcome	Anti-infective for systemic use	High median risk of bias	Moderate median risk of bias	2000-2009	Moderate (score of 7-9 of 12)	NRS published before first RCT	Yes	Yes
Carneiro 2015	75	Placebo or no treatment	Mortality	Antineoplastic and immuno-modulating agents	No risk of bias information	No risk of bias information	2000-2009	Moderate (score of 7-9 of 12)	First RCT published before NRS	No	No
Chai-Adisaksopha 2016	76	Active	Mortality	Blood and blood forming organs	Moderate median risk of bias	High median risk of bias	2010 and later	High (score of 10-12 of 12)	First RCT published before NRS	No	No
Chalhoub 2017	77	Placebo or no treatment	Subjective outcome	Antineoplastic and immuno-modulating agents	Low median risk of bias	No risk of bias information	2010 and later	Moderate (score of 7-9 of 12)	First RCT published before NRS	No	No
Chao 2017	78	Placebo or no treatment	Subjective outcome	Genito-urinary system and sex hormones	Low median risk of bias	Moderate median risk of bias	2010 and later	Moderate (score of 7-9 of 12)	First NRS and first RCT published in the same year	No	No

Chen 2013	79	Placebo or no treatment	Other objective outcome	Anti-infective for systemic use	No risk of bias information	No risk of bias information	2000-2009	Moderate (score of 7-9 of 12)	NRS published before first RCT	No	Yes
Chen 2015	80	Placebo or no treatment	Subjective outcome	Blood and blood forming organs	No risk of bias information	No risk of bias information	2010 and later	High (score of 10-12 of 12)	NRS published before first RCT	No	No
Chen 2015	81	Placebo or no treatment	Other objective outcome	Anti-infective for systemic use	High median risk of bias	Moderate median risk of bias	2010 and later	Moderate (score of 7-9 of 12)	First RCT published before NRS	No	No
Chen 2017	82	Placebo or no treatment	Other objective outcome	Antineoplastic and immuno-modulating agents	Low median risk of bias	High median risk of bias	2010 and later	Moderate (score of 7-9 of 12)	NRS published before first RCT	No	No
Cheng 2015	83	Placebo or no treatment	Subjective outcome	Sensory organs	No risk of bias information	Low median risk of bias	2010 and later	Moderate (score of 7-9 of 12)	First RCT published before NRS	No	No
Chowdhury 2018	84	Placebo or no treatment	Mortality	Dermatologicals	High median risk of bias	Moderate median risk of bias	Before 2000	High (score of 10-12 of 12)	First RCT published before NRS	No	No
Chrcanovic 2014	85	Placebo or no treatment	Other objective outcome	Anti-infective for systemic use	High median risk of bias	Low median risk of bias	2000-2009	Low (score of 4-6 of 12)	First RCT published before NRS	No	No
Clifton 2014	86	Placebo or no treatment	Subjective outcome	Alimentary tract and metabolism	No risk of bias information	No risk of bias information	2010 and later	Moderate (score of 7-9 of 12)	First RCT published before NRS	No	No
Coppola 2015	87	Placebo or no treatment	Other objective outcome	Blood and blood forming organs	High median risk of bias	Moderate median risk of bias	Before 2000	High (score of 10-12 of 12)	NRS published before first RCT	Yes	Yes

© 2024 Salcher-Konrad M et al. *JAMA Network Open*

Costi 2014	88	Placebo or no treatment	Subjective outcome	Nervous system	High median risk of bias	Moderate median risk of bias	Before 2000	High (score of 10-12 of 12)	First RCT published before NRS	Yes	Yes
Coussement 2018	89	Placebo or no treatment	Other objective outcome	Anti-infective for systemic use	High median risk of bias	High median risk of bias	2010 and later	Moderate (score of 7-9 of 12)	NRS published before first RCT	Yes	Yes
Critchley 2014	90	Placebo or no treatment	Mortality	Systemic hormonal preparations	High median risk of bias	Moderate median risk of bias	Before 2000	Low (score of 4-6 of 12)	First RCT published before NRS	Yes	Yes
Cui 2014	91	Both active and placebo-controlled studies	Other objective outcome	Blood and blood forming organs	No risk of bias information	No risk of bias information	2000-2009	Moderate (score of 7-9 of 12)	NRS published before first RCT	No	No
Dahal 2015	92	Placebo or no treatment	Other objective outcome	Cardiovascular system	No risk of bias information	Low median risk of bias	2010 and later	High (score of 10-12 of 12)	First RCT published before NRS	No	No
David 2017	93	Active	Subjective outcome	Systemic hormonal preparations	High median risk of bias	High median risk of bias	2010 and later	Moderate (score of 7-9 of 12)	First RCT published before NRS	Yes	Yes
de Frutos 2015	94	Placebo or no treatment	Other objective outcome	Cardiovascular system	High median risk of bias	Moderate median risk of bias	Before 2000	Moderate (score of 7-9 of 12)	NRS published before first RCT	No	No
Desiderio 2017	95	Placebo or no treatment	Mortality	Antineoplastic and immuno-modulating agents	Low median risk of bias	Moderate median risk of bias	2000-2009	Low (score of 4-6 of 12)	NRS published before first RCT	No	No
Di 2015	96	Active	Other objective outcome	Anti-infective for systemic use	Low median risk of bias	Low median risk of bias	2000-2009	Moderate (score of 7-9 of 12)	First RCT published before NRS	No	No

© 2024 Salcher-Konrad M et al. *JAMA Network Open*

Dong 2017	97	Active	Other objective outcome	Alimentary tract and metabolism	Low median risk of bias	Moderate median risk of bias	2010 and later	Moderate (score of 7-9 of 12)	First NRS and first RCT published in the same year	No	No
Edmonds 2012	98	Placebo or no treatment	Other objective outcome	Respiratory system	High median risk of bias	Moderate median risk of bias	2000-2009	Moderate (score of 7-9 of 12)	First RCT published before NRS	Yes	Yes
El Sayed 2018	99	Active	Other objective outcome	Anti-infective for systemic use	High median risk of bias	High median risk of bias	2000-2009	Moderate (score of 7-9 of 12)	First RCT published before NRS	Yes	Yes
Elgendy 2017	100	Active	Subjective outcome	Blood and blood forming organs	Low median risk of bias	Low median risk of bias	2010 and later	Moderate (score of 7-9 of 12)	NRS published before first RCT	No	No
Engelen 2018	101	Placebo or no treatment	Subjective outcome	Blood and blood forming organs	High median risk of bias	Moderate median risk of bias	Before 2000	High (score of 10-12 of 12)	First RCT published before NRS	Yes	Yes
Engelman 2010	102	Placebo or no treatment	Other objective outcome	Systemic hormonal preparations	High median risk of bias	High median risk of bias	2000-2009	Moderate (score of 7-9 of 12)	First RCT published before NRS	No	No
Estcourt 2015	103	Placebo or no treatment	Mortality	Antineoplastic and immuno-modulating agents	High median risk of bias	High median risk of bias	Before 2000	Moderate (score of 7-9 of 12)	First RCT published before NRS	Yes	Yes
Facciorusso 2018	104	Both active and placebo-controlled studies	Mortality	Cardiovascular system	Low median risk of bias	High median risk of bias	2010 and later	Low (score of 4-6 of 12)	First NRS and first RCT published in the same year	No	No

© 2024 Salcher-Konrad M et al. *JAMA Network Open*

Falagas 2013	105	Active	Other objective outcome	Anti-infective for systemic use	No risk of bias information	No risk of bias information	2000-2009	High (score of 10-12 of 12)	First RCT published before NRS	No	Yes
Feng 2015	106	Both active and placebo-controlled studies	Other objective outcome	Alimentary tract and metabolism	No risk of bias information	No risk of bias information	2010 and later	High (score of 10-12 of 12)	First RCT published before NRS	No	No
Ferrer 2016	107	Placebo or no treatment	Other objective outcome	Anti-infective for systemic use	No risk of bias information	No risk of bias information	2000-2009	Low (score of 4-6 of 12)	First NRS and first RCT published in the same year	No	No
Filippini 2017	108	Placebo or no treatment	Subjective outcome	Antineoplastic and immuno-modulating agents	High median risk of bias	High median risk of bias	2010 and later	High (score of 10-12 of 12)	First RCT published before NRS	Yes	Yes
Fukuta 2017	109	Placebo or no treatment	Mortality	Cardiovascular system	Low median risk of bias	Low median risk of bias	2000-2009	Low (score of 4-6 of 12)	First RCT published before NRS	No	No
Fung 2015	110	Active	Other objective outcome	Anti-infective for systemic use	Low median risk of bias	Moderate median risk of bias	2010 and later	Low (score of 4-6 of 12)	First RCT published before NRS	No	Yes
Furtado 2014	111	Placebo or no treatment	Subjective outcome	Various	No risk of bias information	Low median risk of bias	2000-2009	High (score of 10-12 of 12)	First RCT published before NRS	No	No
Galappaththy 2013	112	Placebo or no treatment	Other objective outcome	Antiparasitic products	High median risk of bias	Moderate median risk of bias	2000-2009	Moderate (score of 7-9 of 12)	First RCT published before NRS	Yes	Yes

Gandhi 2015	113	Placebo or no treatment	Subjective outcome	Blood and blood forming organs	Moderate median risk of bias	High median risk of bias	2010 and later	High (score of 10-12 of 12)	First RCT published before NRS	No	No
Gausden 2017	114	Placebo or no treatment	Other objective outcome	Blood and blood forming organs	No risk of bias information	No risk of bias information	2010 and later	Moderate (score of 7-9 of 12)	First RCT published before NRS	No	No
Gharaibeh 2013	115	Placebo or no treatment	Subjective outcome	Blood and blood forming organs	High median risk of bias	High median risk of bias	Before 2000	High (score of 10-12 of 12)	First RCT published before NRS	Yes	Yes
Gillespie 2010	116	Placebo or no treatment	Subjective outcome	Anti-infective for systemic use	High median risk of bias	High median risk of bias	Before 2000	Moderate (score of 7-9 of 12)	First RCT published before NRS	Yes	Yes
Gong 2017	117	Placebo or no treatment	Subjective outcome	Sensory organs	Low median risk of bias	Moderate median risk of bias	2000-2009	Moderate (score of 7-9 of 12)	NRS published before first RCT	No	Yes
Gonzalez 2018	118	Active	Other objective outcome	Antiparasitic products	High median risk of bias	Low median risk of bias	2010 and later	High (score of 10-12 of 12)	NRS published before first RCT	Yes	Yes
Grabein 2017	119	Active	Subjective outcome	Anti-infective for systemic use	High median risk of bias	Moderate median risk of bias	Before 2000	Moderate (score of 7-9 of 12)	First RCT published before NRS	No	No
Graves 2010	120	Placebo or no treatment	Other objective outcome	Anti-infective for systemic use	No risk of bias information	No risk of bias information	Before 2000	High (score of 10-12 of 12)	NRS published before first RCT	Yes	Yes
Graves 2018	121	Placebo or no treatment	Other objective outcome	Antiparasitic products	High median risk of bias	High median risk of bias	2010 and later	Moderate (score of 7-9 of 12)	First RCT published before NRS	Yes	Yes

Gray 2016	122	Placebo or no treatment	Mortality	Cardiovascular system	Low median risk of bias	Moderate median risk of bias	2010 and later	Low (score of 4-6 of 12)	NRS published before first RCT	No	No
Guerra 2017	123	Both active and placebo-controlled studies	Subjective outcome	Cardiovascular system	High median risk of bias	Low median risk of bias	2010 and later	Moderate (score of 7-9 of 12)	NRS published before first RCT	No	No
Gunter 2017	124	Both active and placebo-controlled studies	Subjective outcome	Musculo-skeletal system	No risk of bias information	No risk of bias information	2000-2009	Moderate (score of 7-9 of 12)	First RCT published before NRS	No	No
Haas 2015	125	Active	Mortality	Dermatologicals	High median risk of bias	High median risk of bias	Before 2000	High (score of 10-12 of 12)	First RCT published before NRS	Yes	Yes
Han 2016	126	Active	Other objective outcome	Blood and blood forming organs	No risk of bias information	No risk of bias information	2010 and later	High (score of 10-12 of 12)	First NRS and first RCT published in the same year	No	No
Han 2017	127	Active	Other objective outcome	Anti-infective for systemic use	Low median risk of bias	Moderate median risk of bias	2010 and later	High (score of 10-12 of 12)	First RCT published before NRS	No	No
Hannah 2016	128	Active	Other objective outcome	Dermatologicals	Moderate median risk of bias	High median risk of bias	2010 and later	High (score of 10-12 of 12)	NRS published before first RCT	No	No
Hao 2016	129	Active	Other objective outcome	Anti-infective for systemic use	Moderate median risk of bias	Moderate median risk of bias	2000-2009	High (score of 10-12 of 12)	NRS published before first RCT	No	No

Harnoss 2017	130	Placebo or no treatment	Subjective outcome	Anti-infective for systemic use	High median risk of bias	High median risk of bias	2010 and later	Moderate (score of 7-9 of 12)	First RCT published before NRS	No	No
Haroon 2014	131	Placebo or no treatment	Other objective outcome	Antineoplastic and immuno-modulating agents	Moderate median risk of bias	No risk of bias information	2010 and later	Moderate (score of 7-9 of 12)	NRS published before first RCT	No	No
Hass 2018	132	Placebo or no treatment	Subjective outcome	Genito-urinary system and sex hormones	High median risk of bias	High median risk of bias	2010 and later	Moderate (score of 7-9 of 12)	First RCT published before NRS	Yes	Yes
He 2013	133	Active	Other objective outcome	Antineoplastic and immuno-modulating agents	Low median risk of bias	Low median risk of bias	2010 and later	High (score of 10-12 of 12)	First RCT published before NRS	No	Yes
He 2015	134	Active	Subjective outcome	Cardiovascular system	Low median risk of bias	Low median risk of bias	2000-2009	Low (score of 4-6 of 12)	First RCT published before NRS	No	No
Heal 2017	135	Placebo or no treatment	Other objective outcome	Dermatologicals	High median risk of bias	Low median risk of bias	2000-2009	Moderate (score of 7-9 of 12)	NRS published before first RCT	No	No
Hemkens 2016	14	Placebo or no treatment	Mortality	Cardiovascular system	Moderate median risk of bias	High median risk of bias	2000-2009	Moderate (score of 7-9 of 12)	First NRS and first RCT published in the same year	No	Yes
Hemkens 2016	14	Placebo or no treatment	Mortality	Blood and blood forming organs	Moderate median risk of bias	High median risk of bias	2010 and later	Moderate (score of 7-9 of 12)	NRS published before first RCT	No	Yes

© 2024 Salcher-Konrad M et al. *JAMA Network Open*

Hemkens 2016	14	Placebo or no treatment	Mortality	Blood and blood forming organs	Moderate median risk of bias	Moderate median risk of bias	2010 and later	Moderate (score of 7-9 of 12)	NRS published before first RCT	No	Yes
Henderson-Smart 2010	136	Placebo or no treatment	Subjective outcome	Respiratory system	Moderate median risk of bias	High median risk of bias	Before 2000	Moderate (score of 7-9 of 12)	First NRS and first RCT published in the same year	Yes	Yes
Henssler 2016	137	Placebo or no treatment	Subjective outcome	Nervous system	High median risk of bias	High median risk of bias	2000-2009	Moderate (score of 7-9 of 12)	First RCT published before NRS	No	No
Hernandez 2017	138	Active	Other objective outcome	Anti-infective for systemic use	Low median risk of bias	High median risk of bias	Before 2000	High (score of 10-12 of 12)	First NRS and first RCT published in the same year	No	No
Hodson 2013	139	Placebo or no treatment	Subjective outcome	Anti-infective for systemic use	High median risk of bias	High median risk of bias	Before 2000	Moderate (score of 7-9 of 12)	First RCT published before NRS	Yes	Yes
Hong 2014	140	Placebo or no treatment	Other objective outcome	Antineoplastic and immuno-modulating agents	Low median risk of bias	Low median risk of bias	2010 and later	Moderate (score of 7-9 of 12)	First RCT published before NRS	No	No
Horbach 2016	141	Active	Other objective outcome	Antineoplastic and immuno-modulating agents	Moderate median risk of bias	Moderate median risk of bias	2010 and later	Moderate (score of 7-9 of 12)	NRS published before first RCT	No	No
Horita 2016	142	Placebo or no treatment	Mortality	Anti-infective for systemic use	Low median risk of bias	High median risk of bias	2000-2009	Moderate (score of 7-9 of 12)	NRS published before first RCT	No	No

© 2024 Salcher-Konrad M et al. *JAMA Network Open*

Hu 2014	143	Placebo or no treatment	Other objective outcome	Musculo-skeletal system	No risk of bias information	No risk of bias information	2000-2009	Low (score of 4-6 of 12)	NRS published before first RCT	No	No
Hu 2015	144	Active	Mortality	Respiratory system	No risk of bias information	No risk of bias information	2000-2009	Moderate (score of 7-9 of 12)	NRS published before first RCT	No	No
Hu 2016	145	Placebo or no treatment	Other objective outcome	Antineoplastic and immuno-modulating agents	No risk of bias information	No risk of bias information	2010 and later	Moderate (score of 7-9 of 12)	First RCT published before NRS	No	No
Huang 2013	146	Placebo or no treatment	Mortality	Cardiovascular system	High median risk of bias	Low median risk of bias	2000-2009	High (score of 10-12 of 12)	First RCT published before NRS	No	No
Huang 2016	147	Placebo or no treatment	Subjective outcome	Blood and blood forming organs	Moderate median risk of bias	High median risk of bias	2010 and later	Low (score of 4-6 of 12)	NRS published before first RCT	No	No
Huang 2017	148	Active	Subjective outcome	Anti-infective for systemic use	Low median risk of bias	Low median risk of bias	2010 and later	Moderate (score of 7-9 of 12)	First RCT published before NRS	No	Yes
Huang 2017	149	Placebo or no treatment	Other objective outcome	Genito-urinary system and sex hormones	High median risk of bias	High median risk of bias	2010 and later	Moderate (score of 7-9 of 12)	First RCT published before NRS	No	Yes
Hughes 2016	150	Placebo or no treatment	Subjective outcome	Systemic hormonal preparations	High median risk of bias	High median risk of bias	Before 2000	Low (score of 4-6 of 12)	First RCT published before NRS	Yes	Yes
Hunt 2010	151	Placebo or no treatment	Mortality	Blood and blood forming organs	High median risk of bias	Moderate median risk of bias	Before 2000	High (score of 10-12 of 12)	First RCT published before NRS	Yes	Yes

Hyun 2017	152	Placebo or no treatment	Other objective outcome	Anti-infective for systemic use	Low median risk of bias	High median risk of bias	2010 and later	High (score of 10-12 of 12)	NRS published before first RCT	No	No
Jain 2016	153	Active	Subjective outcome	Nervous system	High median risk of bias	High median risk of bias	2000-2009	High (score of 10-12 of 12)	First RCT published before NRS	No	No
Ji 2017	154	Placebo or no treatment	Other objective outcome	Antineoplastic and immuno-modulating agents	No risk of bias information	No risk of bias information	2010 and later	Moderate (score of 7-9 of 12)	First RCT published before NRS	No	No
Jiang 2015	155	Placebo or no treatment	Other objective outcome	Genito-urinary system and sex hormones	Moderate median risk of bias	Moderate median risk of bias	2000-2009	High (score of 10-12 of 12)	First RCT published before NRS	No	No
Jiang 2016	156	Placebo or no treatment	Other objective outcome	Blood and blood forming organs	Low median risk of bias	Moderate median risk of bias	2010 and later	High (score of 10-12 of 12)	First NRS and first RCT published in the same year	No	No
Jian-Yu 2018	157	Placebo or no treatment	Mortality	Alimentary tract and metabolism	No risk of bias information	No risk of bias information	2010 and later	Moderate (score of 7-9 of 12)	NRS published before first RCT	No	No
Johnston 2017	158	Active	Mortality	Anti-infective for systemic use	Moderate median risk of bias	Low median risk of bias	2010 and later	High (score of 10-12 of 12)	NRS published before first RCT	No	No
Kabra 2013	159	Placebo or no treatment	Subjective outcome	Anti-infective for systemic use	High median risk of bias	High median risk of bias	Before 2000	Low (score of 4-6 of 12)	NRS published before first RCT	Yes	Yes

© 2024 Salcher-Konrad M et al. *JAMA Network Open*

Kalil 2009	160	Active	Other objective outcome	Anti-infective for systemic use	No risk of bias information	Moderate median risk of bias	2000-2009	Moderate (score of 7-9 of 12)	NRS published before first RCT	No	Yes
Kamal 2017	161	Both active and placebo-controlled studies	Subjective outcome	Alimentary tract and metabolism	Moderate median risk of bias	High median risk of bias	2010 and later	Moderate (score of 7-9 of 12)	NRS published before first RCT	No	No
Kamal 2017	162	Placebo or no treatment	Mortality	Cardiovascular system	Low median risk of bias	Low median risk of bias	2010 and later	Low (score of 4-6 of 12)	NRS published before first RCT	No	No
Kanbay 2014	163	Placebo or no treatment	Other objective outcome	Musculo-skeletal system	High median risk of bias	High median risk of bias	2010 and later	Moderate (score of 7-9 of 12)	First RCT published before NRS	No	No
Kaplan 2016	164	Placebo or no treatment	Different types of outcomes	Antiparasitic products	Low median risk of bias	Moderate median risk of bias	2000-2009	Moderate (score of 7-9 of 12)	NRS published before first RCT	No	No
Kenyon 2013	165	Active	Other objective outcome	Systemic hormonal preparations	High median risk of bias	High median risk of bias	2000-2009	Moderate (score of 7-9 of 12)	First RCT published before NRS	Yes	Yes
Kessel 2015	166	Both active and placebo-controlled studies	Different types of outcomes	Anti-infective for systemic use	High median risk of bias	High median risk of bias	2010 and later	Low (score of 4-6 of 12)	NRS published before first RCT	No	No
Khan 2016	167	Active	Subjective outcome	Nervous system	Low median risk of bias	High median risk of bias	2010 and later	High (score of 10-12 of 12)	NRS published before first RCT	No	No
Khan 2017	168	Placebo or no treatment	Subjective outcome	Dermatologicals	Low median risk of bias	Low median risk of bias	2010 and later	Moderate (score of 7-9 of 12)	NRS published before first RCT	No	No

© 2024 Salcher-Konrad M et al. *JAMA Network Open*

Khan 2017	169	Placebo or no treatment	Mortality	Cardiovascular system	Moderate median risk of bias	Low median risk of bias	2000-2009	Low (score of 4-6 of 12)	NRS published before first RCT	No	No
Khoshbin 2013	170	Both active and placebo-controlled studies	Subjective outcome	Blood and blood forming organs	Low median risk of bias	Low median risk of bias	2010 and later	Moderate (score of 7-9 of 12)	First RCT published before NRS	No	No
Kim 2016	171	Active	Other objective outcome	Respiratory system	Low median risk of bias	Moderate median risk of bias	2010 and later	Moderate (score of 7-9 of 12)	First RCT published before NRS	No	Yes
Kirkland 2017	172	Placebo or no treatment	Other objective outcome	Respiratory system	High median risk of bias	No risk of bias information	2000-2009	Moderate (score of 7-9 of 12)	First RCT published before NRS	Yes	Yes
Kirsch 2017	173	Placebo or no treatment	Other objective outcome	Blood and blood forming organs	Low median risk of bias	Moderate median risk of bias	2010 and later	Moderate (score of 7-9 of 12)	First RCT published before NRS	No	No
Kitsios 2015	174	Placebo or no treatment	Mortality	Blood and blood forming organs	No risk of bias information	Moderate median risk of bias	2000-2009	Moderate (score of 7-9 of 12)	First RCT published before NRS	No	No
Kitsios 2015	174	Active	Mortality	Blood and blood forming organs	No risk of bias information	Moderate median risk of bias	2010 and later	Moderate (score of 7-9 of 12)	First RCT published before NRS	No	No
Kitsios 2015	174	Both active and placebo-controlled studies	Mortality	Blood and blood forming organs	No risk of bias information	High median risk of bias	2000-2009	High (score of 10-12 of 12)	First RCT published before NRS	No	No
Kitsios 2015	174	Placebo or no treatment	Mortality	Cardiovascular system	No risk of bias information	High median risk of bias	2000-2009	Moderate (score of 7-9 of 12)	First RCT published before NRS	No	No

© 2024 Salcher-Konrad M et al. *JAMA Network Open*

Kitsios 2015	174	Placebo or no treatment	Mortality	Cardiovascular system	No risk of bias information	Low median risk of bias	2010 and later	Moderate (score of 7-9 of 12)	NRS published before first RCT	No	No
Kitsios 2015	174	Placebo or no treatment	Mortality	Cardiovascular system	No risk of bias information	Low median risk of bias	2010 and later	Moderate (score of 7-9 of 12)	First RCT published before NRS	No	No
Kitsios 2015	174	Placebo or no treatment	Mortality	Systemic hormonal preparations	No risk of bias information	Moderate median risk of bias	2000-2009	Moderate (score of 7-9 of 12)	First RCT published before NRS	No	No
Kitsios 2015	174	Both active and placebo-controlled studies	Mortality	Blood and blood forming organs	No risk of bias information	Moderate median risk of bias	2000-2009	Moderate (score of 7-9 of 12)	First RCT published before NRS	No	No
Kitsios 2015	174	Both active and placebo-controlled studies	Mortality	Blood and blood forming organs	No risk of bias information	Moderate median risk of bias	2000-2009	Moderate (score of 7-9 of 12)	First RCT published before NRS	No	No
Klimo 2014	175	Placebo or no treatment	Other objective outcome	Anti-infective for systemic use	Moderate median risk of bias	Low median risk of bias	Before 2000	Low (score of 4-6 of 12)	NRS published before first RCT	No	No
Kovacs 2016	176	Placebo or no treatment	Other objective outcome	Antiparasitic products	Moderate median risk of bias	High median risk of bias	2010 and later	Moderate (score of 7-9 of 12)	First RCT published before NRS	No	Yes
Kowalewski 2016	177	Active	Subjective outcome	Blood and blood forming organs	Low median risk of bias	Moderate median risk of bias	2000-2009	High (score of 10-12 of 12)	First RCT published before NRS	No	No
Krajewski	178	Active	Mortality	Blood and blood forming organs	Low median risk of bias	Moderate median risk of bias	2010 and later	High (score of 10-12 of 12)	NRS published before first RCT	No	No

© 2024 Salcher-Konrad M et al. *JAMA Network Open*

Kroon 2015	179	Active	Subjective outcome	Musculo-skeletal system	High median risk of bias	Moderate median risk of bias	Before 2000	Moderate (score of 7-9 of 12)	First RCT published before NRS	Yes	Yes
Kuang 2017	180	Placebo or no treatment	Subjective outcome	Nervous system	Moderate median risk of bias	Low median risk of bias	2010 and later	High (score of 10-12 of 12)	First RCT published before NRS	No	No
Kwok 2013	181	Placebo or no treatment	Subjective outcome	Alimentary tract and metabolism	No risk of bias information	Low median risk of bias	2010 and later	High (score of 10-12 of 12)	NRS published before first RCT	No	No
Lee 2017	182	Active	Other objective outcome	Anti-infective for systemic use	Low median risk of bias	Moderate median risk of bias	2010 and later	Moderate (score of 7-9 of 12)	First RCT published before NRS	No	No
Lee 2017	183	Active	Mortality	Anti-infective for systemic use	High median risk of bias	High median risk of bias	2000-2009	Moderate (score of 7-9 of 12)	First RCT published before NRS	No	No
Leibovici-Weissman 2014	184	Placebo or no treatment	Subjective outcome	Anti-infective for systemic use	High median risk of bias	High median risk of bias	Before 2000	Low (score of 4-6 of 12)	NRS published before first RCT	Yes	Yes
Lemos 2014	185	Active	Subjective outcome	Antineoplastic and immuno-modulating agents	Low median risk of bias	High median risk of bias	2010 and later	High (score of 10-12 of 12)	First NRS and first RCT published in the same year	No	No
Leone 2016	186	Placebo or no treatment	Subjective outcome	Nervous system	High median risk of bias	High median risk of bias	Before 2000	Moderate (score of 7-9 of 12)	First RCT published before NRS	Yes	Yes
Lewis 2018	187	Both active and placebo-controlled studies	Mortality	Blood and blood forming organs	High median risk of bias	Moderate median risk of bias	2010 and later	Low (score of 4-6 of 12)	First RCT published before NRS	Yes	Yes

© 2024 Salcher-Konrad M et al. *JAMA Network Open*

Li 2015	188	Placebo or no treatment	Other objective outcome	Antineoplastic and immuno-modulating agents	Moderate median risk of bias	Moderate median risk of bias	2010 and later	High (score of 10-12 of 12)	First RCT published before NRS	No	No
Li 2015	189	Placebo or no treatment	Other objective outcome	Sensory organs	Low median risk of bias	Low median risk of bias	2000-2009	Moderate (score of 7-9 of 12)	First RCT published before NRS	No	Yes
Li 2016	190	Placebo or no treatment	Subjective outcome	Blood and blood forming organs	No risk of bias information	No risk of bias information	2010 and later	Moderate (score of 7-9 of 12)	First RCT published before NRS	No	No
Li 2017	191	Placebo or no treatment	Subjective outcome	Musculo-skeletal system	Low median risk of bias	Low median risk of bias	2010 and later	High (score of 10-12 of 12)	First RCT published before NRS	No	No
Li 2018	192	Active	Subjective outcome	Antineoplastic and immuno-modulating agents	No risk of bias information	No risk of bias information	2010 and later	High (score of 10-12 of 12)	First RCT published before NRS	No	No
Liang 2014	193	Both active and placebo-controlled studies	Mortality	Antineoplastic and immuno-modulating agents	Moderate median risk of bias	Low median risk of bias	2000-2009	Moderate (score of 7-9 of 12)	NRS published before first RCT	No	No
Liang 2017	194	Placebo or no treatment	Subjective outcome	Nervous system	Low median risk of bias	Moderate median risk of bias	2010 and later	High (score of 10-12 of 12)	First RCT published before NRS	No	No
Liet 2015	195	Placebo or no treatment	Other objective outcome	Various	High median risk of bias	Moderate median risk of bias	2000-2009	High (score of 10-12 of 12)	First RCT published before NRS	Yes	Yes

© 2024 Salcher-Konrad M et al. *JAMA Network Open*

Lim 2015	196	Placebo or no treatment	Other objective outcome	Anti-infective for systemic use	Low median risk of bias	Moderate median risk of bias	2000-2009	Moderate (score of 7-9 of 12)	First RCT published before NRS	No	No
Lim 2015	197	Placebo or no treatment	Other objective outcome	Cardiovascular system	No risk of bias information	Moderate median risk of bias	2010 and later	High (score of 10-12 of 12)	First RCT published before NRS	No	No
Lin 2015	198	Placebo or no treatment	Mortality	Antineoplastic and immuno-modulating agents	High median risk of bias	Low median risk of bias	2000-2009	Low (score of 4-6 of 12)	NRS published before first RCT	No	No
Liu 2013	199	Placebo or no treatment	Other objective outcome	Musculo-skeletal system	Low median risk of bias	Low median risk of bias	2010 and later	Low (score of 4-6 of 12)	NRS published before first RCT	No	No
Liu 2014	200	Placebo or no treatment	Subjective outcome	Anti-infective for systemic use	Moderate median risk of bias	Low median risk of bias	2010 and later	Moderate (score of 7-9 of 12)	NRS published before first RCT	No	Yes
Liu 2015	201	Placebo or no treatment	Subjective outcome	Blood and blood forming organs	Moderate median risk of bias	High median risk of bias	2000-2009	High (score of 10-12 of 12)	First RCT published before NRS	No	Yes
Liu 2016	202	Placebo or no treatment	Other objective outcome	Dermatologicals	Low median risk of bias	Low median risk of bias	2000-2009	Moderate (score of 7-9 of 12)	First RCT published before NRS	No	No
Liu 2017	203	Placebo or no treatment	Other objective outcome	Anti-infective for systemic use	Moderate median risk of bias	Moderate median risk of bias	2000-2009	High (score of 10-12 of 12)	First RCT published before NRS	No	Yes
Loomba 2015	204	Placebo or no treatment	Subjective outcome	Cardiovascular system	Low median risk of bias	No risk of bias information	2010 and later	High (score of 10-12 of 12)	NRS published before first RCT	No	No

Lu 2014	205	Placebo or no treatment	Other objective outcome	Anti-infective for systemic use	Low median risk of bias	Moderate median risk of bias	2010 and later	Moderate (score of 7-9 of 12)	NRS published before first RCT	No	No
Luni 2018	206	Placebo or no treatment	Other objective outcome	Cardiovascular system	Low median risk of bias	High median risk of bias	2010 and later	Moderate (score of 7-9 of 12)	NRS published before first RCT	No	No
Lussana 2014	207	Active	Subjective outcome	Blood and blood forming organs	No risk of bias information	No risk of bias information	2010 and later	High (score of 10-12 of 12)	First RCT published before NRS	No	No
Ma 2015	208	Placebo or no treatment	Subjective outcome	Blood and blood forming organs	High median risk of bias	High median risk of bias	2000-2009	Low (score of 4-6 of 12)	First RCT published before NRS	No	Yes
Mackeen 2011	209	Placebo or no treatment	Mortality	Genito-urinary system and sex hormones	High median risk of bias	High median risk of bias	Before 2000	Moderate (score of 7-9 of 12)	First RCT published before NRS	Yes	Yes
Mao 2015	210	Active	Other objective outcome	Systemic hormonal preparations	No risk of bias information	No risk of bias information	2000-2009	Low (score of 4-6 of 12)	First RCT published before NRS	No	No
Matthews 2016	211	Active	Other objective outcome	Systemic hormonal preparations	High median risk of bias	High median risk of bias	2000-2009	Moderate (score of 7-9 of 12)	First RCT published before NRS	Yes	Yes
Mbeye 2014	212	Placebo or no treatment	Other objective outcome	Anti-infective for systemic use	No risk of bias information	No risk of bias information	2010 and later	Moderate (score of 7-9 of 12)	First RCT published before NRS	No	No
Meduri 2016	213	Placebo or no treatment	Subjective outcome	Nervous system	Moderate median risk of bias	Moderate median risk of bias	2000-2009	Moderate (score of 7-9 of 12)	NRS published before first RCT	No	No

© 2024 Salcher-Konrad M et al. *JAMA Network Open*

Merlotti 2014	214	Placebo or no treatment	Other objective outcome	Cardiovascular system	High median risk of bias	High median risk of bias	2000-2009	Low (score of 4-6 of 12)	First RCT published before NRS	No	No
Mesfin 2016	215	Placebo or no treatment	Other objective outcome	Anti-infective for systemic use	Moderate median risk of bias	Moderate median risk of bias	2000-2009	Moderate (score of 7-9 of 12)	NRS published before first RCT	No	No
Mesgarpour 2017	216	Placebo or no treatment	Subjective outcome	Blood and blood forming organs	Low median risk of bias	Moderate median risk of bias	2010 and later	Low (score of 4-6 of 12)	First RCT published before NRS	Yes	Yes
Miyake 2010	217	Placebo or no treatment	Other objective outcome	Antineoplastic and immuno-modulating agents	No risk of bias information	No risk of bias information	2000-2009	Low (score of 4-6 of 12)	NRS published before first RCT	No	No
Moraes 2014	218	Placebo or no treatment	Subjective outcome	Blood and blood forming organs	High median risk of bias	High median risk of bias	2000-2009	Moderate (score of 7-9 of 12)	NRS published before first RCT	Yes	Yes
Muanda 2015	219	Placebo or no treatment	Other objective outcome	Antiparasitic products	High median risk of bias	Moderate median risk of bias	2000-2009	Low (score of 4-6 of 12)	First RCT published before NRS	No	Yes
Munnee 2016	220	Placebo or no treatment	Mortality	Alimentary tract and metabolism	No risk of bias information	Moderate median risk of bias	2000-2009	Moderate (score of 7-9 of 12)	NRS published before first RCT	No	No
Muranushi 2015	221	Placebo or no treatment	Other objective outcome	Blood and blood forming organs	Low median risk of bias	Low median risk of bias	2010 and later	Low (score of 4-6 of 12)	NRS published before first RCT	No	No
Muranushi 2015	222	Placebo or no treatment	Other objective outcome	Blood and blood forming organs	High median risk of bias	Low median risk of bias	2010 and later	Low (score of 4-6 of 12)	NRS published before first RCT	No	No

© 2024 Salcher-Konrad M et al. *JAMA Network Open*

Murphy 2016	223	Placebo or no treatment	Other objective outcome	Anti-infective for systemic use	High median risk of bias	Moderate median risk of bias	Before 2000	Low (score of 4-6 of 12)	NRS published before first RCT	No	No
Muzii 2016	224	Active	Subjective outcome	Genito-urinary system and sex hormones	No risk of bias information	Moderate median risk of bias	2010 and later	Moderate (score of 7-9 of 12)	First RCT published before NRS	No	No
Nairooz 2017	225	Placebo or no treatment	Subjective outcome	Blood and blood forming organs	Low median risk of bias	Low median risk of bias	2000-2009	High (score of 10-12 of 12)	First NRS and first RCT published in the same year	No	No
Neufeld 2016	226	Placebo or no treatment	Subjective outcome	Nervous system	High median risk of bias	High median risk of bias	2010 and later	Low (score of 4-6 of 12)	First RCT published before NRS	No	No
Niafar 2015	227	Placebo or no treatment	Other objective outcome	Alimentary tract and metabolism	Low median risk of bias	Low median risk of bias	2000-2009	Moderate (score of 7-9 of 12)	First RCT published before NRS	No	No
Nie 2016	228	Placebo or no treatment	Mortality	Antineoplastic and immuno-modulating agents	Low median risk of bias	Moderate median risk of bias	2010 and later	High (score of 10-12 of 12)	NRS published before first RCT	No	No
O'Brien 2014	229	Placebo or no treatment	Subjective outcome	Genito-urinary system and sex hormones	No risk of bias information	No risk of bias information	2000-2009	Low (score of 4-6 of 12)	NRS published before first RCT	No	No
Ogunlesi 2015	230	Placebo or no treatment	Mortality	Systemic hormonal preparations	High median risk of bias	High median risk of bias	2000-2009	Moderate (score of 7-9 of 12)	NRS published before first RCT	Yes	Yes

© 2024 Salcher-Konrad M et al. *JAMA Network Open*

Ohlsson 2015	231	Placebo or no treatment	Mortality	Anti-infective for systemic use	High median risk of bias	High median risk of bias	Before 2000	Moderate (score of 7-9 of 12)	NRS published before first RCT	Yes	Yes
Okoli 2014	232	Placebo or no treatment	Subjective outcome	Anti-infective for systemic use	Moderate median risk of bias	No risk of bias information	2000-2009	Moderate (score of 7-9 of 12)	First RCT published before NRS	No	Yes
Ortize-Orendain 2017	233	Placebo or no treatment	Subjective outcome	Nervous system	High median risk of bias	High median risk of bias	2000-2009	Moderate (score of 7-9 of 12)	First RCT published before NRS	Yes	Yes
Ortiz-Salas 2016	234	Active	Subjective outcome	Anti-infective for systemic use	Low median risk of bias	Low median risk of bias	2000-2009	Moderate (score of 7-9 of 12)	First RCT published before NRS	No	No
Osborn 2010	235	Active	Subjective outcome	Nervous system	High median risk of bias	High median risk of bias	Before 2000	High (score of 10-12 of 12)	First RCT published before NRS	Yes	Yes
Osborn 2010	236	Active	Subjective outcome	Nervous system	High median risk of bias	High median risk of bias	Before 2000	High (score of 10-12 of 12)	First RCT published before NRS	Yes	Yes
Paciaroni 2011	237	Both active and placebo-controlled studies	Subjective outcome	Blood and blood forming organs	No risk of bias information	No risk of bias information	2000-2009	Moderate (score of 7-9 of 12)	First RCT published before NRS	No	No
Pammi 2015	238	Placebo or no treatment	Mortality	Cardiovascular system	High median risk of bias	High median risk of bias	2000-2009	High (score of 10-12 of 12)	First RCT published before NRS	Yes	Yes
Pan 2015	239	Placebo or no treatment	Subjective outcome	Blood and blood forming organs	No risk of bias information	No risk of bias information	2010 and later	Low (score of 4-6 of 12)	NRS published before first RCT	No	No

© 2024 Salcher-Konrad M et al. *JAMA Network Open*

Pan 2016	240	Both active and placebo-controlled studies	Other objective outcome	Nervous system	Moderate median risk of bias	Low median risk of bias	2010 and later	Moderate (score of 7-9 of 12)	First RCT published before NRS	No	No
Pani 2014	241	Placebo or no treatment	Subjective outcome	Nervous system	High median risk of bias	High median risk of bias	2010 and later	Low (score of 4-6 of 12)	First RCT published before NRS	Yes	Yes
Paul 2014	242	Active	Mortality	Anti-infective for systemic use	High median risk of bias	High median risk of bias	Before 2000	Moderate (score of 7-9 of 12)	First RCT published before NRS	Yes	Yes
Paul 2016	243	Active	Mortality	Anti-infective for systemic use	High median risk of bias	Moderate median risk of bias	2010 and later	High (score of 10-12 of 12)	NRS published before first RCT	No	No
Paul 2016	244	Placebo or no treatment	Other objective outcome	Anti-infective for systemic use	Low median risk of bias	Moderate median risk of bias	2010 and later	Low (score of 4-6 of 12)	NRS published before first RCT	No	Yes
Perez-Gaxiola 2018	245	Placebo or no treatment	Subjective outcome	Alimentary tract and metabolism	High median risk of bias	Moderate median risk of bias	2000-2009	Moderate (score of 7-9 of 12)	First RCT published before NRS	Yes	Yes
Peters 2014	246	Both active and placebo-controlled studies	Subjective outcome	Cardiovascular system	No risk of bias information	No risk of bias information	2000-2009	Low (score of 4-6 of 12)	NRS published before first RCT	No	No
Prasad 2014	247	Placebo or no treatment	Subjective outcome	Nervous system	High median risk of bias	Low median risk of bias	Before 2000	Moderate (score of 7-9 of 12)	First RCT published before NRS	Yes	Yes

© 2024 Salcher-Konrad M et al. *JAMA Network Open*

Price 2012	248	Active	Subjective outcome	Genito-urinary system and sex hormones	High median risk of bias	Moderate median risk of bias	Before 2000	High (score of 10-12 of 12)	NRS published before first RCT	Yes	Yes
Prijic 2014	249	Both active and placebo-controlled studies	Subjective outcome	Cardiovascular system	No risk of bias information	No risk of bias information	2000-2009	Moderate (score of 7-9 of 12)	First RCT published before NRS	No	No
Prins 2015	250	Active	Mortality	Cardiovascular system	High median risk of bias	High median risk of bias	2000-2009	High (score of 10-12 of 12)	NRS published before first RCT	No	No
Proietti 2015	251	Active	Subjective outcome	Blood and blood forming organs	Low median risk of bias	Moderate median risk of bias	2000-2009	Moderate (score of 7-9 of 12)	First RCT published before NRS	No	No
Prutsky 2013	252	Placebo or no treatment	Subjective outcome	Anti-infective for systemic use	Moderate median risk of bias	Moderate median risk of bias	Before 2000	Low (score of 4-6 of 12)	NRS published before first RCT	No	No
Puig 2016	253	Active	Other objective outcome	Alimentary tract and metabolism	Moderate median risk of bias	Moderate median risk of bias	2000-2009	Moderate (score of 7-9 of 12)	First RCT published before NRS	No	No
Qin 2016	254	Placebo or no treatment	Mortality	Antineoplastic and immuno-modulating agents	Low median risk of bias	Moderate median risk of bias	2000-2009	Low (score of 4-6 of 12)	First RCT published before NRS	No	No
Qiu 2017	255	Placebo or no treatment	Other objective outcome	Antineoplastic and immuno-modulating agents	Moderate median risk of bias	Low median risk of bias	2000-2009	Low (score of 4-6 of 12)	NRS published before first RCT	No	No

© 2024 Salcher-Konrad M et al. *JAMA Network Open*

Qiu 2018	256	Placebo or no treatment	Other objective outcome	Alimentary tract and metabolism	Low median risk of bias	Low median risk of bias	2000-2009	Moderate (score of 7-9 of 12)	NRS published before first RCT	No	No
Radeva-Petrova 2014	257	Placebo or no treatment	Other objective outcome	Antiparasitic products	High median risk of bias	Moderate median risk of bias	Before 2000	Moderate (score of 7-9 of 12)	NRS published before first RCT	Yes	Yes
Rivero 2017	258	Placebo or no treatment	Subjective outcome	Respiratory system	Low median risk of bias	Low median risk of bias	2010 and later	High (score of 10-12 of 12)	NRS published before first RCT	No	No
Roberts 2018	259	Active	Other objective outcome	Anti-infective for systemic use	Moderate median risk of bias	Low median risk of bias	2010 and later	Moderate (score of 7-9 of 12)	NRS published before first RCT	No	No
Rodriguez-Zuniga 2018	260	Placebo or no treatment	Subjective outcome	Anti-infective for systemic use	High median risk of bias	Moderate median risk of bias	2010 and later	High (score of 10-12 of 12)	NRS published before first RCT	No	No
Rojas-Villarraga 2014	261	Placebo or no treatment	Subjective outcome	Genito-urinary system and sex hormones	High median risk of bias	Moderate median risk of bias	2000-2009	Low (score of 4-6 of 12)	NRS published before first RCT	No	Yes
Rokkas 2014	262	Placebo or no treatment	Other objective outcome	Alimentary tract and metabolism	No risk of bias information	No risk of bias information	2010 and later	Moderate (score of 7-9 of 12)	NRS published before first RCT	No	No
Rys 2018	263	Active	Other objective outcome	Alimentary tract and metabolism	No risk of bias information	No risk of bias information	2010 and later	High (score of 10-12 of 12)	NRS published before first RCT	No	No
Sahebkar 2017	264	Placebo or no treatment	Other objective outcome	Cardiovascular system	Moderate median risk of bias	Moderate median risk of bias	2010 and later	Low (score of 4-6 of 12)	NRS published before first RCT	No	No

© 2024 Salcher-Konrad M et al. *JAMA Network Open*

Sahebkar 2017	265	Placebo or no treatment	Other objective outcome	Antineoplastic and immuno-modulating agents	Low median risk of bias	Moderate median risk of bias	Before 2000	Low (score of 4-6 of 12)	First RCT published before NRS	No	No
Salata 2018	266	Placebo or no treatment	Other objective outcome	Cardiovascular system	Low median risk of bias	Low median risk of bias	2010 and later	Low (score of 4-6 of 12)	NRS published before first RCT	No	No
Salvi 2017	267	Placebo or no treatment	Other objective outcome	Nervous system	Low median risk of bias	Low median risk of bias	2010 and later	Low (score of 4-6 of 12)	NRS published before first RCT	No	Yes
Sant'anna 2014	268	Active	Subjective outcome	Blood and blood forming organs	No risk of bias information	No risk of bias information	2010 and later	High (score of 10-12 of 12)	First NRS and first RCT published in the same year	No	No
Sardar 2014	269	Active	Subjective outcome	Blood and blood forming organs	Moderate median risk of bias	No risk of bias information	2010 and later	High (score of 10-12 of 12)	First NRS and first RCT published in the same year	No	No
Serpa Neto 2014	270	Placebo or no treatment	Other objective outcome	Blood and blood forming organs	No risk of bias information	Low median risk of bias	2010 and later	High (score of 10-12 of 12)	First RCT published before NRS	No	No
Seth 2014	271	Active	Subjective outcome	Musculo-skeletal system	High median risk of bias	High median risk of bias	2000-2009	High (score of 10-12 of 12)	First RCT published before NRS	Yes	Yes
Shang 2011	272	Active	Other objective outcome	Antineoplastic and immuno-modulating agents	High median risk of bias	High median risk of bias	Before 2000	High (score of 10-12 of 12)	NRS published before first RCT	Yes	Yes

Sharma 2014	273	Placebo or no treatment	Subjective outcome	Musculo-skeletal system	Moderate median risk of bias	No risk of bias information	2000-2009	Low (score of 4-6 of 12)	First RCT published before NRS	No	No
Shen 2016	274	Placebo or no treatment	Other objective outcome	Cardiovascular system	Low median risk of bias	Low median risk of bias	2010 and later	Low (score of 4-6 of 12)	NRS published before first RCT	No	No
Shi 2014	275	Placebo or no treatment	Other objective outcome	Cardiovascular system	Low median risk of bias	No risk of bias information	2010 and later	Low (score of 4-6 of 12)	NRS published before first RCT	No	Yes
Shi 2017	276	Placebo or no treatment	Subjective outcome	Blood and blood forming organs	High median risk of bias	High median risk of bias	2010 and later	High (score of 10-12 of 12)	NRS published before first RCT	No	No
Shim 2014	277	Placebo or no treatment	Other objective outcome	Genito-urinary system and sex hormones	Low median risk of bias	Low median risk of bias	Before 2000	Moderate (score of 7-9 of 12)	NRS published before first RCT	No	No
Shin 2015	278	Active	Subjective outcome	Antineoplastic and immuno-modulating agents	Low median risk of bias	Low median risk of bias	2010 and later	Moderate (score of 7-9 of 12)	First NRS and first RCT published in the same year	No	No
Sim 2010	279	Both active and placebo-controlled studies	Other objective outcome	Genito-urinary system and sex hormones	Moderate median risk of bias	High median risk of bias	2000-2009	High (score of 10-12 of 12)	First RCT published before NRS	No	No
Sinclair 2011	280	Placebo or no treatment	Other objective outcome	Anti-infective for systemic use	High median risk of bias	Moderate median risk of bias	Before 2000	Moderate (score of 7-9 of 12)	First RCT published before NRS	Yes	Yes

Singh 2017	281	Placebo or no treatment	Subjective outcome	Blood and blood forming organs	Moderate median risk of bias	Moderate median risk of bias	2010 and later	Moderate (score of 7-9 of 12)	First RCT published before NRS	No	No
Singh 2017	282	Placebo or no treatment	Other objective outcome	Nervous system	High median risk of bias	High median risk of bias	2010 and later	Moderate (score of 7-9 of 12)	NRS published before first RCT	No	No
Smaill 2014	283	Placebo or no treatment	Other objective outcome	Anti-infective for systemic use	High median risk of bias	Moderate median risk of bias	Before 2000	Moderate (score of 7-9 of 12)	First RCT published before NRS	Yes	Yes
Smit 2013	284	Placebo or no treatment	Subjective outcome	Nervous system	High median risk of bias	High median risk of bias	Before 2000	Moderate (score of 7-9 of 12)	First RCT published before NRS	Yes	Yes
Sole-Lleonart 2017	285	Active	Other objective outcome	Anti-infective for systemic use	Low median risk of bias	Low median risk of bias	2010 and later	High (score of 10-12 of 12)	NRS published before first RCT	No	No
Song 2017	286	Placebo or no treatment	Other objective outcome	Cardiovascular system	No risk of bias information	No risk of bias information	2010 and later	Moderate (score of 7-9 of 12)	NRS published before first RCT	No	No
Sotiriadis 2015	287	Placebo or no treatment	Subjective outcome	Systemic hormonal preparations	High median risk of bias	Low median risk of bias	2000-2009	Moderate (score of 7-9 of 12)	First RCT published before NRS	No	No
Squizzato 2010	288	Placebo or no treatment	Subjective outcome	Cardiovascular system	High median risk of bias	Low median risk of bias	2000-2009	Low (score of 4-6 of 12)	NRS published before first RCT	No	No
Stern 2014	289	Active	Subjective outcome	Anti-infective for systemic use	High median risk of bias	High median risk of bias	Before 2000	High (score of 10-12 of 12)	NRS published before first RCT	Yes	Yes

Strohmeier 2014	290	Active	Other objective outcome	Anti-infective for systemic use	High median risk of bias	High median risk of bias	2000-2009	High (score of 10-12 of 12)	First RCT published before NRS	Yes	Yes
Suthar 2015	291	Placebo or no treatment	Mortality	Anti-infective for systemic use	Low median risk of bias	Low median risk of bias	2000-2009	Moderate (score of 7-9 of 12)	First RCT published before NRS	No	Yes
Talukdar 2015	292	Placebo or no treatment	Subjective outcome	Various	High median risk of bias	Moderate median risk of bias	2000-2009	High (score of 10-12 of 12)	First RCT published before NRS	No	No
Tang 2009	293	Placebo or no treatment	Mortality	Systemic hormonal preparations	No risk of bias information	No risk of bias information	2000-2009	Moderate (score of 7-9 of 12)	First NRS and first RCT published in the same year	No	No
Tang 2016	294	Placebo or no treatment	Other objective outcome	Cardiovascular system	Low median risk of bias	High median risk of bias	2010 and later	Low (score of 4-6 of 12)	NRS published before first RCT	No	No
Tarantini 2017	295	Active	Subjective outcome	Blood and blood forming organs	Low median risk of bias	Low median risk of bias	2010 and later	High (score of 10-12 of 12)	First RCT published before NRS	No	No
Taylor 2015	296	Placebo or no treatment	Other objective outcome	Anti-infective for systemic use	High median risk of bias	Moderate median risk of bias	2000-2009	Moderate (score of 7-9 of 12)	First RCT published before NRS	Yes	Yes
Teng 2017	297	Placebo or no treatment	Other objective outcome	Cardiovascular system	Moderate median risk of bias	Moderate median risk of bias	2010 and later	High (score of 10-12 of 12)	NRS published before first RCT	No	No
Toews 2018	298	Active	Different types of outcomes	Alimentary tract and metabolism	High median risk of bias	High median risk of bias	Before 2000	High (score of 10-12 of 12)	NRS published before first RCT	Yes	Yes

Tran-Duy 2016	299	Placebo or no treatment	Subjective outcome	Alimentary tract and metabolism	Moderate median risk of bias	Moderate median risk of bias	2000-2009	Low (score of 4-6 of 12)	NRS published before first RCT	No	No
Tsai 2018	300	Active	Subjective outcome	Dermatologicals	Moderate median risk of bias	Low median risk of bias	2010 and later	High (score of 10-12 of 12)	First RCT published before NRS	No	No
Tsaousi 2016	301	Placebo or no treatment	Other objective outcome	Nervous system	High median risk of bias	Low median risk of bias	2010 and later	Moderate (score of 7-9 of 12)	NRS published before first RCT	No	No
Tully 2016	302	Placebo or no treatment	Subjective outcome	Cardiovascular system	Low median risk of bias	Low median risk of bias	2000-2009	Low (score of 4-6 of 12)	First RCT published before NRS	No	No
Tunnicliffe 2018	303	Active	Other objective outcome	Antineoplastic and immuno-modulating agents	High median risk of bias	High median risk of bias	2000-2009	High (score of 10-12 of 12)	First RCT published before NRS	Yes	Yes
Turgeon 2015	304	Placebo or no treatment	Mortality	Blood and blood forming organs	Moderate median risk of bias	High median risk of bias	2010 and later	Moderate (score of 7-9 of 12)	First RCT published before NRS	No	No
Ukaigwe 2017	305	Active	Subjective outcome	Blood and blood forming organs	Moderate median risk of bias	No risk of bias information	2010 and later	High (score of 10-12 of 12)	First NRS and first RCT published in the same year	No	No
van Herwaarden 2014	306	Active	Other objective outcome	Antineoplastic and immuno-modulating agents	High median risk of bias	High median risk of bias	2010 and later	High (score of 10-12 of 12)	First RCT published before NRS	Yes	Yes

© 2024 Salcher-Konrad M et al. *JAMA Network Open*

Vardakas 2018	307	Placebo or no treatment	Mortality	Anti-infective for systemic use	No risk of bias information	Moderate median risk of bias	2010 and later	High (score of 10-12 of 12)	NRS published before first RCT	No	No
Vecchio 2015	308	Placebo or no treatment	Other objective outcome	Systemic hormonal preparations	High median risk of bias	High median risk of bias	Before 2000	Moderate (score of 7-9 of 12)	First RCT published before NRS	Yes	Yes
Vyas 2015	309	Active	Subjective outcome	Blood and blood forming organs	Low median risk of bias	Low median risk of bias	2010 and later	High (score of 10-12 of 12)	First RCT published before NRS	No	No
Wan 2018	310	Placebo or no treatment	Subjective outcome	Cardiovascular system	Moderate median risk of bias	Low median risk of bias	2010 and later	Moderate (score of 7-9 of 12)	NRS published before first RCT	No	No
Wang 2014	311	Both active and placebo-controlled studies	Subjective outcome	Cardiovascular system	No risk of bias information	No risk of bias information	2000-2009	High (score of 10-12 of 12)	NRS published before first RCT	No	Yes
Wang 2015	312	Placebo or no treatment	Subjective outcome	Blood and blood forming organs	Moderate median risk of bias	Low median risk of bias	2000-2009	Moderate (score of 7-9 of 12)	First NRS and first RCT published in the same year	No	No
Wang 2016	313	Placebo or no treatment	Subjective outcome	Musculo-skeletal system	Low median risk of bias	Moderate median risk of bias	2010 and later	Moderate (score of 7-9 of 12)	First RCT published before NRS	No	No
Wang 2016	314	Placebo or no treatment	Other objective outcome	Anti-infective for systemic use	High median risk of bias	Moderate median risk of bias	2000-2009	Moderate (score of 7-9 of 12)	NRS published before first RCT	No	No
Wang 2017	315	Both active and placebo-	Other objective outcome	Alimentary tract and metabolism	No risk of bias information	High median risk of bias	2000-2009	Moderate (score of 7-9 of 12)	First RCT published before NRS	No	No

© 2024 Salcher-Konrad M et al. *JAMA Network Open*

		controlled studies									
Wang 2017	316	Placebo or no treatment	Other objective outcome	Antiparasitic products	Low median risk of bias	Low median risk of bias	2010 and later	Moderate (score of 7-9 of 12)	NRS published before first RCT	No	Yes
Wang 2017	317	Placebo or no treatment	Other objective outcome	Systemic hormonal preparations	Moderate median risk of bias	Low median risk of bias	2000-2009	Moderate (score of 7-9 of 12)	NRS published before first RCT	No	No
Wang 2018	318	Placebo or no treatment	Other objective outcome	Blood and blood forming organs	Low median risk of bias	Moderate median risk of bias	2010 and later	Low (score of 4-6 of 12)	First NRS and first RCT published in the same year	No	No
Watti 2017	319	Active	Mortality	Blood and blood forming organs	Low median risk of bias	No risk of bias information	2010 and later	High (score of 10-12 of 12)	First RCT published before NRS	No	No
Westhoff 2013	320	Both active and placebo-controlled studies	Subjective outcome	Systemic hormonal preparations	High median risk of bias	High median risk of bias	Before 2000	High (score of 10-12 of 12)	First RCT published before NRS	Yes	Yes
Whiting 2017	321	Active	Subjective outcome	Cardiovascular system	High median risk of bias	Moderate median risk of bias	2010 and later	High (score of 10-12 of 12)	First RCT published before NRS	No	Yes
Widmer 2015	322	Active	Other objective outcome	Anti-infective for systemic use	High median risk of bias	High median risk of bias	Before 2000	High (score of 10-12 of 12)	NRS published before first RCT	Yes	Yes
Wilhelmus 2015	323	Placebo or no treatment	Other objective outcome	Sensory organs	High median risk of bias	Moderate median risk of bias	Before 2000	Moderate (score of 7-9 of 12)	First NRS and first RCT published in the same year	Yes	Yes

© 2024 Salcher-Konrad M et al. *JAMA Network Open*

Wiysonge 2017	324	Placebo or no treatment	Other objective outcome	Systemic hormonal preparations	High median risk of bias	Moderate median risk of bias	2000-2009	Low (score of 4-6 of 12)	NRS published before first RCT	Yes	Yes
Wu 2015	325	Both active and placebo-controlled studies	Other objective outcome	Alimentary tract and metabolism	Low median risk of bias	High median risk of bias	2000-2009	Low (score of 4-6 of 12)	First RCT published before NRS	No	No
Wu 2015	326	Placebo or no treatment	Other objective outcome	Cardiovascular system	Moderate median risk of bias	Moderate median risk of bias	2010 and later	Moderate (score of 7-9 of 12)	First RCT published before NRS	No	Yes
Xia 2015	327	Placebo or no treatment	Mortality	Anti-infective for systemic use	Low median risk of bias	Low median risk of bias	2010 and later	Moderate (score of 7-9 of 12)	NRS published before first RCT	No	No
Xia 2015	328	Placebo or no treatment	Subjective outcome	Antineoplastic and immuno-modulating agents	Low median risk of bias	High median risk of bias	2010 and later	Moderate (score of 7-9 of 12)	First RCT published before NRS	No	No
Xie 2017	329	Placebo or no treatment	Other objective outcome	Antineoplastic and immuno-modulating agents	Moderate median risk of bias	High median risk of bias	2010 and later	Moderate (score of 7-9 of 12)	NRS published before first RCT	No	No
Xing 2016	330	Placebo or no treatment	Other objective outcome	Cardiovascular system	No risk of bias information	Moderate median risk of bias	2010 and later	Moderate (score of 7-9 of 12)	First RCT published before NRS	No	No
Xiong 2014	331	Active	Other objective outcome	Antineoplastic and immuno-modulating agents	Low median risk of bias	Low median risk of bias	2010 and later	Moderate (score of 7-9 of 12)	First NRS and first RCT published in the same year	No	Yes

Xu 2016	332	Both active and placebo-controlled studies	Other objective outcome	Anti-infective for systemic use	No risk of bias information	No risk of bias information	2000-2009	Moderate (score of 7-9 of 12)	First RCT published before NRS	No	No
Yang 2016	333	Active	Other objective outcome	Anti-infective for systemic use	Low median risk of bias	Moderate median risk of bias	2010 and later	High (score of 10-12 of 12)	NRS published before first RCT	No	No
Yang 2016	334	Active	Subjective outcome	Blood and blood forming organs	High median risk of bias	High median risk of bias	2000-2009	High (score of 10-12 of 12)	First RCT published before NRS	No	Yes
Yang 2016	335	Placebo or no treatment	Other objective outcome	Anti-infective for systemic use	Moderate median risk of bias	High median risk of bias	2000-2009	Moderate (score of 7-9 of 12)	NRS published before first RCT	No	Yes
Yang 2016	336	Active	Subjective outcome	Anti-infective for systemic use	Low median risk of bias	High median risk of bias	2000-2009	High (score of 10-12 of 12)	NRS published before first RCT	No	No
Yang 2017	337	Active	Other objective outcome	Antineoplastic and immuno-modulating agents	High median risk of bias	Low median risk of bias	2010 and later	High (score of 10-12 of 12)	NRS published before first RCT	No	No
Yao 2016	338	Placebo or no treatment	Subjective outcome	Nervous system	No risk of bias information	No risk of bias information	2010 and later	Moderate (score of 7-9 of 12)	NRS published before first RCT	No	No
Ye 2015	339	Placebo or no treatment	Subjective outcome	Cardiovascular system	No risk of bias information	No risk of bias information	2010 and later	Low (score of 4-6 of 12)	First RCT published before NRS	No	No

© 2024 Salcher-Konrad M et al. *JAMA Network Open*

Yin 2016	340	Active	Other objective outcome	Antineoplastic and immuno-modulating agents	Low median risk of bias	Low median risk of bias	2010 and later	High (score of 10-12 of 12)	NRS published before first RCT	No	No
Yong 2015	341	Active	Other objective outcome	Antineoplastic and immuno-modulating agents	Low median risk of bias	Low median risk of bias	2010 and later	High (score of 10-12 of 12)	NRS published before first RCT	No	Yes
Yong 2017	342	Active	Subjective outcome	Blood and blood forming organs	No risk of bias information	No risk of bias information	2010 and later	High (score of 10-12 of 12)	NRS published before first RCT	No	Yes
Yuan 2014	343	Active	Subjective outcome	Nervous system	No risk of bias information	No risk of bias information	2000-2009	Moderate (score of 7-9 of 12)	NRS published before first RCT	No	No
Zaiem 2017	344	Placebo or no treatment	Other objective outcome	Genito-urinary system and sex hormones	High median risk of bias	High median risk of bias	2010 and later	Moderate (score of 7-9 of 12)	NRS published before first RCT	No	No
Zeng 2016	345	Active	Other objective outcome	Alimentary tract and metabolism	No risk of bias information	Low median risk of bias	2000-2009	High (score of 10-12 of 12)	NRS published before first RCT	No	No
Zeng 2017	346	Placebo or no treatment	Mortality	Blood and blood forming organs	High median risk of bias	Low median risk of bias	Before 2000	Moderate (score of 7-9 of 12)	NRS published before first RCT	No	No
Zhai 2016	347	Placebo or no treatment	Subjective outcome	Nervous system	Low median risk of bias	Low median risk of bias	2000-2009	High (score of 10-12 of 12)	First NRS and first RCT published in the same year	No	No

© 2024 Salcher-Konrad M et al. *JAMA Network Open*

Zhang 2014	348	Active	Mortality	Antineoplastic and immuno-modulating agents	High median risk of bias	High median risk of bias	Before 2000	High (score of 10-12 of 12)	First RCT published before NRS	No	No
Zhang 2014	349	Placebo or no treatment	Other objective outcome	Antineoplastic and immuno-modulating agents	High median risk of bias	High median risk of bias	2000-2009	High (score of 10-12 of 12)	First RCT published before NRS	Yes	Yes
Zhang 2014	350	Placebo or no treatment	Subjective outcome	Nervous system	High median risk of bias	High median risk of bias	2010 and later	Low (score of 4-6 of 12)	NRS published before first RCT	No	No
Zhang 2016	351	Placebo or no treatment	Other objective outcome	Genito-urinary system and sex hormones	Moderate median risk of bias	No risk of bias information	2010 and later	High (score of 10-12 of 12)	NRS published before first RCT	No	No
Zhang 2017	352	Placebo or no treatment	Other objective outcome	Antineoplastic and immuno-modulating agents	Moderate median risk of bias	No risk of bias information	2000-2009	High (score of 10-12 of 12)	First NRS and first RCT published in the same year	No	No
Zhang 2017	353	Active	Other objective outcome	Respiratory system	High median risk of bias	Low median risk of bias	2010 and later	High (score of 10-12 of 12)	First RCT published before NRS	Yes	Yes
Zhang 2017	354	Active	Other objective outcome	Blood and blood forming organs	Moderate median risk of bias	Low median risk of bias	2010 and later	Moderate (score of 7-9 of 12)	First RCT published before NRS	No	No
Zhang 2017	355	Active	Other objective outcome	Blood and blood forming organs	Low median risk of bias	Low median risk of bias	2010 and later	High (score of 10-12 of 12)	First RCT published before NRS	No	No

© 2024 Salcher-Konrad M et al. *JAMA Network Open*

Zhang 2017	356	Both active and placebo-controlled studies	Subjective outcome	Systemic hormonal preparations	No risk of bias information	No risk of bias information	2000-2009	Moderate (score of 7-9 of 12)	NRS published before first RCT	No	No
Zhang 2017	357	Both active and placebo-controlled studies	Other objective outcome	Alimentary tract and metabolism	Low median risk of bias	Low median risk of bias	2010 and later	Low (score of 4-6 of 12)	First RCT published before NRS	No	Yes
Zhao 2015	358	Placebo or no treatment	Other objective outcome	Musculo-skeletal system	Low median risk of bias	Low median risk of bias	2000-2009	High (score of 10-12 of 12)	NRS published before first RCT	No	No
Zhao 2015	359	Placebo or no treatment	Other objective outcome	Genito-urinary system and sex hormones	High median risk of bias	High median risk of bias	2000-2009	High (score of 10-12 of 12)	NRS published before first RCT	No	No
Zhao 2016	360	Placebo or no treatment	Subjective outcome	Alimentary tract and metabolism	High median risk of bias	Low median risk of bias	2000-2009	Moderate (score of 7-9 of 12)	First RCT published before NRS	No	Yes
Zhao 2016	361	Placebo or no treatment	Other objective outcome	Cardiovascular system	No risk of bias information	No risk of bias information	2000-2009	Moderate (score of 7-9 of 12)	NRS published before first RCT	No	No
Zhao 2017	362	Placebo or no treatment	Mortality	Antineoplastic and immuno-modulating agents	Low median risk of bias	High median risk of bias	2000-2009	Low (score of 4-6 of 12)	First RCT published before NRS	No	No
Zhao 2017	363	Placebo or no treatment	Other objective outcome	Blood and blood forming organs	Low median risk of bias	Low median risk of bias	2010 and later	Moderate (score of 7-9 of 12)	First NRS and first RCT published in the same year	No	No

Zhao 2018	364	Placebo or no treatment	Other objective outcome	Antineoplastic and immuno-modulating agents	Moderate median risk of bias	Low median risk of bias	2010 and later	High (score of 10-12 of 12)	NRS published before first RCT	No	No
Zheng 2014	365	Active	Subjective outcome	Dermatologicals	High median risk of bias	Moderate median risk of bias	2000-2009	High (score of 10-12 of 12)	NRS published before first RCT	Yes	Yes
Zheng 2016	366	Placebo or no treatment	Other objective outcome	Antineoplastic and immuno-modulating agents	High median risk of bias	No risk of bias information	2010 and later	Moderate (score of 7-9 of 12)	First NRS and first RCT published in the same year	No	No
Zheng 2017	367	Placebo or no treatment	Other objective outcome	Cardiovascular system	Moderate median risk of bias	Low median risk of bias	2010 and later	Moderate (score of 7-9 of 12)	NRS published before first RCT	No	No
Zhou 2014	368	Active	Mortality	Antineoplastic and immuno-modulating agents	Low median risk of bias	Low median risk of bias	2010 and later	High (score of 10-12 of 12)	First RCT published before NRS	No	No
Zhu 2016	369	Placebo or no treatment	Other objective outcome	Cardiovascular system	Moderate median risk of bias	Low median risk of bias	2000-2009	Moderate (score of 7-9 of 12)	First RCT published before NRS	No	No
Zhu 2017	370	Active	Other objective outcome	Antineoplastic and immuno-modulating agents	High median risk of bias	High median risk of bias	2010 and later	High (score of 10-12 of 12)	NRS published before first RCT	No	No
Zhuang 2016	371	Placebo or no treatment	Subjective outcome	Cardiovascular system	Low median risk of bias	Low median risk of bias	2010 and later	Low (score of 4-6 of 12)	NRS published before first RCT	No	No

© 2024 Salcher-Konrad M et al. *JAMA Network Open*

Ziff 2015	372	Placebo or no treatment	Other objective outcome	Cardiovascular system	Moderate median risk of bias	Moderate median risk of bias	2000-2009	Moderate (score of 7-9 of 12)	First RCT published before NRS	No	Yes
Zuo 2015	373	Active	Other objective outcome	Anti-infective for systemic use	Moderate median risk of bias	Low median risk of bias	2010 and later	High (score of 10-12 of 12)	NRS published before first RCT	No	No

eTable 2. Results for Measures of Discrepancy Between Nonrandomized Studies and RCTs

	**Measure of discrepancy between non-randomized studies and RCTs**					
	Summary OR twice as favorable for one study type vs. the other (frequency (%))	Summary OR is 50% more favorable for one study type vs. the other (frequency (%))	Summary OR is 10% more favorable for one study type vs. the other (frequency (%))	Discrepancy in summary OR beyond chance (frequency (%))	Meta-analyses with different statistical conclusions (frequency (%))	ROR (95% CrI)
**Full sample (n=346 MA)**	121 (35.0)	237 (68.5)	319 (92.2)	54 (15.6)	130 (37.6)	0.95 (0.89–1.02)
NRS study type:						
**Experimental NRS (n=122 MA)**	55 (45.1%)	95 (77.9%)	117 (95.9%)	31 (25.4%)	42 (34.4%)	0.81 (0.67–0.97)
**Observational NRS (n=227 MA)**	40 (17.6%)	149 (65.6%)	207 (91.2%)	31 (13.7%)	89 (39.2%)	0.98 (0.87–1.06)
Outcome type:						
**Mortality outcome (n=59 MA)**	13 (22.0%)	44 (74.6%)	54 (91.5%)	11 (18.6%)	28 (47.5%)	0.95 (0.84–1.08)
**Other objective outcome (n=161 MA)**	50 (31.1%)	104 (64.6%)	148 (91.9%)	32 (19.9%)	56 (34.8%)	0.95 (0.85–1.06)
**Subjective outcome (n=126 MA)**	50 (39.7%)	89 (70.6%)	117 (92.9%)	37 (29.4%)	46 (36.5%)	0.97 (0.84–1.09)
Comparator:						
**Active comparator (n=108 MA)**	40 (37.0%)	80 (74.1%)	102 (94.4%)	19 (17.6%)	32 (29.6%)	0.91 (0.76–1.07)
**Placebo / no treatment comparator (n=237 MA)**	43 (18.1%)	161 (67.9%)	218 (92.0%)	38 (16.0%)	97 (40.9%)	0.95 (0.88–1.04)
Matching quality of RCTs and NRS in meta-analysis:						

© 2024 Salcher-Konrad M et al. *JAMA Network Open*

**Good match RCT-NRS (n=111 MA)**	35 (31.5%)	83 (74.8%)	105 (94.6%)	24 (21.6%)	40 (36.0%)	0.91 (0.79–1.03)
**Moderate match RCT-NRS (n=166 MA)**	53 (31.9%)	107 (64.5%)	152 (91.6%)	37 (22.3%)	57 (34.3%)	0.98 (0.87–1.12)
**Poor match RCT-NRS (n=69 MA)**	19 (27.5%)	47 (68.1%)	62 (89.9%)	19 (27.5%)	33 (47.8%)	0.96 (0.87–1.08)
Methodological quality of source meta-analyses:						
**Top journals only (n=118 MA)**	48 (40.7%)	80 (67.8%)	105 (89.0%)	27 (22.9%)	45 (38.1%)	0.88 (0.76–1.03)
**Cochrane reviews only (n=78 MA)**	36 (46.2%)	58 (74.4%)	72 (92.3%)	16 (20.5%)	29 (37.2%)	0.83 (0.65–1.03)
Timing of evidence generation:						
**NRS published before first RCT (n=146 MA)**	53 (36.3%)	104 (71.2%)	137 (93.8%)	31 (21.2%)	50 (34.2%)	0.95 (0.83–1.08)

Abbreviations: CrI, credible interval; MA, meta-analyses; NRS, non-randomized study(ies); OR, odds ratio; RCT, randomized controlled trial(s); ROR, ratio of odds ratios

eFigure. Results From Additional Subgroup Analyses for Study-Level Characteristics

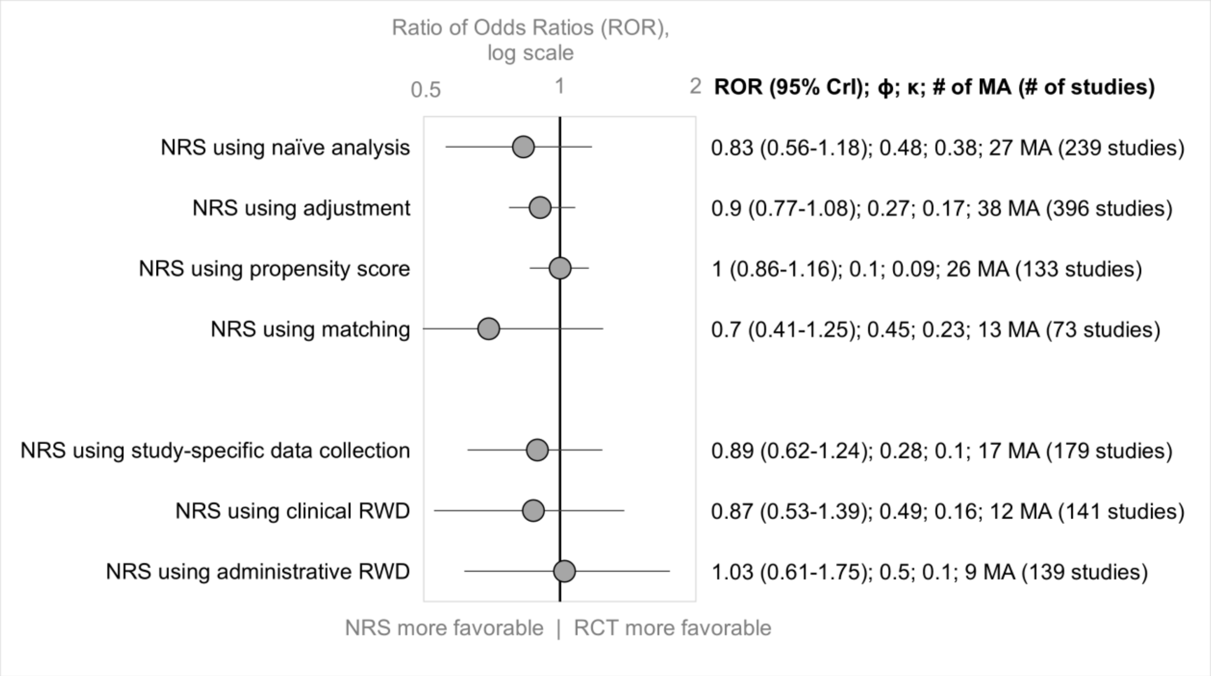

Legend:

Abbreviations: MA, meta-analyses; NRS, non-randomized studies; RCT, randomized controlled trials; ROR, ratio of odds ratios; RWD, real-world data.

Figure shows ratio of odds ratios (ROR) comparing effect estimates obtained from non-randomized studies to effect estimates obtained from randomized studies, and heterogeneity parameters (φ, between-meta-analysis heterogeneity; κ, increase in within-meta-analysis heterogeneity).

© 2024 Salcher-Konrad M et al. *JAMA Network Open*
