# Supplementary material for: Treatment Effects in Randomized and Nonrandomized Studies of Pharmacological Interventions: A Meta-Analysis
Source: JAMA Netw Open. 2024 Sep 27;7(9):e2436230. doi: 10.1001/jamanetworkopen.2024.36230 (PMC11437387; doi:10.1001/jamanetworkopen.2024.36230)
Supplement: Supplement 2. — Data Sharing Statement [file jamanetwopen-e2436230-s002.pdf]

Data Sharing Statement

Salcher-Konrad. Treatment Effects in Randomized and Nonrandomized Studies of Pharmacological Interventions. *JAMA Netw Open*. Published September 27, 2024. doi:10.1001/jamanetworkopen.2024.36230

Data
Data available: Yes
Data types: Data (not involving human participants)
How to access data: All raw data used in this study are publicly available under the following link: 10.5281/zenodo.4958221
When available: With publication

Supporting Documents
Document types: None

Additional Information
Who can access the data: N/A
Types of analyses: N/A
Mechanisms of data availability: N/A
